# Synthesis of Five‐Porphyrin Nanorings by Using Ferrocene and Corannulene Templates

**DOI:** 10.1002/anie.201602909

**Published:** 2016-05-23

**Authors:** Pengpeng Liu, Yutaka Hisamune, Martin D. Peeks, Barbara Odell, Juliane Q. Gong, Laura M. Herz, Harry L. Anderson

**Affiliations:** ^1^ Department of ChemistryUniversity of OxfordChemistry Research LaboratoryOxfordOX1 3TAUK; ^2^ Department of PhysicsUniversity of OxfordClarendon LaboratoryParks RoadOxfordOX1 3PUUK

**Keywords:** corannulene derivatives, ferrocene derivatives, macrocycles, strained rings, templated synthesis

## Abstract

The smallest and most strained member of a family of π‐conjugated cyclic porphyrin oligomers was synthesized by using pentapyridyl templates based on ferrocene and corannulene. Both templates are effective for directing the synthesis of the butadiyne‐linked cyclic pentamer, despite the fact that the radii of their N5 donor sets are too small by 0.5 Å and 0.9 Å, respectively (from DFT calculations). The five‐porphyrin nanoring exhibits a structured absorption spectrum and its fluorescence extends to 1200 nm, reflecting strong π conjugation and Herzberg–Teller vibronic coupling.

Strained π systems, such as picotubes,^1^ nanohoops,^2^ bowls,^3^ cages,^4^ and helicences,^5^ have attracted increasing attention because of their remarkable electronic structures and properties. Previously, we have investigated the synthesis of butadiyne‐linked nanorings consisting of 6–50 porphyrin units.^6^ Herein, we present the synthesis of the smallest and most strained macrocycle in this family, the five‐porphyrin nanoring ***c*** **‐P5**. In this work, we compared the ability of two pentadentate templates to direct the formation of this cyclic pentamer: **T5_Fc_** and **T5**_**cor**_, which are based on ferrocene and corannulene cores, respectively (Figure 1, Scheme 1, and Scheme 2).

**Figure 1:**
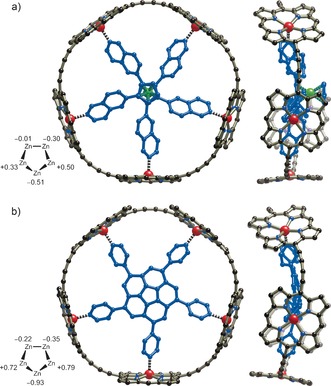
Two orthogonal views of the DFT‐calculated geometries of a) ***c*** **‐P5⋅T5_Fc_** and b) ***c*** **‐P5⋅T5**_**cor**_, showing the deviations of the Zn atoms from the Zn5 mean planes in Å. (B3LYP/6‐31G* with D3 dispersion correction; *meso*‐aryl groups and the PO(*t*‐Bu)_2_ were omitted to simplify the calculations.)

**Scheme 1:**
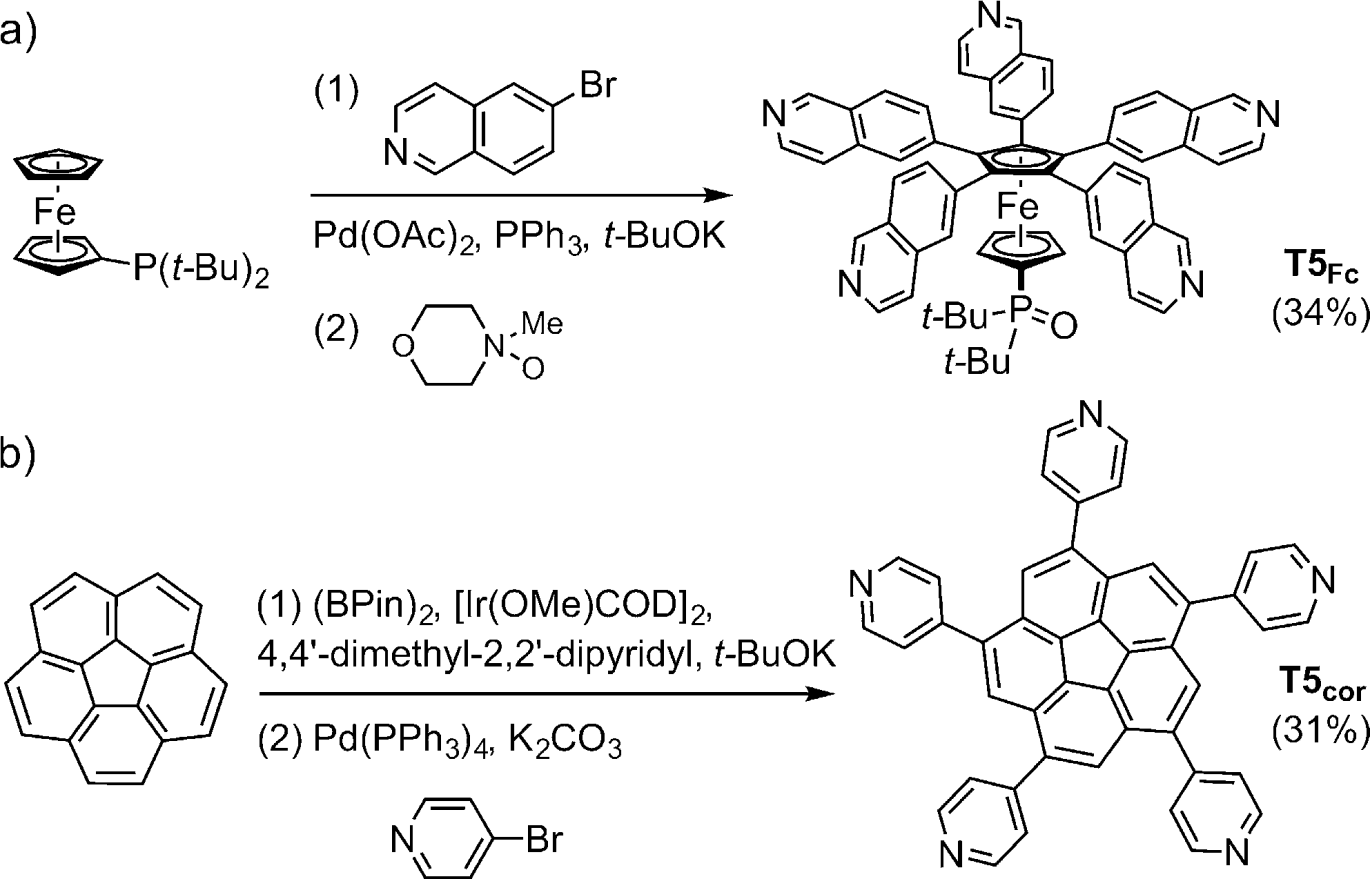
Synthesis of the templates **T5_Fc_** and **T5**_**cor**_, with overall yields.

**Scheme 2:**
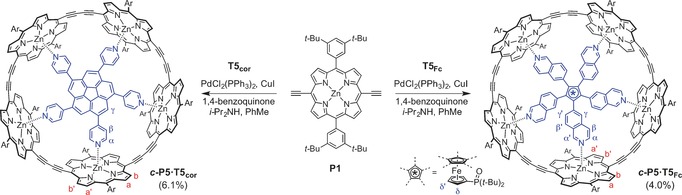
Template‐directed synthesis of ***c*** **‐P5⋅T5_Fc_** and ***c*** **‐P5⋅T5**_**cor**_.

The design of these templates started with a computational study. Density functional theory (DFT) geometry optimizations using Gaussian09/D.01 at the B3LYP/6‐31G* level^7^ with Grimme's D3 dispersion correction^8^ indicate that both templates are too small for the cavity of ***c*** **‐P5**. The radii of the N5 donor sets (measured to the centroid of the five N atoms) are 7.73 and 7.37 Å for **T5_Fc_** and **T5**_**cor**_, respectively. The optimal N5 radius for binding ***c*** **‐P5**, computed by multiple methods, is 8.27±0.07 Å.^9^ The corannulene core of **T5**_**cor**_ adopts the usual bowl conformation, but upon complexation in ***c*** **‐P5⋅T5**_**cor**_, the bowl becomes flatter, thereby extending the radius of the N5 donor set by 0.17 Å to 7.54 Å. All five zinc centers are in the same plane in the ligand‐free ***c*** **‐P5** nanoring, whereas they distort into an envelope conformation reminiscent of cyclopentane when ***c*** **‐P5** binds **T5_Fc_** and **T5**_**cor**_ (Figure 1). The better fit of **T5_Fc_** for ***c*** **‐P5**, compared with **T5**_**cor**_, is reflected in the deviations from planarity of the Zn5 acceptor set: the root‐mean‐square deviation from the mean plane is 0.43 Å in ***c*** **‐P5⋅T5_Fc_** versus 0.67 Å in ***c*** **‐P5⋅T5**_**cor**_. Although these calculations demonstrated that the geometries of the templates are not ideal, we decided to test whether they could direct the synthesis of ***c*** **‐P5**, and this approach turned out to be successful.

Both templates were prepared through transition‐metal‐catalyzed C−H activation (Scheme 1). The ferrocene‐based template **T5_Fc_** was synthesized by phosphine‐activated palladium‐catalyzed aryl–aryl coupling,^10^ while **T5**_**cor**_ was synthesized from corannulene by iridium‐catalyzed borylation,3a, 11 followed by Suzuki coupling. Both templates are effective in directing the palladium‐catalyzed oxidative coupling of porphyrin monomer **P1** to give the five‐porphyrin nanoring in yields of 4.0 % for ***c*** **‐P5⋅T5_Fc_** and 6.1 % for ***c*** **‐P5⋅T5**_**cor**_ (Scheme 2). We also synthesized a version of ***c*** **‐P5** with different solubilizing aryl groups (OC_8_H_17_ rather than *t*‐Bu; see the Supporting Information). GPC analysis shows that the main byproducts in these reactions are larger linear and cyclic porphyrin oligomers (see the Supporting Information). The yield of ***c*** **‐P5** is consistently higher when using **T5**_**cor**_ rather than **T5_Fc_** as the template, for both porphyrin monomers. Addition of excess pyridine quantitatively displaces both templates from their complexes, yielding the template‐free nanorings. The template complexes can be regenerated immediately by adding **T5_Fc_** or **T5**_**cor**_ to a solution of ***c*** **‐P5**.

The ^1^H NMR spectra of nanoring complexes ***c*** **‐P5⋅T5_Fc_** and ***c*** **‐P5⋅T5**_**cor**_ (Figure 2) were fully assigned by using 2D correlation techniques (see the Supporting Information). As expected, the template protons are shielded by the porphyrin ring current; for example the α‐pyridine protons are shifted by Δ*δ*(=*δ*_H,**T5**_−*δ*_H,***c*****‐P5⋅T5**_)=6.45 ppm in both complexes (see list of Δ*δ* values in the Supporting Information).

**Figure 2:**
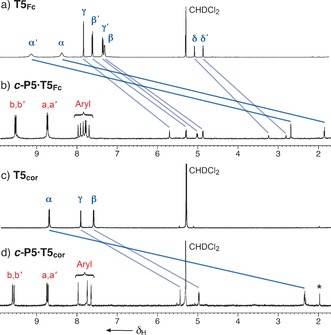
Partial ^1^H‐NMR spectra of a) **T5_Fc_**, b) ***c*** **‐P5⋅T5_Fc_** c) **T5**_**cor**_ and d) ***c*** **‐P5⋅T5**_**cor**_. All spectra were recorded in CD_2_Cl_2_, 298 K, 500 MHz. Spectrum (b) is diffusion‐edited to remove overlapping solvent peaks. An impurity signal in spectrum (d) is indicated by *.

The distortions in the DFT‐calculated geometries (Figure 1) are not reflected in the ^1^H NMR spectra, presumably because there is rapid interconversion between five degenerate envelope conformations for each complex. The symmetry of the ferrocene‐based template **T5_Fc_** is effectively *C*_5v_ on the NMR timescale, owing to fast rotation of the isoquinoline substituents and of the phosphine oxide. This symmetry is retained in ***c*** **‐P5⋅T5_Fc_** and the rims of the nanoring become non‐equivalent, thereby resulting in four β‐pyrrole doublets (a, a′, b and b′; Scheme 2 and Figure 2) and six aromatic aryl signals because each porphyrin has two non‐equivalent faces.

The corannulene template **T5**_**cor**_ is chiral, but racemization through bowl‐to‐bowl inversion is expected to be fast at room temperature^12^ and the ***c*** **‐P5⋅T5**_**cor**_ complex has *C*_5h_ symmetry on the ^1^H NMR timescale, which explains why there are four (rather than eight) β‐pyrrole doublets (a, a′, b and b′) and three (rather than six) signals for the aryl protons. As mentioned above, DFT calculations (Figure 1 b) indicate that the **T5**_**cor**_ template is stretched when it binds ***c*** **‐P5⋅T5**_**cor**_, flattening the bowl and reducing the barrier to bowl‐to‐bowl inversion, but we were unable to test this prediction because the complex is not sufficiently soluble for a low‐temperature NMR study.

The NIR absorption spectra of ***c*** **‐P5⋅T5_Fc_**, ***c*** **‐P5⋅T5**_**cor**_, and template‐free ***c*** **‐P5** all exhibit sharp finger patterns (Figure 3). This behavior is similar to that of the six‐porphyrin ring ***c*** **‐P6**,6b whereas larger macrocycles of this type do not have structured Q bands.^13^ The absorption spectrum of template‐free ***c*** **‐P5** is similar to those of ***c*** **‐P5⋅T5_Fc_** and ***c*** **‐P5⋅T5**_**cor**_, thus indicating that ***c*** **‐P5** is shape‐persistent and that its conformation is not strongly perturbed by the templates; only a slight broadening arises from the increased flexibility of ***c*** **‐P5** in the absence of template. The fluorescence spectra of the three compounds extend far into the NIR region (Figure 3), like that of ***c*** **‐P6**.6b The fluorescence quantum yields, decay times, and radiative rate constants are compared with those of ***c*** **‐P6** and ***c*** **‐P6⋅T6** in Table 1.^13^ The low fluorescence quantum yields of all these compounds result from the fact that S_1_–S_0_ transitions are only weakly allowed in circular π systems.6b, 13 Binding of either **T5_Fc_** or **T5**_**cor**_ to ***c*** **‐P5** reduces the radiative rate and the fluorescence quantum yield. Taken in isolation, the low fluorescence quantum yield of ***c*** **‐P5⋅T5_Fc_** might be viewed as evidence for photoinduced electron transfer involving the redox‐active ferrocene core. However, the fact that ***c*** **‐P5⋅T5_Fc_** and ***c*** **‐P5⋅T5**_**cor**_ have similar fluorescence quantum yields implies that this is a consequence of the regular circular geometry of the complexes, which suppresses the symmetry‐breaking vibrations required for Herzberg–Teller coupling.^13^

**Figure 3:**
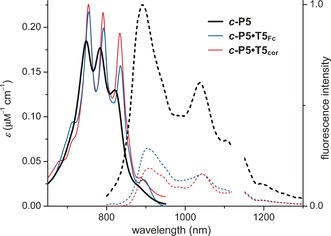
NIR absorption (*ɛ*, solid lines) and fluorescence spectra (dashed lines) of ***c*** **‐P5** (black), ***c*** **‐P5⋅T5_Fc_** (blue), and ***c*** **‐P5⋅T5**_**cor**_ (red) in toluene containing 1 % pyridine at 298 K. The fluorescence intensity is normalized such that the areas of the peaks are proportional to their quantum yields. Data at 1116–1148 nm are not shown due to overlap with solvent signals.

**Table 1:** Fluorescence quantum yields *Φ*_F_, fluorescence decay times *τ*, and radiative rate constants *k*_R_.^[a]^

Compound	*Φ*_F_^[b]^	*τ* [ns]	*k*_R_ [ns^−1^]^[c]^
***c*** **‐P5**	3.1 %	0.45	0.067
***c*** **‐P5⋅T5_Fc_**	0.89 %	0.42	0.021
***c*** **‐P5⋅T5**_**cor**_	0.61 %	0.37	0.016
***c*** **‐P6** ^[d]^	1.5 %	0.49	0.031
***c*** **‐P6⋅T6** ^[d]^	0.42 %	0.25	0.017

[a] Solvent: toluene with 1 % pyridine, 298 K. [b] Quantum yields measured as described in Ref. 13 using linear porphyrin hexamer ***l*** **‐P6** as a standard (*Φ*_F_=28 %). [c] *k*_R_=*Φ*_F_/τ. [d] Data for ***c*** **‐P6** and ***c*** **‐P6⋅T6** from Ref. 13.

The formation constants (*K*_f_) of the nanoring–template complexes reflect how well the templates fit the cavity of the five‐porphyrin nanoring. The nanoring–template complexes ***c*** **‐P5⋅T5_Fc_** and ***c*** **‐P5⋅T5**_**cor**_ are too stable for their formation constants to be determined by direct titration, so we measured *K*_f_ by displacing the templates with pyridine, giving log *K*_f_ values of 29.3±0.2 and 28.5±0.1 for ***c*** **‐P5⋅T5_Fc_** and ***c*** **‐P5⋅T5**_**cor**_, respectively (see the Supporting Information). The chelate cooperativity of complex formation is quantified by the effective molarity (EM), calculated from the formation constant (*K*_f_), the statistical factor (*K*_σ_) of the complex, and the corresponding microscopic binding constant (*K*_1_) for the ligand site (isoquinoline for **T5_Fc_** and pyridine for **T5**_**cor**_). The geometric average of the four effective molarities ($\bar{\mathrm{EM}}$ ) of the five‐coordinate complex can be calculated from Equation (1).

(1) EM‾=4KfKσK51

The effective molarities for ***c*** **‐P5⋅T5_Fc_** and ***c*** **‐P5⋅T5**_**cor**_ are $\bar{\mathrm{EM}}$ =41±9 m and $\bar{\mathrm{EM}}$ =36±5 m, respectively. While being higher than the values in many supramolecular systems,^14^ these effective molarities are lower than those for the corresponding six‐porphyrin ring ***c*** **‐P6**, either with a rigid **T6** template ($\bar{\mathrm{EM}}$ =126±5 m)^15^ or with a flexible cyclodextrin‐based template ($\bar{\mathrm{EM}}$ =74±20 m),6d which reflects the poor size complementarity of **T5_Fc_** and **T5**_**cor**_ for ***c*** **‐P5**.

In conclusion, templates based on ferrocene and corannulene can be used to direct the synthesis of the five‐porphyrin nanoring ***c*** **‐P5**, which has a diameter of 2.1 nm. The corannulene‐based template gives a higher yield of ***c*** **‐P5**, despite being too small and having a lower affinity for ***c*** **‐P5**. The lack of correlation between the size of the template and its ability to direct the formation of ***c*** **‐P5** may indicate that the transition state for template‐directed coupling is smaller than the final product. The five‐porphyrin nanoring exhibits highly structured absorption and fluorescence spectra and a low radiative rate, thus indicating that emission is strongly suppressed due to the high rotational symmetry of the lowest excited state, with the majority of the fluorescence arising from dynamic symmetry breaking through Herzberg–Teller coupling. This work demonstrates that perfect size‐complementarity is not essential in template‐directed synthesis, and it illustrates how templates can be used to synthesize strained π‐conjugated macrocycles.^16^

## Supporting information

As a service to our authors and readers, this journal provides supporting information supplied by the authors. Such materials are peer reviewed and may be re‐organized for online delivery, but are not copy‐edited or typeset. Technical support issues arising from supporting information (other than missing files) should be addressed to the authors.

Supplementary
