# Supplementary material for: Synthesis of Five‐Porphyrin Nanorings by Using Ferrocene and Corannulene Templates
Source: Angew Chem Int Ed Engl. 2016 May 23;55(29):8358–62. doi: 10.1002/anie.201602909 (PMC5089564; doi:10.1002/anie.201602909)
Supplement: Supplementary file 1 — Supplementary [file ANIE-55-8358-s001.pdf]

Angewandte Chemie
Eine Zeitschrift der Gesellschaft Deutscher Chemiker

Supporting Information

Synthesis of Five-Porphyrin Nanorings by Using Ferrocene and Corannulene Templates

*Pengpeng Liu, Yutaka Hisamune, Martin D. Peeks, Barbara Odell, Juliane Q. Gong, Laura M. Herz, and Harry L. Anderson**

anie_201602909_sm_miscellaneous_information.pdf

Table of Contents

A. General Methods S2

B. Synthetic Procedures S3

B1. Known Compounds S3

B2. Novel Compounds S4

C. Spectra Confirming Identity and Purity of New Compounds S10

C1. Full Spectra S10

C2. Characterization of ***c-P5·T5***$_{Fc}$ S25

C3. Characterization of ***c-P5***$_{OOct}$***·T5***$_{Fc}$ S29

C4. Characterization of ***c-P5·T5***$_{cor}$ S31

C5. The Shielding Effects of Porphyrin Nanoring S33

C6. GPC Traces of ***c-P5·T5***$_{Fe}$ *and* ***c-P5·T5***$_{cor}$ *Crude Reaction Mixtures* S33

D. Fluorescent Properties S35

D1. Time-Resolved Photoluminescence Spectroscopy S35

D2. Quantum Yields and Radiative Rates S36

E. Effective Molarity Measurement S37

E1. Titrations of Monodentate Ligands with ***c-P5*** *(Reference Titrations)* S37

E2. Denaturation Titration of ***c-P5·T5***$_{Fc}$ S40

E3. Denaturation Titration of ***c-P5·T5***$_{cor}$ S42

E4. Calculation of Effective Molarities S43

F. Computational details S45

G. References S47

A. General Methods

Dry toluene was obtained by passing the solvent through a column of alumina, under nitrogen. Diisopropylamine (DIPA) was distilled from CaH_2 and kept over activated molecular sieves (3 Å, 8–12 mesh). Unless specified otherwise, all other solvents were used as commercially supplied.

Flash chromatography was carried out on silica gel 60 under positive pressure. Analytical thin-layer chromatography was carried out on aluminum-backed silica gel 60 F254 plates. Visualization was achieved using UV light when necessary. Gel-permeation chromatography (GPC) was performed on a line of JAIGEL 3H (20 × 600 mm) and JAIGEL 4H (20 × 600 mm) columns in the solvent of toluene/pyridine (100/1, *v*/*v*) and the flow rate of 3.5 mL/min. Analytical gel permeation chromatography (GPC) was performed on a JAIGEL H-P pre-column, a JAIGEL 3H-A (8 × 500 mm) and a JAIGEL 4H-A column (8 × 500 mm) in series with toluene/pyridine 100/1 as eluent or on two PLgel 3 µm Mixed-E columns (2 × 300 mm length, 7.5 mm diameter) and two PLgel 5 µm Mixed-D columns (3 × 300 mm length, 7.5 mm diameter) in series with THF as an eluent.

All UV-vis-NIR spectra were recorded in solution using a Perkin-Lambda 20 spectrometer (1 cm path length quartz cell). NMR data were collected on Bruker AVANCE AVIIIHD 400 and AVII 500 spectrometers. Samples were dissolved in dry $CDCl_3$ or CD_2Cl_2 and characterized by ^1H, COSY, edited-HSQC and ^{13}C NMR and 2D NOESY spectra (with mixing times of 300 ms) using the standard Bruker pulse programs. Diffusion-edited spectra were collected using the standard Bruker pulse program, ledbpgp2s1d sequence for diffusion measurements using stimulated echo and LED (longitudinal eddy current delay) and bipolar gradient pulses for diffusion. With parameters big DELTA 100 ms, an eddy current delay of 5 ms and gradient strength 2–40% depending on the attenuation of solvent peaks. For each data set, 256 or 512 transients were acquired using recovery times of 3 s. Data were processed typically 32K data points using line broadening of 0.5 Hz.

^1H, ^{13}C and ^{31}P NMR spectra are reported in ppm; coupling constants are given in Hertz and are quoted to the nearest 0.1 Hz. Spectral referencing was based absolute spectrometer frequencies calculated relative to TMS scaling factors for the relevant nuclei, ^1H, ^{13}C and ^{31}P NMR ^{31}P NMR spectra are referenced with respect to H_3PO_4 at 0 ppm.

ESI mass spectra were carried out either using Fisons Platform or Micromass LCT spectrometer. MALDI-TOF mass spectra were carried out using Waters MALDI Micro MX spectrometer.

Steady-state absorption and time-integrated PL spectra at 298 K were recorded using a PerkinElmer Lambda 1050 UV-vis-NIR spectrometer and a Horiba FluoroLog fluorimeter respectively.

B. Synthetic Procedures

B1. Known Compounds

Di-*tert*-butyl-phosphinoferrocene tetrafluoroboric acid complex[S1] (**1**, starting material for **T5$_{Fc}$**), pentakis-BPin corannulene[S2] (**2**, starting material for **T5$_{cor}$**) and porphyrin monomers **P1**[S3, S4] were synthesized using published procedures. Our slightly modified procedure for preparation of pentakis-Bpin corannulene (**2**) is detailed here:

Pentakis-BPin corannulene, 2

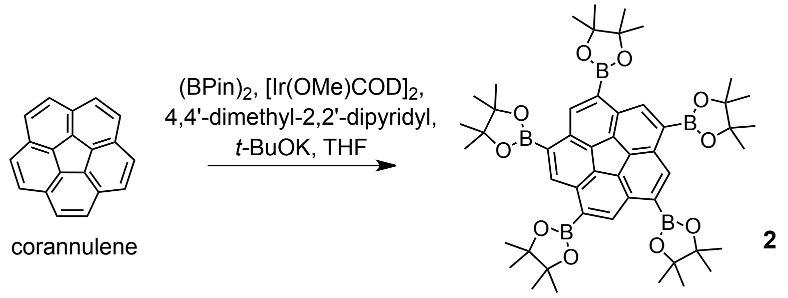

Scheme S1. The synthesis of pentakis-Bpin corannulene **2**

A pressure vessel was charged with $[Ir(OMe)COD]_2$ (53 mg, 0.08 mmol), 4,4'-dimethyl-2,2'-dipyridyl (30 mg, 0.16 mmol), $(BPin)_2$ (528 mg, 2.08 mmol) and *t*-BuOK (5 mg, 0.04 mmol), all as dry solids. THF (0.4 mL) was added by syringe and the mixture was heated at 50 °C for 5 min under N_2. A dark brown-red solution formed. The vessel was charged with corannulene (100 mg, 0.40 mmol), purged with N_2, sealed and stirred at 85 °C for 4 days. The reaction mixture was cooled, opened to the air and immediately diluted with DCM (5 mL), then quenched by dropwise addition of water (3 mL). The layers were separated and the aqueous layer was thoroughly extracted with DCM. The combined organic layers were washed with water and brine, and dried over magnesium sulfate. The filtrate was concentrated to dryness on a rotary evaporator to give a dark red-brown oily mixture. Methanol (8 mL) was added and the mixture was sonicated for 10 min, until the dark mixture separated into a dark solution and an off-white precipitate. The precipitate was collected by vacuum filtration and washed with methanol to yield **2** as white solid (192 mg, 55%).

B2. Novel Compounds

T5$_{Fc}$

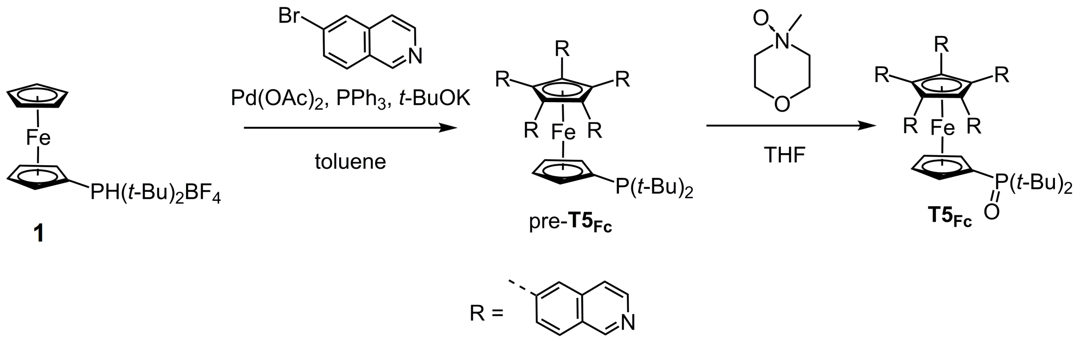

Scheme S2. The synthesis of **T5$_{Fc}$**

Compound **1** (0.12 g, 0.28 mmol), 6-bromoisoquinoline (0.70 g, 3.3 mmol), *t*-BuOK (0.30 g, 2.6 mmol), $Pd(OAc)_2$ (6.0 mg, 0.027 mmol) and PPh_3 (33 mg, 0.13 mmol) were dissolved in dry toluene (12 mL). The mixture was degassed using the freeze-pump-thaw method and was then stirred vigorously at 107 °C under argon for 2 days. The mixture was cooled and filtered through Celite and the solvent was evaporated under vacuum. The residue was separated with column chromatography (SiO_2) using DCM/EtOH (80/11 gradually increasing polarity to 5/1, *v/v*) as the eluent. Pre-**T5$_{Fc}$** was obtained as the last dark red band (0.11 g, 40%). pre-**T5$_{Fc}$**: ^{1}H NMR (400 MHz, $CDCl_3$): δ = 9.19 (s, br, 5H), 8.43 (s, br, 5H), 7.69 (s, 5H), 7.65 (d, *J* = 8.5 Hz, 5H), 7.45 (dd, *J* = 8.5 Hz, *J* = 1.0 Hz, 5H), 7.26 – 7.24 (m, 5H), 4.78 (s, 2H), 4.64 (t, *J* = 1.6 Hz, 2H), 1.04 (d, *J* = 11.2 Hz, 18H); ^{13}C NMR (100 MHz, $CDCl_3$): δ = 152.2, 143.6, 137.4, 135.0, 131.8, 130.0, 126.5, 88.1, 78.4, 78.3, 76.6, 33.6 (d, *J* = 25 Hz), 31.1 (d, *J* = 13 Hz); ^{31}P{^{1}H} NMR ($CDCl_3$, 162 MHz): δ = 24.91.

Pre-**T5$_{Fc}$** (0.10 g, 0.10 mmol) was dissolved in THF (20 mL). *N*-Methylmorpholine *N*-oxide monohydrate (15 mg, 0.12 mmol) was added and the mixture was stirred at room temperature overnight. The solvent was evaporated and the residue was dissolved in DCM and the solution was washed with water and dried with $MgSO_4$. The DCM solvent was evaporated and the residue was precipitated by layered diffusion of hexane into its solution in benzene. The product was obtained as bright red solid (84 mg, 84%). **T5$_{Fc}$**: ^{1}H NMR (400 MHz, $CDCl_3$): *δ* = 9.14 (s, 5H), 8.41 (d, *J* = 5.8 Hz, 5H), 7.79 (s, 5H), 7.65 (d, *J* = 8.4 Hz, 5H), 7.37 (dd, *J* = 8.4 Hz, 1.6 Hz, 5H), 7.29 (d, *J* = 5.8 Hz, 5H), 5.08 (d, *J* = 1.6 Hz, 2H), 4.84 (d, *J* = 1.6 Hz, 2H), 0.96 (d, *J* = 14.2 Hz, 18H); ^{13}C NMR (100 MHz, $CDCl_3$): δ = 152.3, 143.8, 136.8, 135.3, 131.1, 130.4, 127.4, 127.0, 120.4, 88.8, 79.3, 79.2, 77.9 (d, *J* = 7.9 Hz), 77.7, 77.6, 37.0 (d, *J* = 60.7 Hz), 27.1; ^{31}P{^{1}H} NMR ($CDCl_3$, 162 MHz): δ = 62.27; MALDI-TOF MS+: *m/z* 981.22 ([M]$^+$, $C_{63}H_{52}FeN_5PO$ requires: 981.33).

1,3,5,7,9-Penta(4-pyridyl)corannulene T5$_{cor}$

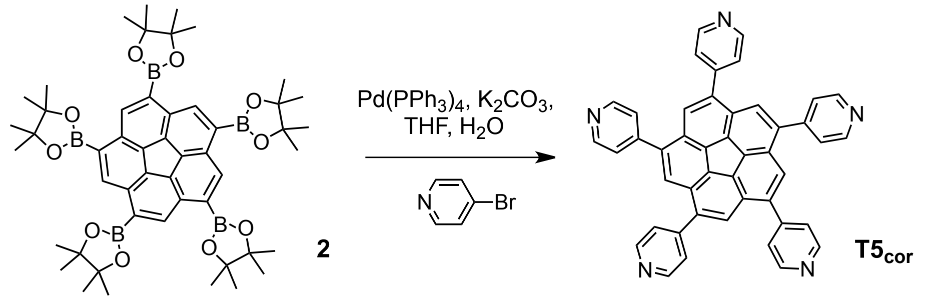

Scheme S3. The synthesis of **T5$_{cor}$**

Tetrakis(triphenylphosphine)-palladium (13 mg, 11 μmol) was added to a solution of compound **2** (20 mg, 23 μmol) in THF (0.9 mL). After addition of water (0.3 mL), K_2CO_3 (156 mg, 1.13 mmol) and 4-bromopyridine hydrochloride (88 mg, 0.45 mmol), the mixture was deoxygenated (vacuum freeze/thaw) and stirred at 70 °C for 10 days. Solvents were removed and the crude product was purified by column chromatography on flash silica gel (chloroform : methanol : triethylamine; gradient 100:0:0 to 100:5:0.5) to give the template as a pale yellow solid (8.2 mg, 57%). **T5$_{cor}$**: ^{1}H NMR (400 MHz, $CDCl_3$): δ = 9.80 (d, J = 5.8 Hz, 10H), 7.92 (s, 5H), 7.62 (d, J = 5.8 Hz, 10H); ^{13}C NMR (100 MHz, $CDCl_3$): δ = 150.58, 146.32, 140.37, 136.22, 128.72, 126.62, 124.44; MALDI-TOF MS+: *m/z* 635.85 ([M]$^+$, $C_{45}H_{25}N_5$ requires: 635.21); HRMS (ESI-TOF) *m/z* 636.21795 ([M+H]$^+$, $C_{25}H_{46}N_5$ 636.21837). UV-vis (toluene): λ_{max} (ε) 288 (1.3 × 10^4), 319 (1.1 × 10^4).

***c*-P5·T5$_{Fc}$**

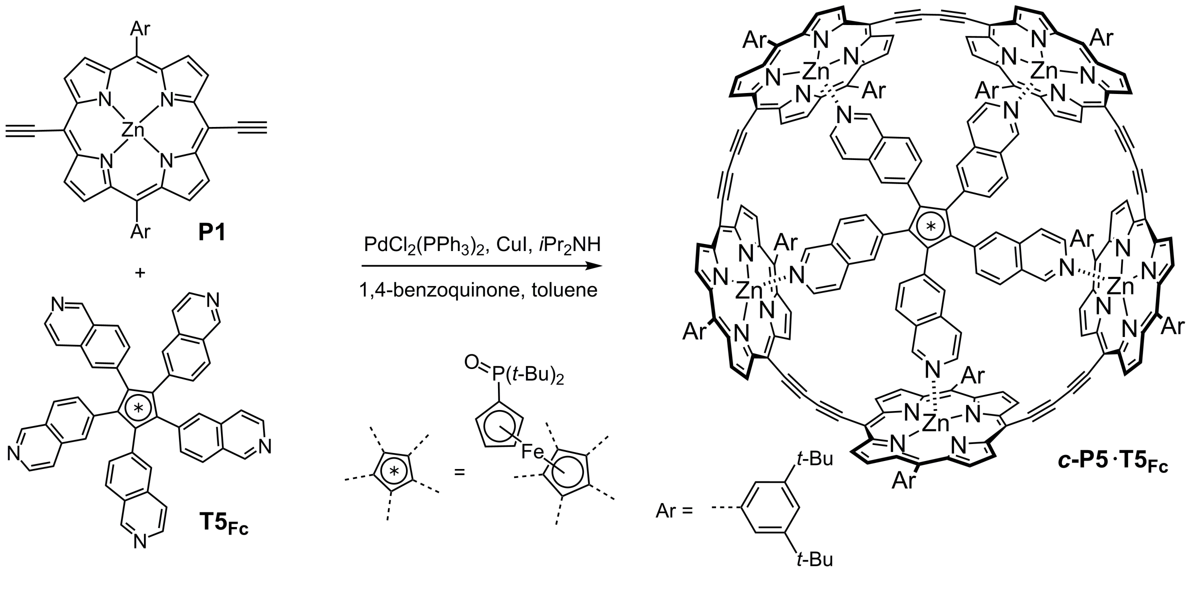

Scheme S4. The synthesis of ***c*-P5·T5$_{Fc}$**

P1 (31 mg, 38 μmol) and **T5$_{Fc}$** (7.5 mg, 7.7 μmol) were dissolved in toluene (700 mL). DIPA (7.5 mL) was added and the solution was stirred for 3 h. $PdCl_2(PPh_3)_2$ (70 mg, 0.10 mmol), CuI (75 mg, 0.19 mmol) and 1,4-benzoquinone (90 mg, 0.83 mmol) were added into the solution and the mixture was stirred at room temperature overnight. The mixture was filtered through basic Al_2O_3 and the solvent was evaporated. The residue was separated

through size-exclusion column (Biobeads SX-1, 35 × 300 mm) with pure toluene and the second band was collected. The first (major) band was larger porphyrin nanorings and linear oligomers. The solvent was removed under vacuum and the residue was precipitated with DCM/MeOH to give the product as black solid (1.52 mg, 4.0%). ***c*-P5·T5$_{\mathbf{Fc}}$**: ^{1}H NMR (500 MHz, CD_2Cl_2): 9.54 (d, *J* = 4.4 Hz, 10H), 9.52 (d, *J* = 4.4 Hz, 10H), 8.75 (d, *J* = 4.4 Hz, 10H), 8.73 (d, *J* = 4.4 Hz, 10H), 7.97 (t, *J* = 1.5 Hz, 5H), 7.91 (t, *J* = 1.5 Hz, 5H), 7.85 (t, *J* = 1.5 Hz, 5H), 7.79 (t, *J* = 1.5 Hz, 5H), 7.78 (t, *J* = 1.5 Hz, 5H), 7.70 (t, *J* = 1.5 Hz, 5H), 5.70 (s, 5H), 5.30 (d, *J* = 9.0 Hz, 5H), 5.01 (d, *J* = 9.0 Hz, 5H), 4.87 (d, *J* = 6.2 Hz, 5H), 3.24 (s, 2H), 2.80 (s, 2H), 2.69 (s, 5H), 1.86 (d, *J* = 6.2 Hz, 5H), 1.54 – 1.36 (m, 180H), -0.25 (d, *J* = 13.8 Hz, 18H); ^{31}P{^{1}H} NMR (CD_2Cl_2, 162 MHz): δ = 59.78; MALDI-TOF MS+: *m/z* 4953 ($[M]^+$, $C_{323}H_{302}FeN_{25}Zn_5PO$ requires: 4963), 3978 ([M-**T5$_{\mathbf{Fc}}$**]$^+$, $C_{260}H_{250}N_{20}Zn_5$ requires: 3982); UV-vis-NIR-NIR (toluene): λ_{max} (ε) 431 (2.5 × 10^5), 494 (4.0 × 10^5), 756 (2.2 × 10^5), 794 (2.2 × 10^5), 837 (1.8 × 10^5). (Note: ***c*-P5·T5$_{\mathbf{Fc}}$** has poor stability and trace impurities appear after a week of storage at –20 °C, either as a solid or in solution, as monitored by ^{1}H NMR.)

***c*-P5**

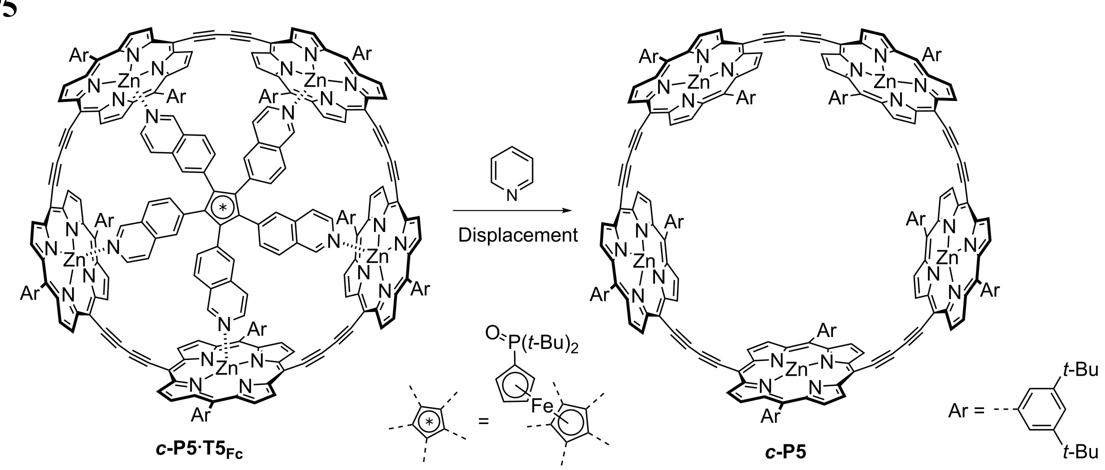

Scheme S5. The preparation of ***c*-P5**

***c*-P5·T5$_{\mathbf{Fc}}$** (2.0 mg, 0.40 μmol) was dissolved in toluene/pyridine (2/1, *v/v*) and the solution was passed through a size-exclusion column (Biobeads SX-1, 10 × 300 mm) with toluene/pyridine (2/1, *v/v*). The major band was collected and the solvent was evaporated. The desired product was obtained as black solid (1.5 mg, 94%). ***c*-P5**: ^{1}H NMR (400 MHz, $CDCl_3$): δ = 9.49 (d, *J* = 4.5 Hz, 20H), 8.68 (d, *J* = 4.5 Hz, 20H), 7.81 (d, *J* = 1.6 Hz, 20H), 7.71 (d, *J* = 1.6 Hz, 10H), 1.44 (s, 180H); MALDI-TOF MS+: *m/z* 3974 ($[M]^+$, $C_{260}H_{250}N_{20}Zn_5$ requires: 3982); UV-vis-NIR (toluene): λ_{max} (*ε*) 430 (2.4 × 10^5), 489 (4.0 × 10^5), 719 (1.6 × 10^5), 747 (1.7 × 10^5).

***c*-P5$_{OOct}$·T5$_{Fc}$**

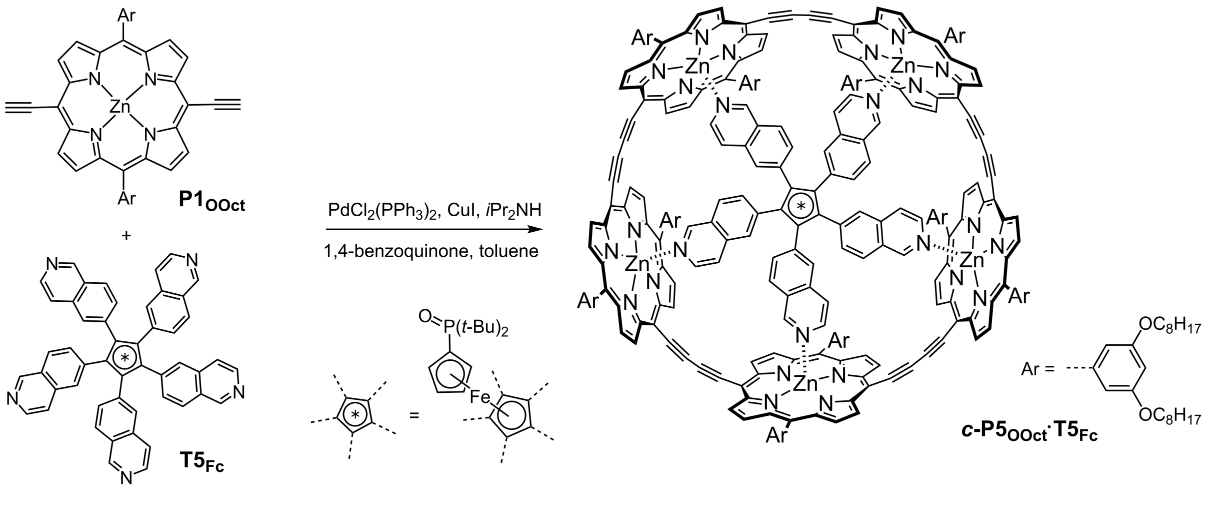

Scheme S6. The synthesis of ***c*-P5$_{OOct}$·T5$_{Fc}$**

P1$_{OOct}$ (42 mg, 38 μmol) and **T5$_{Fc}$** (7.5 mg, 7.7 μmol) were dissolved in toluene (700 mL). DIPA (7.5 mL) was added and the solution was stirred for 3 h. $PdCl_2(PPh_3)_2$ (70 mg, 0.10 mmol), CuI (75 mg, 0.19 mmol) and 1,4-benzoquinone (90 mg, 0.83 mmol) were added into the solution and the mixture was stirred at room temperature overnight. The mixture was filtered through basic Al_2O_3 and the solvent was evaporated. The residue was separated through size-exclusion column (Biobeads SX-1, 25 × 1000 mm) to remove low molecular weight impurities. The product was purified by recycling GPC (toluene/1% pyridine, *v/v*) to give the desired compound as black solid (0.86 mg, 1.7%). ***c*-P5$_{OOct}$·T5$_{Fc}$**: ^{1}H NMR (500 MHz, CD_2Cl_2): 9.53 (d, J = 4.4 Hz, 10H), 9.50 (d, J = 4.4 Hz, 10H), 8.80 (d, J = 4.4 Hz, 20H), 7.16 – 7.09 (m, 20H), 6.83 (t, J = 1.5 Hz, 5H), 6.81 (t, J = 1.5 Hz, 5H), 5.68 (s, 5H), 5.28 (d, J = 9.0 Hz, 5H), 5.01 (d, J = 9.0 Hz, 5H), 4.88 (d, J = 6.2 Hz, 5H), 4.10 – 4.00 (m, 40H), 3.29 (s, 2H), 2.81 (s, 2H), 2.71 (s, 5H), 1.84 – 0.70 (m, 300H), -0.24 (d, J = 13.8 Hz, 18H); ^{31}P{^{1}H} NMR (CD_2Cl_2, 162 MHz): δ = 60.14; MALDI-TOF MS+: *m/z* 6404 ([M]$^+$, $C_{403}H_{462}FeN_{25}Zn_5PO_{21}$ requires: 6405), 5422 ([M-**T5$_{Fc}$**]$^+$, $C_{340}H_{410}N_{20}O_{20}Zn_5$ requires: 5424); UV-vis-NIR (toluene): λ_{max} (ε) 431 (2.4 × 10^5), 494 (4.0 × 10^5), 749 (2.2 × 10^5), 785 (2.2 × 10^5), 828 (1.9 × 10^5).

***c*-P5·T5$_{cor}$**

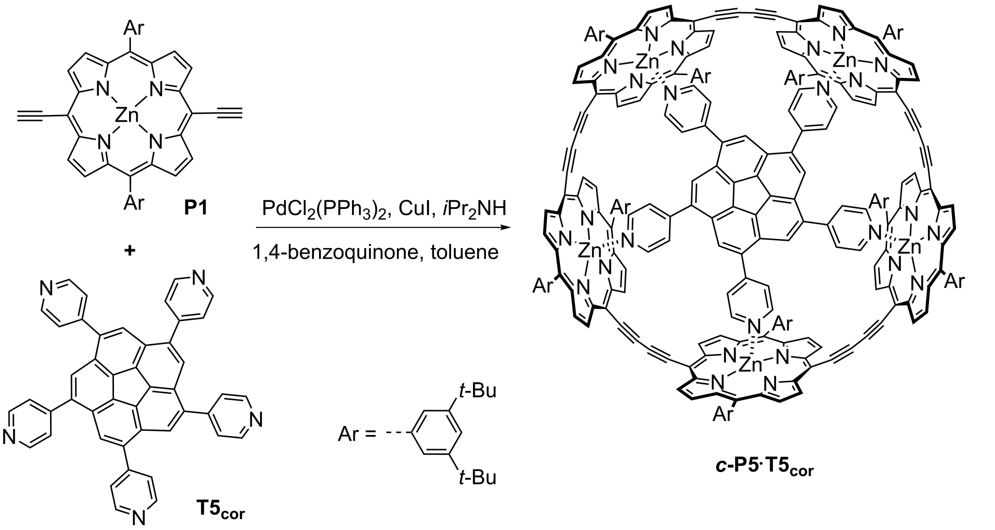

Scheme S7. The synthesis of ***c*-P5·T5$_{cor}$**

P1 (20 mg, 25 μmol) and **T5$_{cor}$** (3.1 mg, 5.0 μmol) were dissolved in toluene (500 mL). DIPA (5.0 mL) was added and the solution was stirred for 3 h. $PdCl_2(PPh_3)_2$ (45 mg, 0.064 mmol), CuI (48 mg, 0.25 mmol) and 1,4-benzoquinone (58 mg, 0.53 mmol) were added into the solution and the mixture was stirred at room temperature overnight. The mixture was filtered through basic Al_2O_3 and the solvent was evaporated. The residue was separated through size-exclusion column with pure toluene and the second band was collected. The first band was larger porphyrin nanorings and linear oligomers. The solvent was removed under vacuum and the residue was precipitated with DCM/MeOH to give the product as black solid (1.2 mg, 6.1%). ***c*-P5·T5$_{cor}$**: ^{1}H NMR (500 MHz, CD_2Cl_2): 9.60 (d, *J* = 4.5 Hz,10 H), 9.56 (d, *J* = 4.5 Hz, 10H), 8.75 (d, *J* = 4.5 Hz, 10H), 8.72 (d, *J* = 4.5 Hz, 10H), 7.97 (s, 10H), 7.74 (s, 10H), 7.64 (s, 10H), 5.44 (s, 5H), 4.97 (d, *J* = 6.5 Hz, 10H), 2.35 (d, *J* = 6.5 Hz, 10H) 1.54 – 1.36 (m, 180H); MALDI-TOF MS+: *m/z* 4608 ($[M]^+$, $C_{305}H_{275}N_{25}Zn_5$ requires: 4617), 3977 ([M-**T5$_{cor}$**]$^+$, $C_{260}H_{250}N_{20}Zn_5$ requires: 3982); UV-vis-NIR (toluene): λ_{max} (ε) 428 (2.8 × 10^5), 493 (4.3 × 10^5), 752 (2.3 × 10^5), 789 (2.2 × 10^5), 832 (1.9 × 10^5).

***c*-P5$_{OOct}$·T5$_{cor}$**

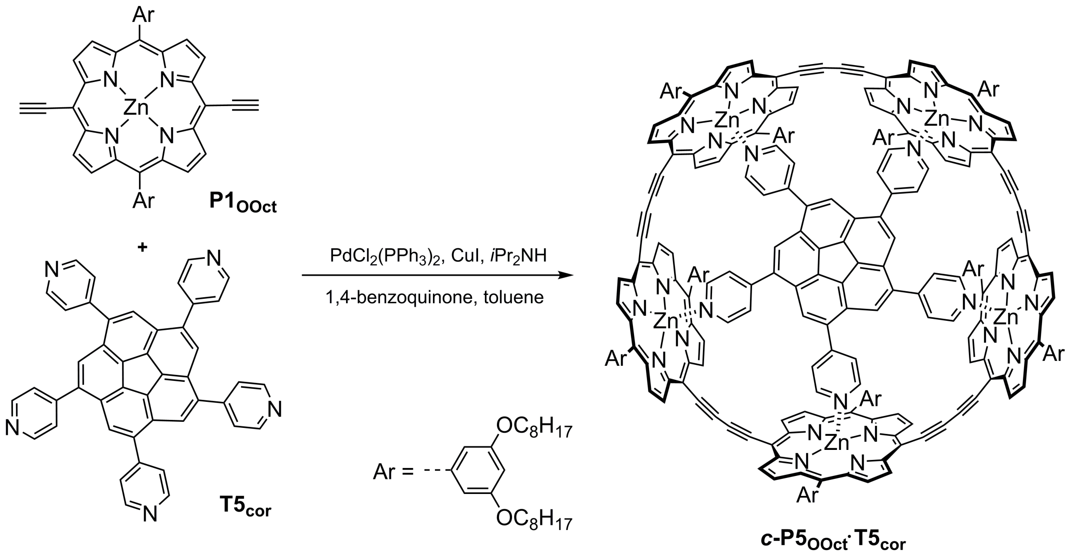

Scheme S8. The synthesis of ***c*-P5$_{OOct}$·T5$_{cor}$**

P1$_{OOct}$ (21 mg, 19 μmol) and **T5$_{cor}$** (2.5 mg, 4.0 μmol) were dissolved in toluene (400 mL). DIPA (4.0 mL) was added and the solution was stirred for 3 h. $PdCl_2(PPh_3)_2$ (35 mg, 0.050 mmol), CuI (38 mg, 0.19 mmol) and 1,4-benzoquinone (45 mg, 0.41 mmol) were added into the solution and the mixture was stirred at room temperature overnight. The mixture was filtered through basic Al_2O_3 and the solvent was evaporated. The residue was separated through size-exclusion column with pure toluene and the second band was collected. The first band was larger porphyrin nanorings and linear oligomers. The residue was separated using recycling GPC (toluene/1% pyridine, *v/v*) to give the desired product as black solid (0.50 mg, 2.0%). ***c*-P5$_{OOct}$·T5$_{cor}$**: ^{1}H NMR (500 MHz, CD_2Cl_2): 9.57 (d, J = 4.5 Hz, 10H), 9.52 (d, J = 4.5 Hz, 10H), 8.79 (d, J = 4.6 Hz, 10H), 8.76 (d, J = 4.6Hz, 10H), 7.15 (s, 10H), 6.95 (s, 10H), 6.75 (s, 10H), 5.44 (s, 5H), 4.96 (d, J = 6.5 Hz, 10H), 4.06 (t, J = 6.2 Hz, 20H), 3.88 (t, J = 6.2 Hz, 20H), 2.35 (d, J = 6.5 Hz, 10H), 1.84 – 0.70 (m, 300H); MALDI-TOF MS+: *m/z* 6060 ($[M]^+$, $C_{385}H_{435}N_{25}O_{20}Zn_5$ requires: 6059), 5420 ([M-**T5$_{cor}$**]$^+$, $C_{340}H_{410}N_{20}O_{20}Zn_5$ requires: 5424); UV-vis-NIR (toluene): λ_{max} (ε) 428 (2.3×10^5), 494 (4.2×10^5), 746 (2.2×10^5), 783 (2.2×10^5), 825 (2.0×10^5).

C. Spectra Confirming Identity and Purity of New Compounds

C1. Full Spectra

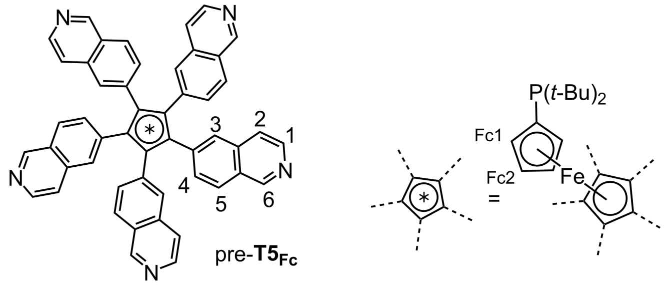

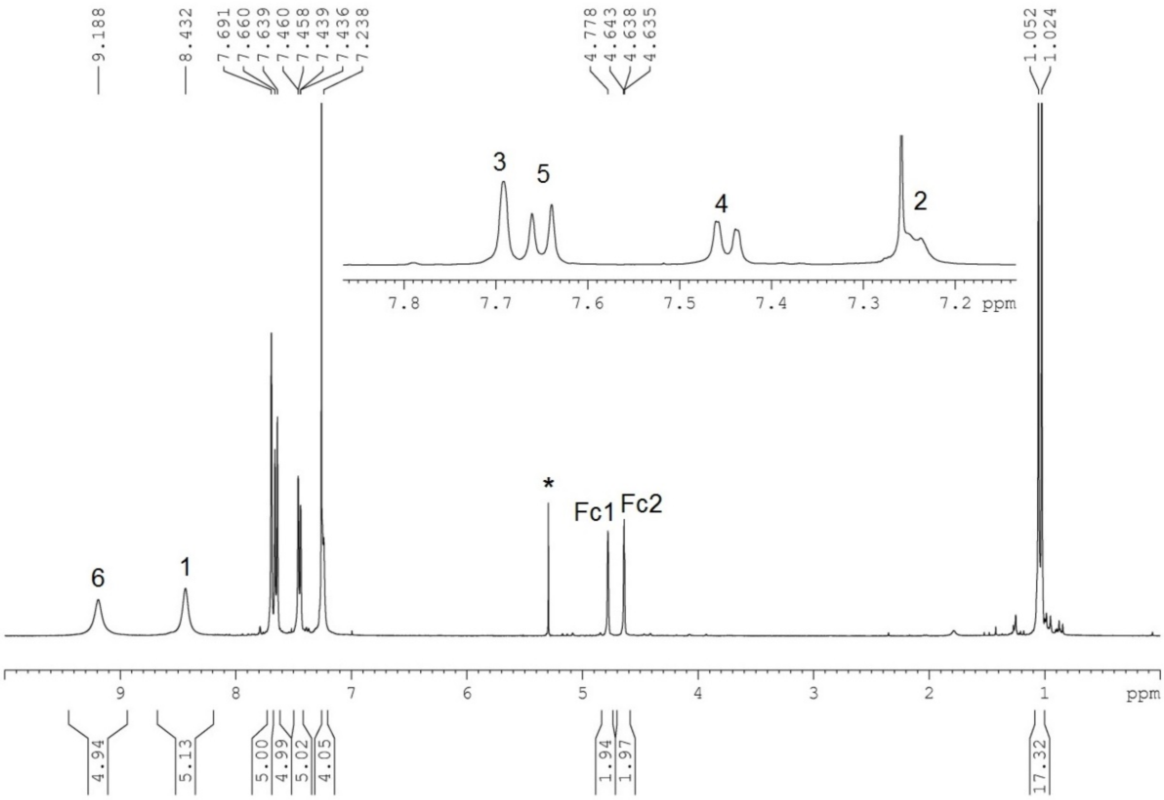

Figure S1. ^{1}H NMR spectrum of pre-**T5$_{Fc}$** ($CDCl_3$, 400 MHz, 298 K, * indicates a trace of CH_2Cl_2 impurity).

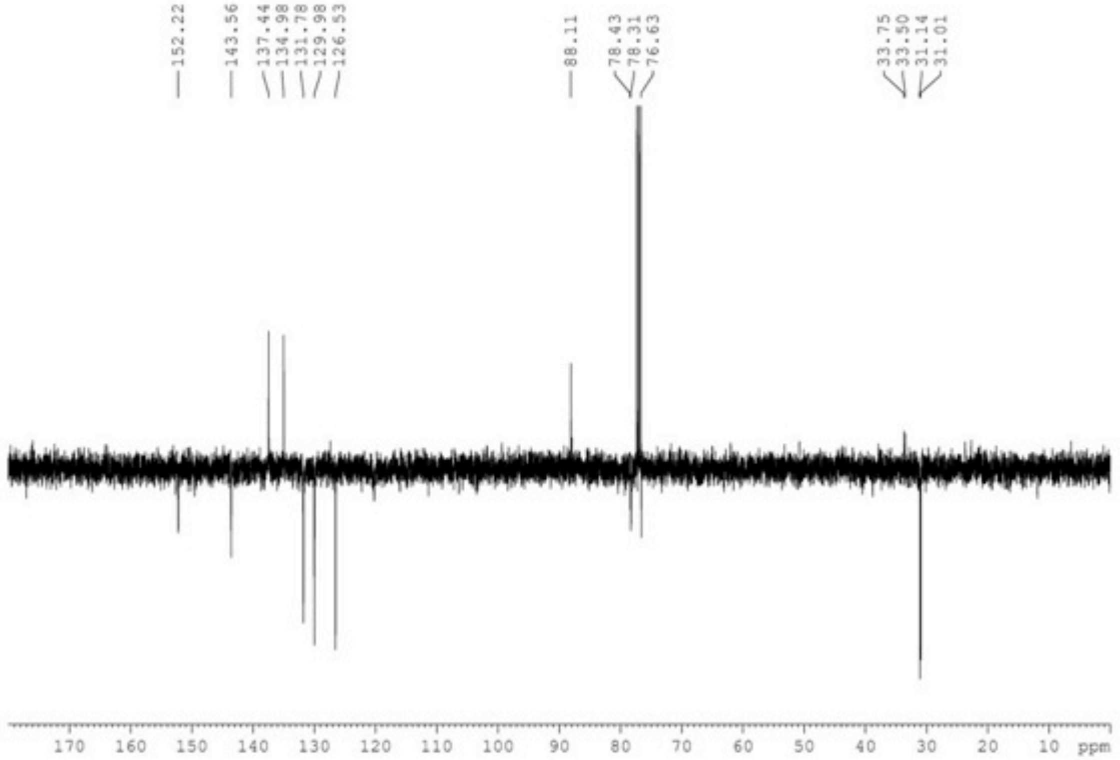

Figure S2. ^{13}C NMR spectrum (DEPTQ) of pre-$\mathbf{T5_{Fc}}$ ($CDCl_3$, 100 MHz, 298 K).

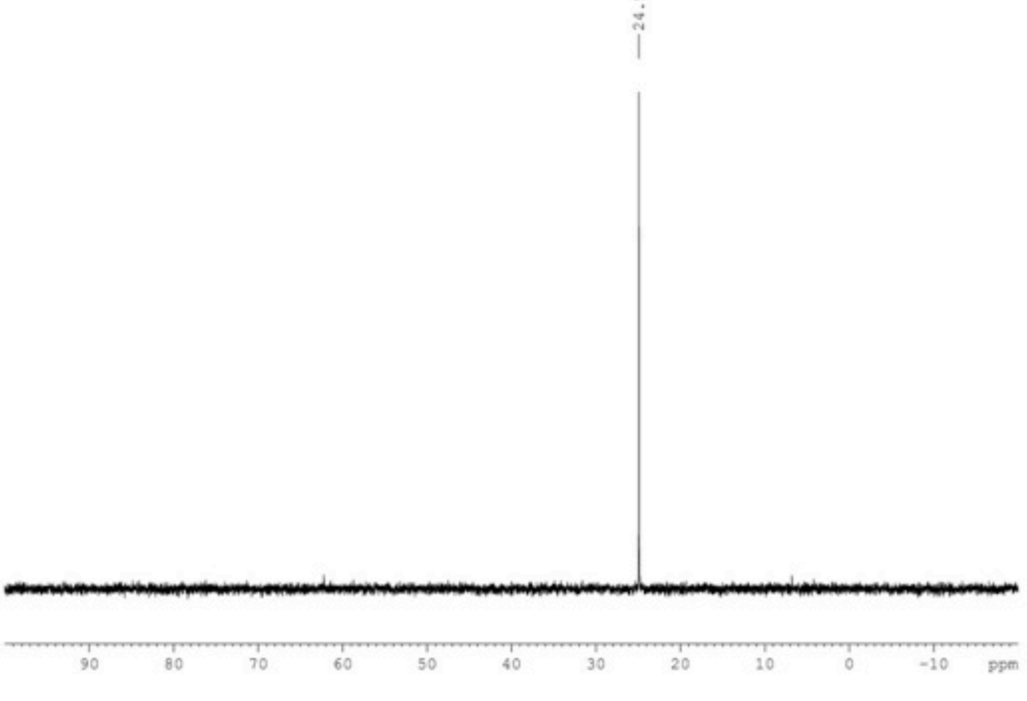

Figure S3. ^{31}P NMR spectrum with ^{1}H decoupling of pre-$\mathbf{T5_{Fc}}$ ($CDCl_3$, 162 MHz, with ^{1}H-NMR decoupling, 298 K).

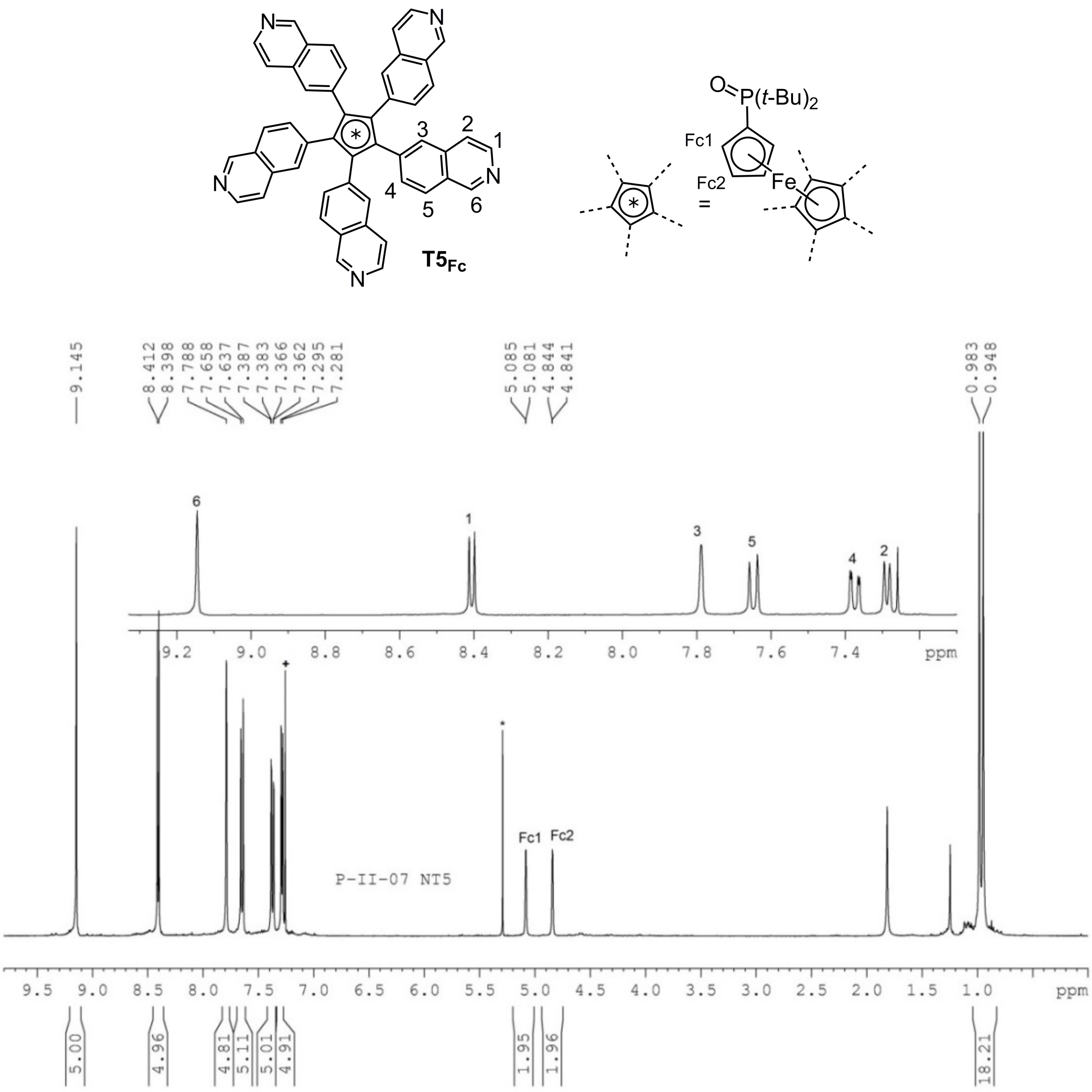

Figure S4. ^{1}H NMR spectrum of **T5$_{Fc}$** ($CDCl_3$, 400 MHz, 298 K, * indicates a trace of CH_2Cl_2 impurity).

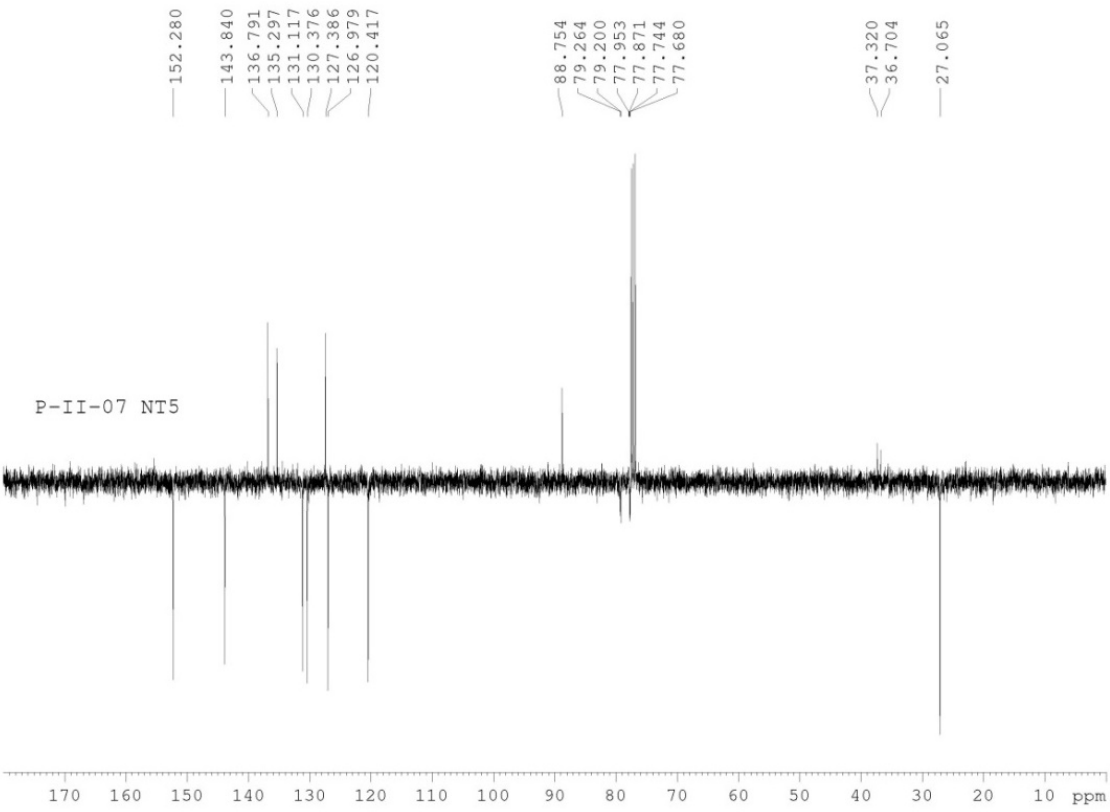

Figure S5. ^{13}C NMR spectrum (DEPTQ) of **T5$_{Fc}$** ($CDCl_3$, 100 MHz, 298 K).

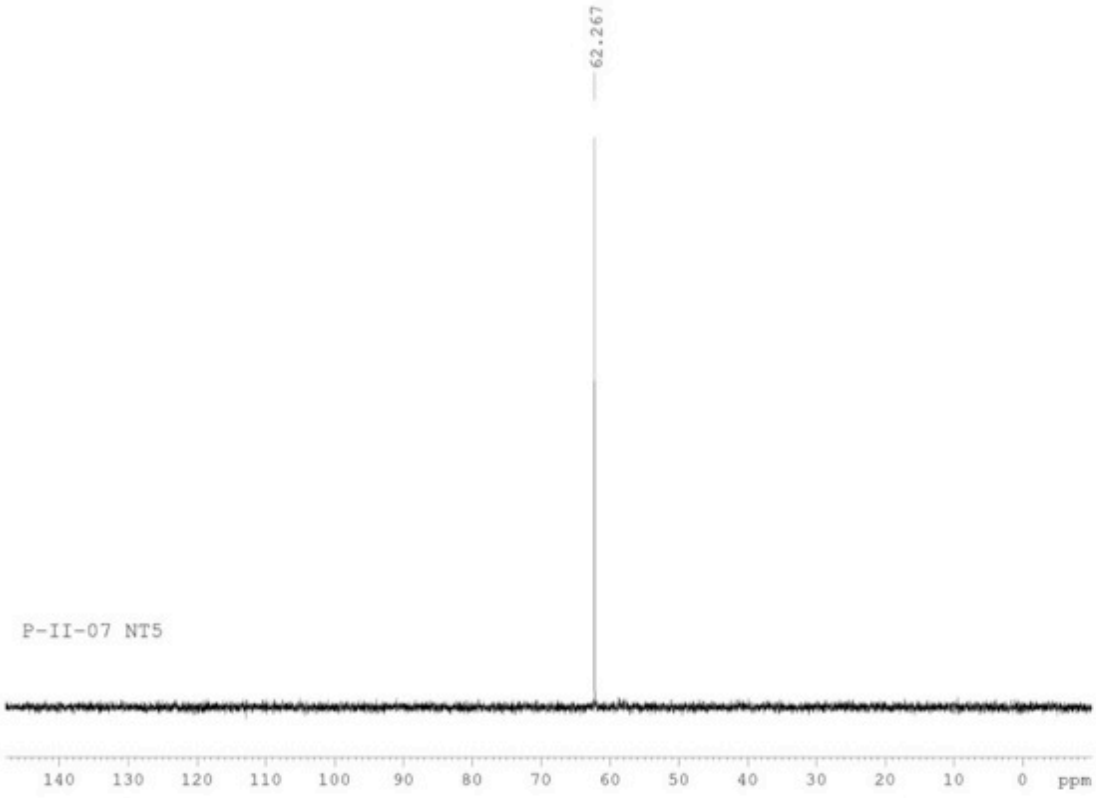

Figure S6. ^{31}P NMR spectrum with ^{1}H decoupling of **T5$_{Fc}$** ($CDCl_3$, 162 MHz, 298 K).

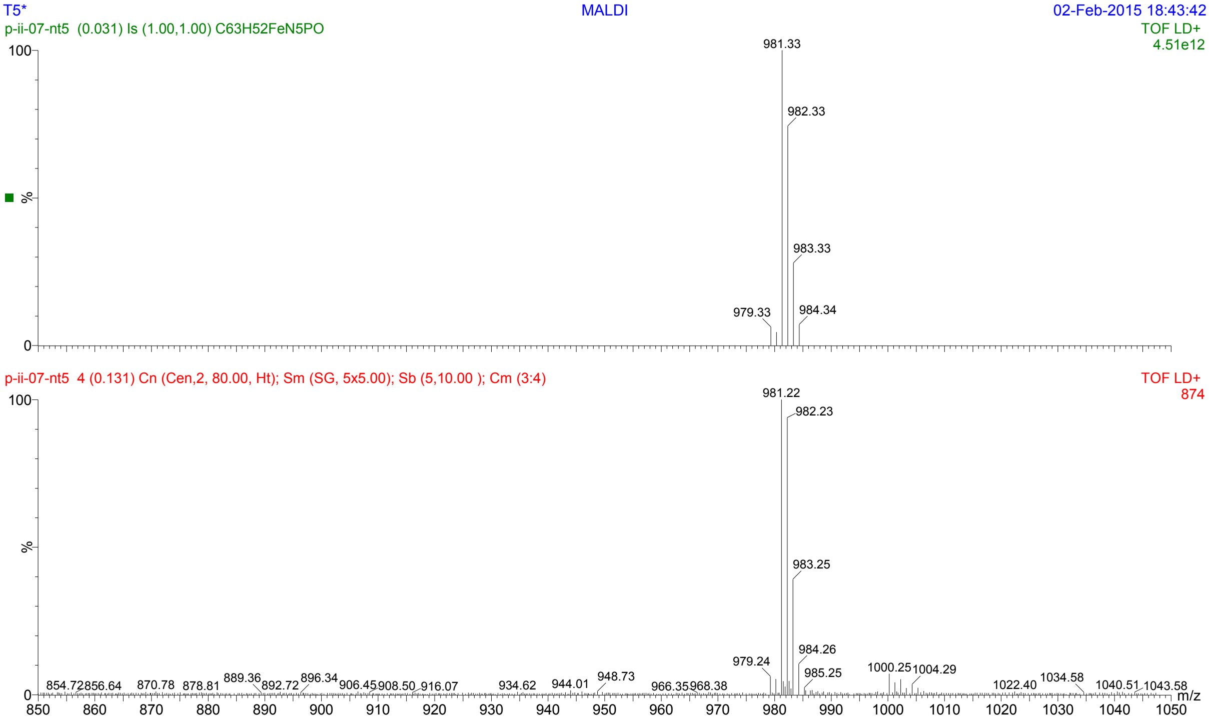

Figure S7. MALDI-MS spectrum of **T5$_{Fc}$** (matrix: DCTB, $C_{63}H_{52}FeN_5PO$ requires: 981.33, found: 981.22).

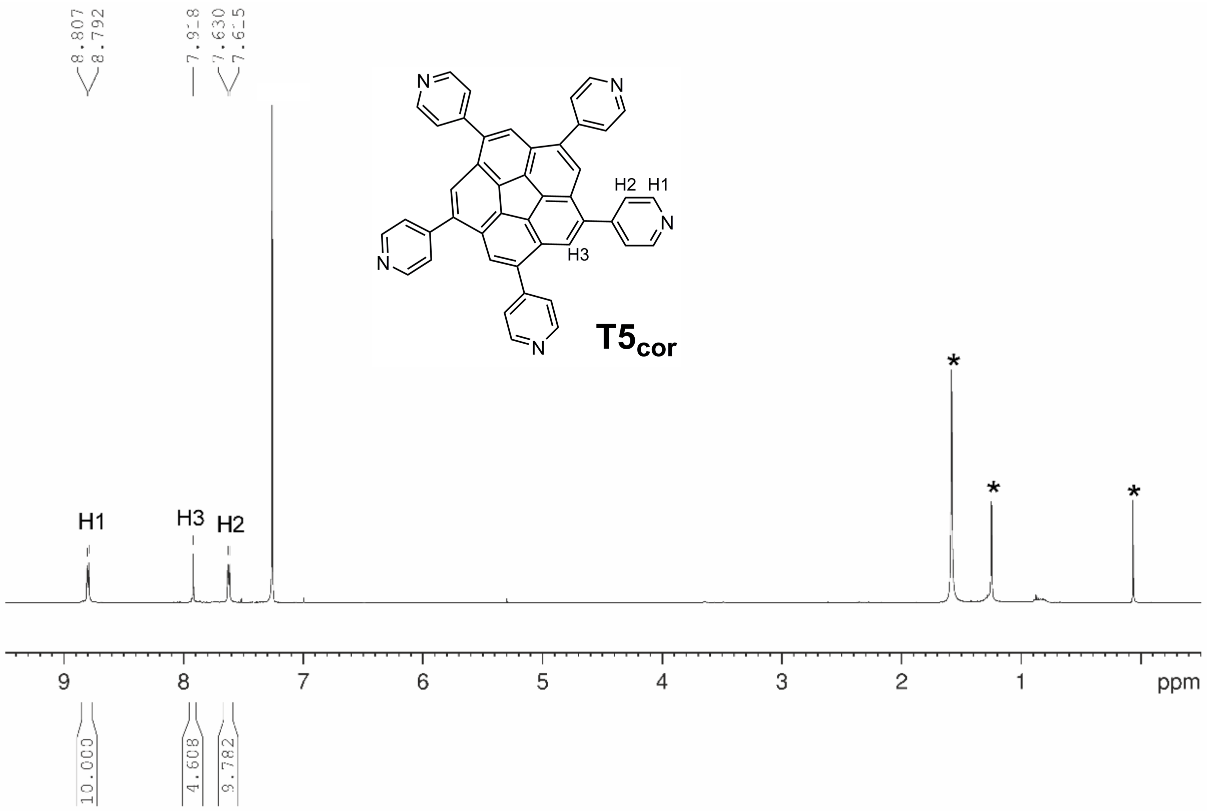

Figure S8. ^{1}H NMR spectrum of **T5$_{cor}$** ($CDCl_3$, 400 MHz, 298 K, * represents residual solvent or impurity).

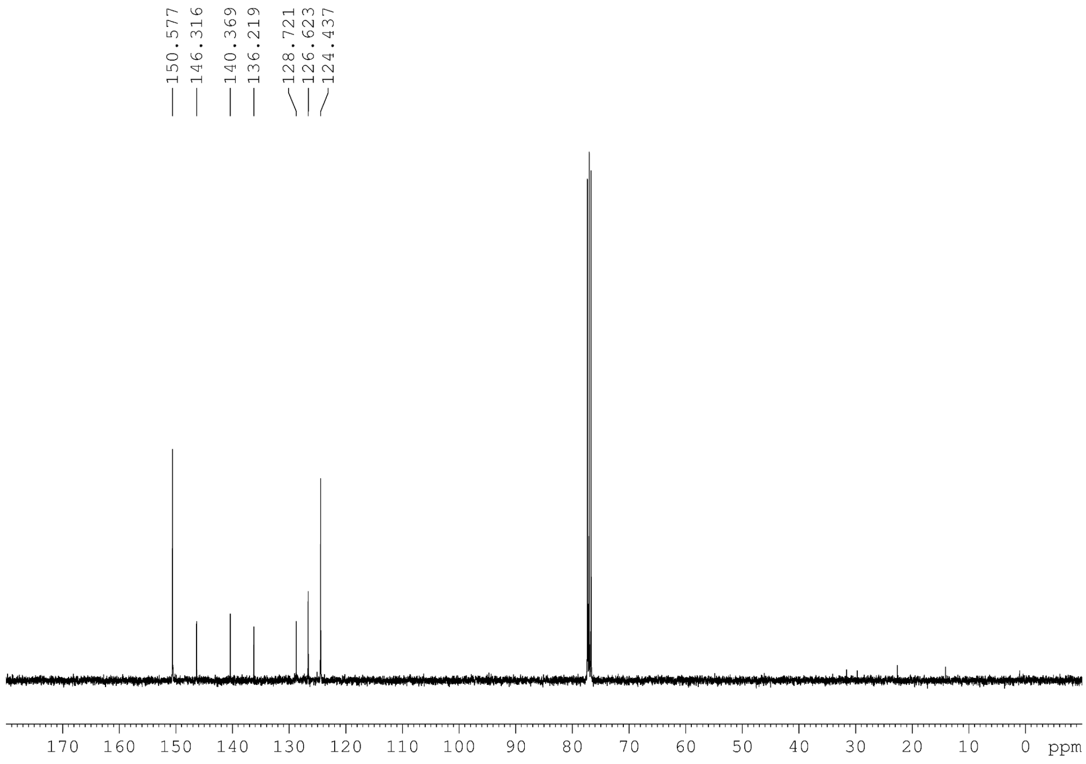

Figure S9. ^{13}C NMR spectrum of **T5$_{cor}$** ($CDCl_3$, 400 MHz, 298 K)

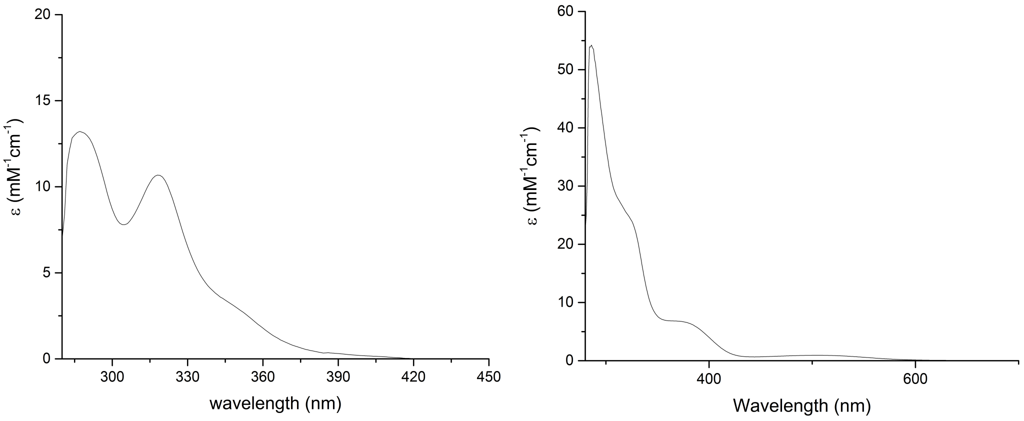

Figure S10. UV-vis spectra of **T5$_{Fc}$** (left) and **T5$_{cor}$** (right) in toluene.

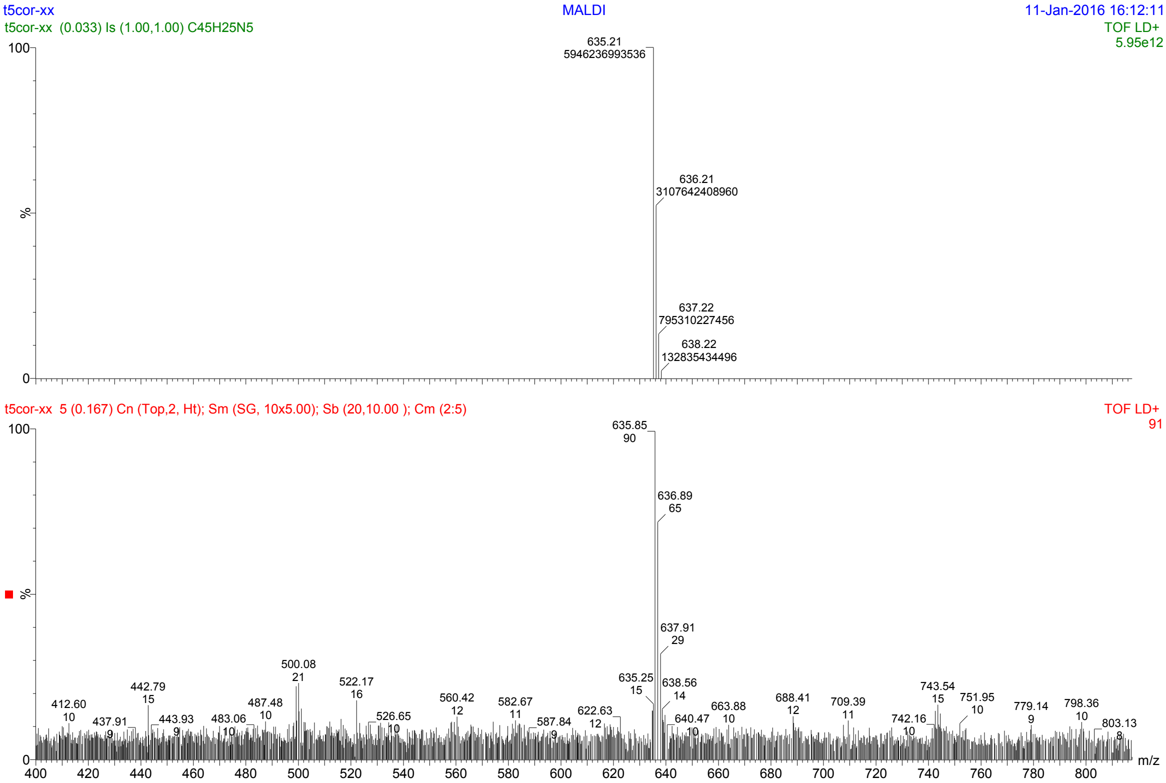

Figure S11. MALDI-MS spectrum of **T5$_{cor}$** (matrix: DCTB, $C_{45}H_{25}N_5$ requires: 635.21, found: 635.85)

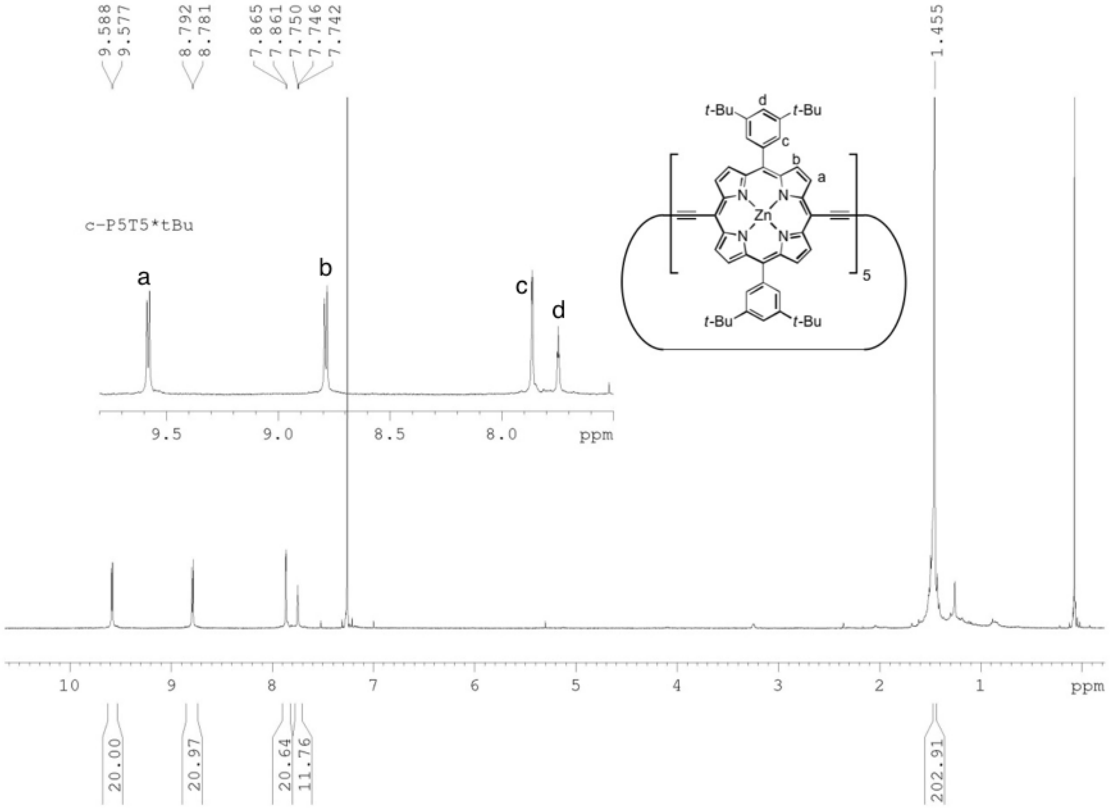

Figure S12. ^{1}H NMR spectrum of ***c*-P5** ($CDCl_3$, 400 MHz, 298 K).

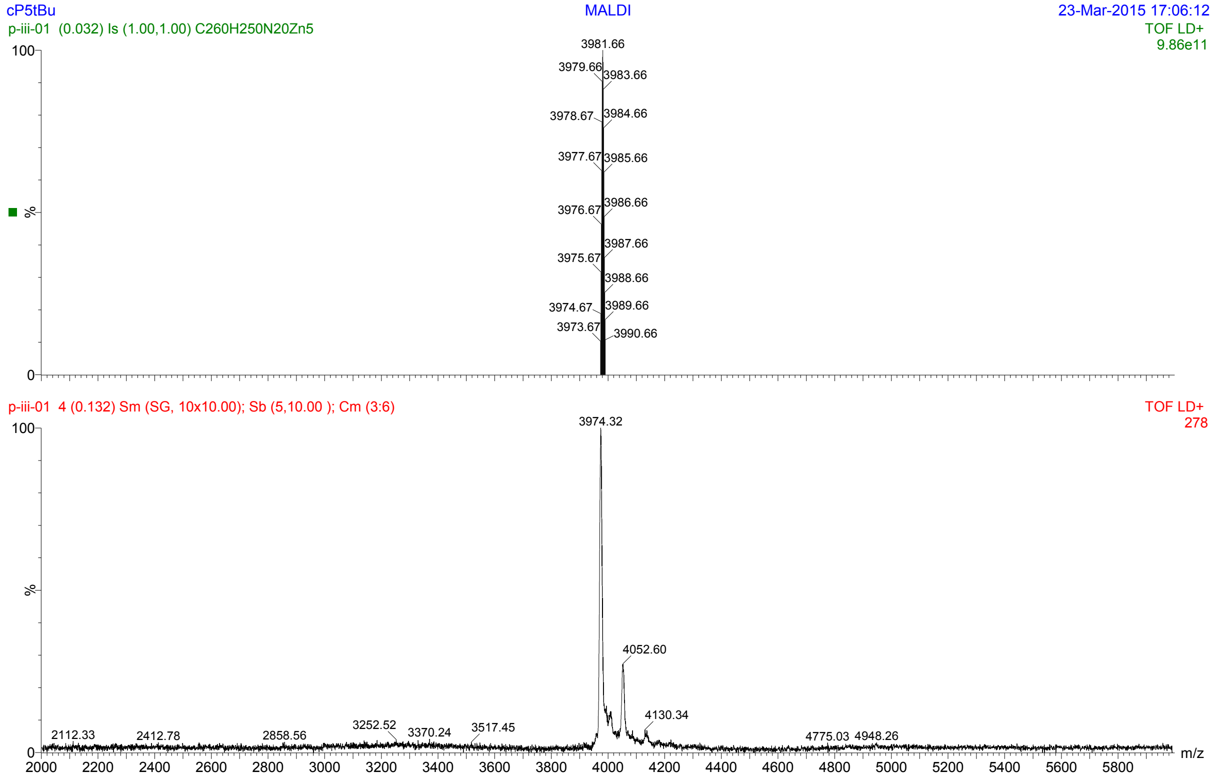

Figure S13. MALDI-MS spectrum of ***c*-P5** (matrix: DCTB, $C_{260}H_{250}N_{20}Zn_5$ requires: 3982, found: 3974).

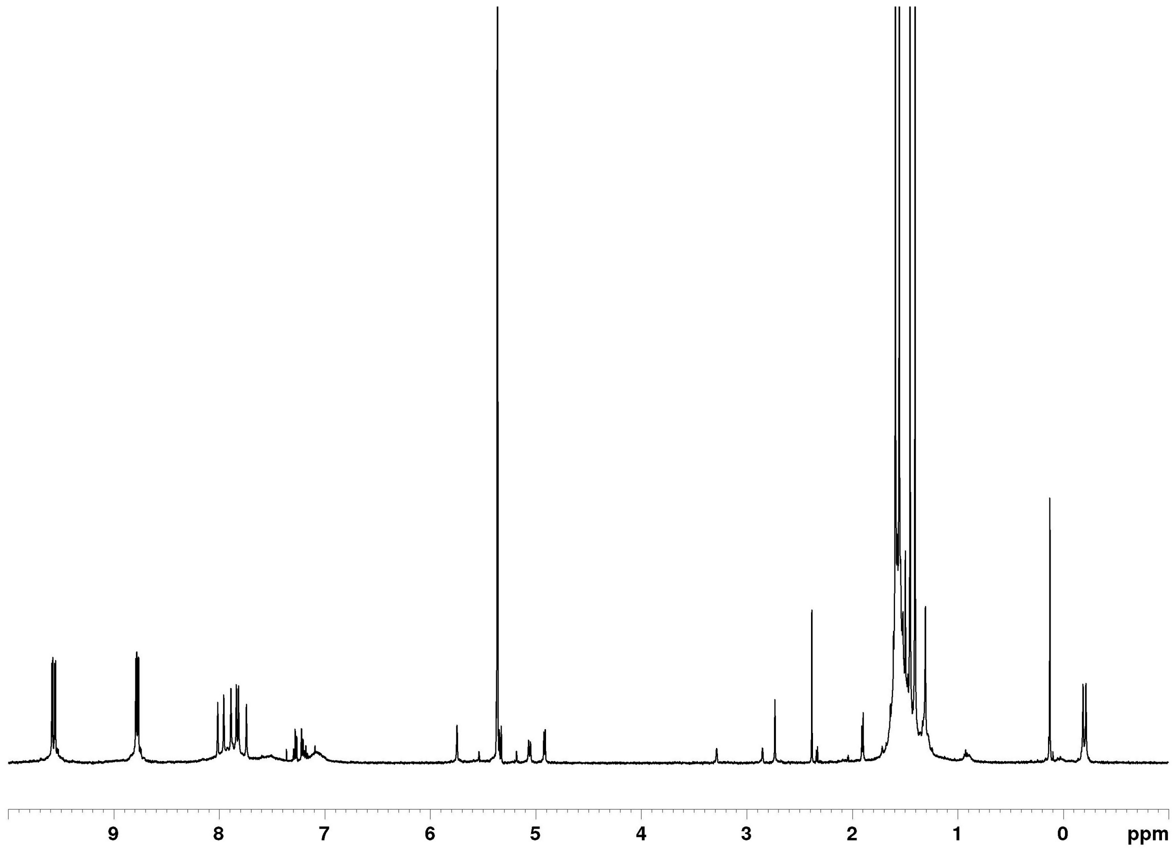

Figure S14. The original ^{1}H NMR spectrum of ***c*-P5·T5$_{Fc}$** (CD_2Cl_2, 500 MHz, 298 K).

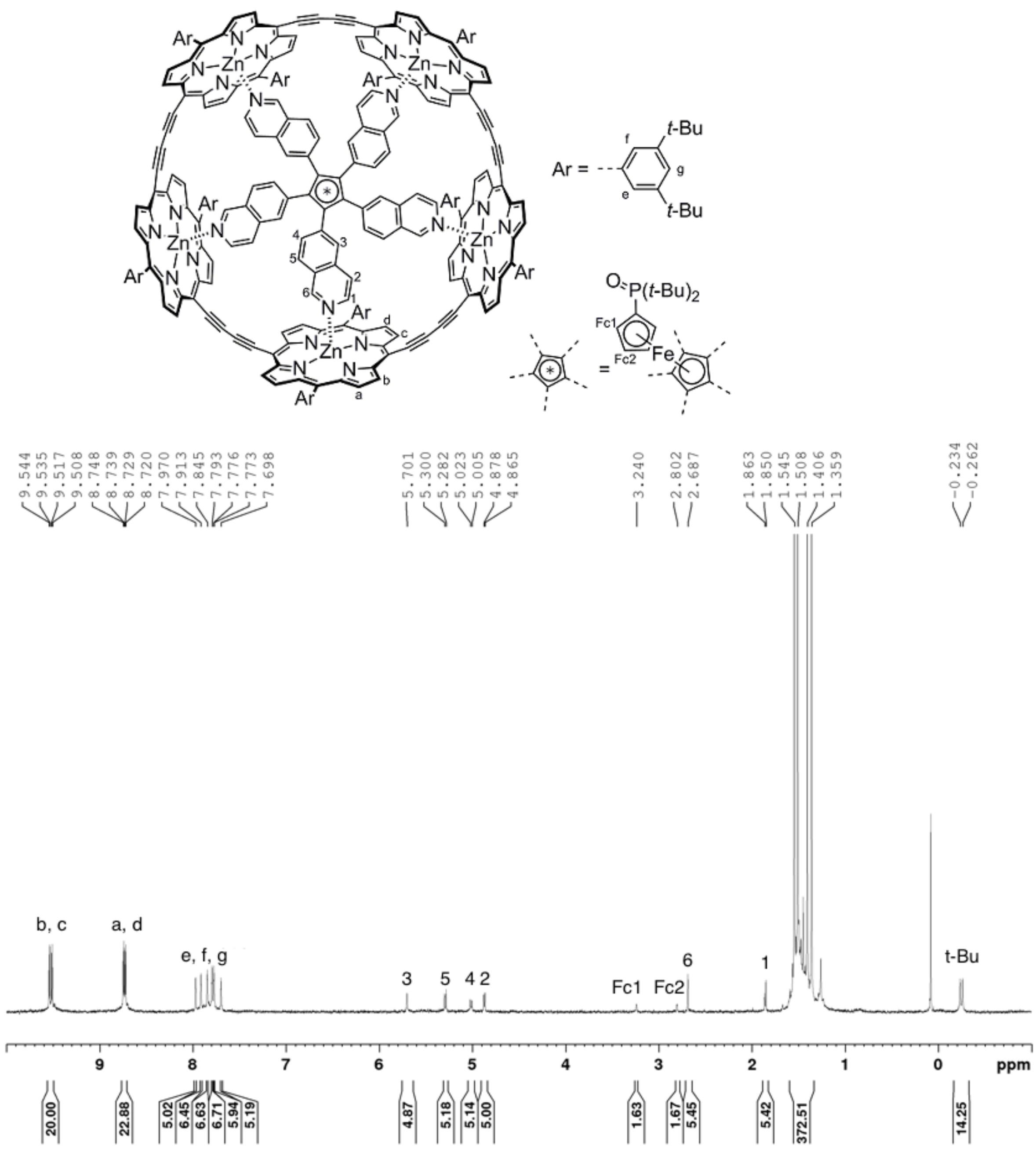

Figure S15. ^{1}H NMR spectrum of ***c*-P5·T5$_{\mathbf{Fc}}$** (diffusion edited gradient strength 40%, CD_2Cl_2, 500 MHz, 298 K).

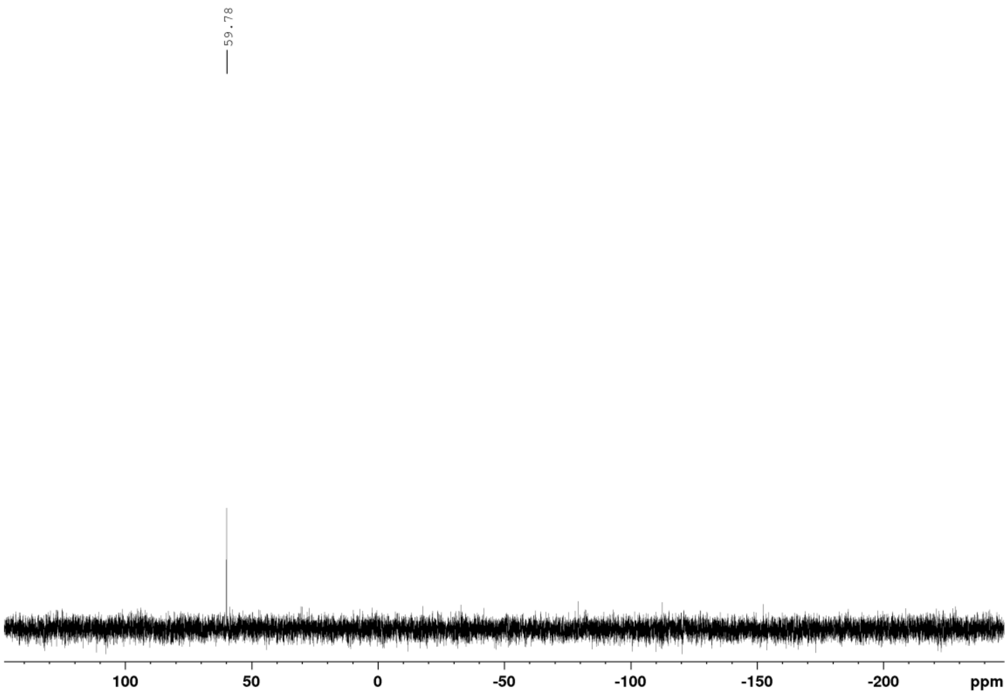

Figure S16. ^{31}P NMR spectrum with ^{1}H decoupling of ***c*-P5·T5$_{Fc}$** (CD_2Cl_2, 162 MHz, 298 K).

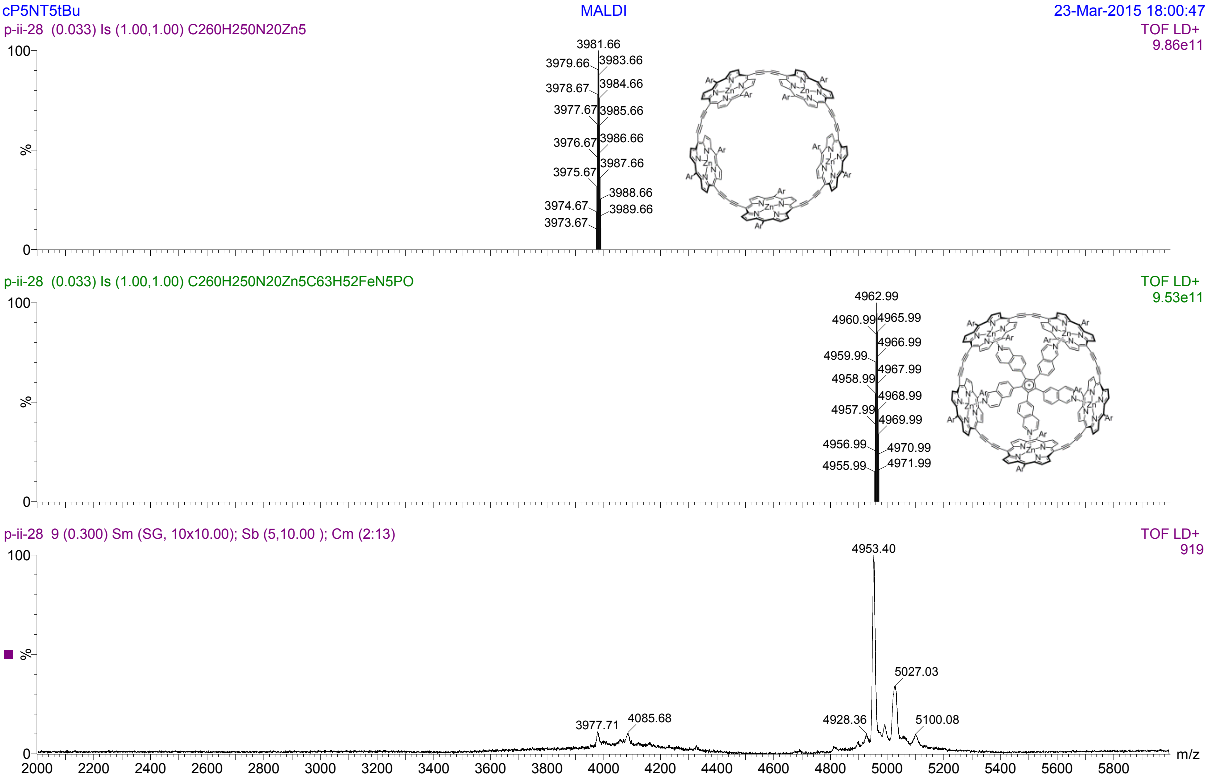

Figure S17. MALDI-MS spectrum of ***c*-P5·T5$_{Fc}$** (matrix: DCTB, $C_{323}H_{302}FeN_{25}Zn_5PO$ requires: 4963, found: 4953; $C_{260}H_{250}N_{20}Zn_5$ ([M-**T5$_{Fc}$**]$^{+}$) requires: 3982, found: 3978).

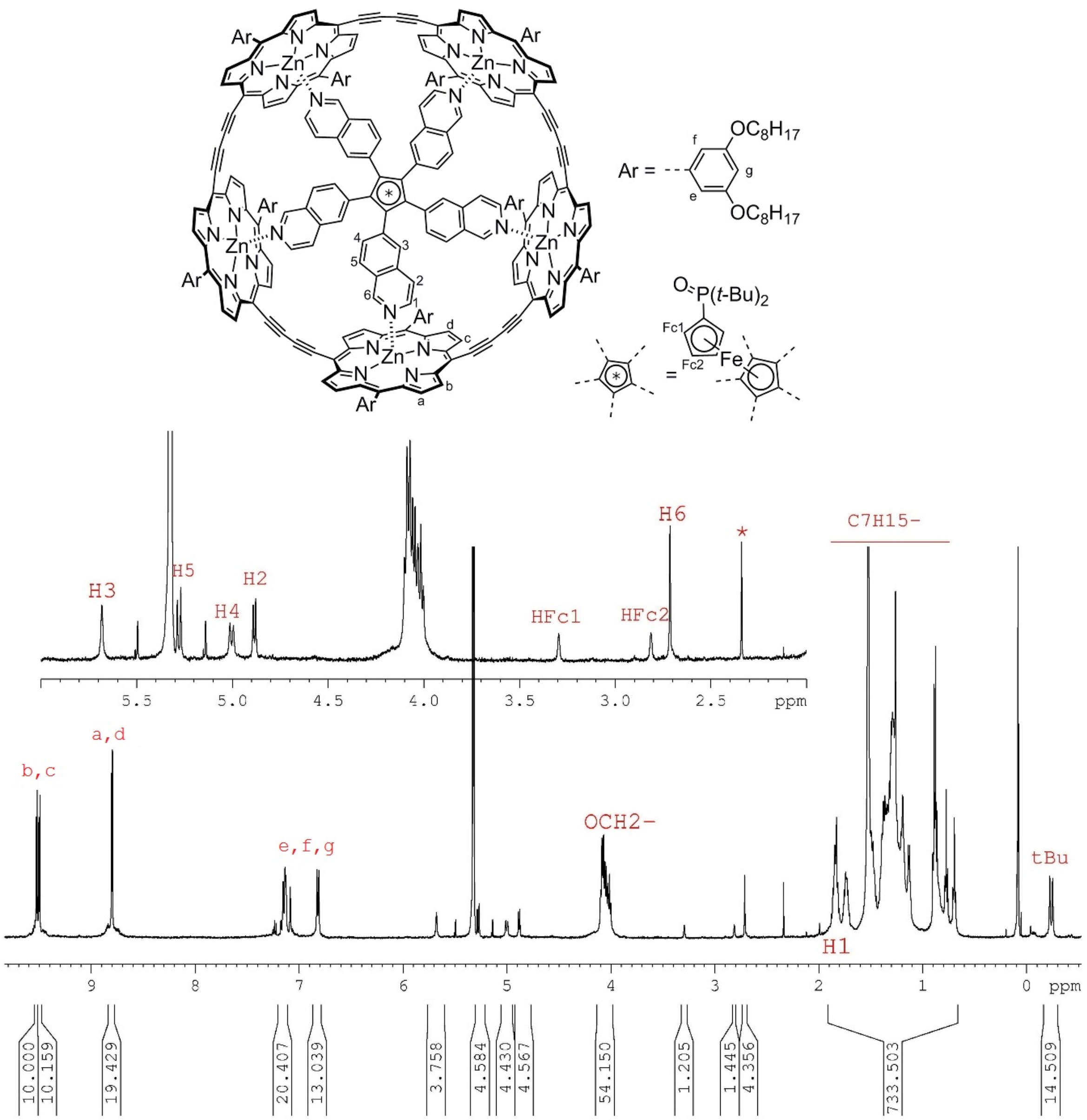

Figure S18. ^{1}H-NMR spectrum of ***c*-P5$_{OOct}$·T5$_{Fc}$** (CD_2Cl_2, 500 MHz, 298 K, ***** represents residual solvent or impurity).

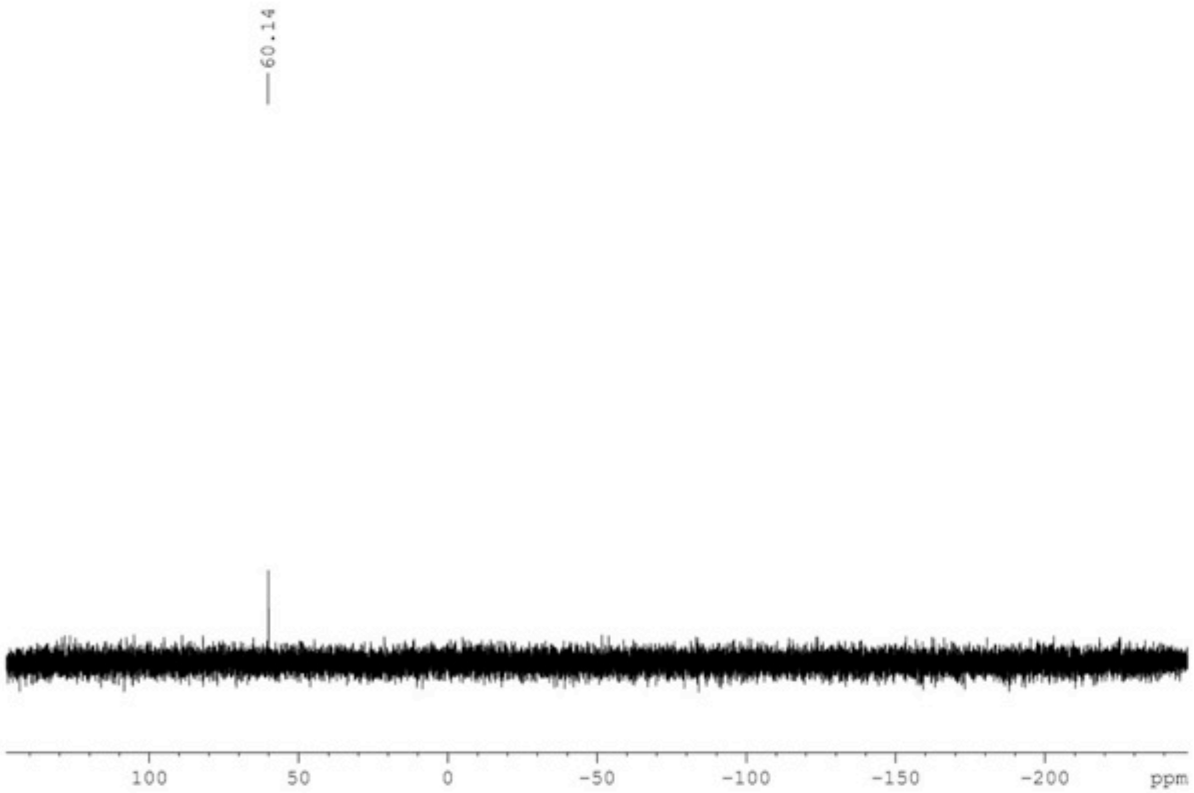

Figure S19. ^{31}P-NMR spectrum with ^{1}H decoupling of ***c*-P5**$_{\mathbf{OOct}}$**·T5**$_{\mathbf{Fc}}$ (CD_2Cl_2, 162 MHz, 298 K).

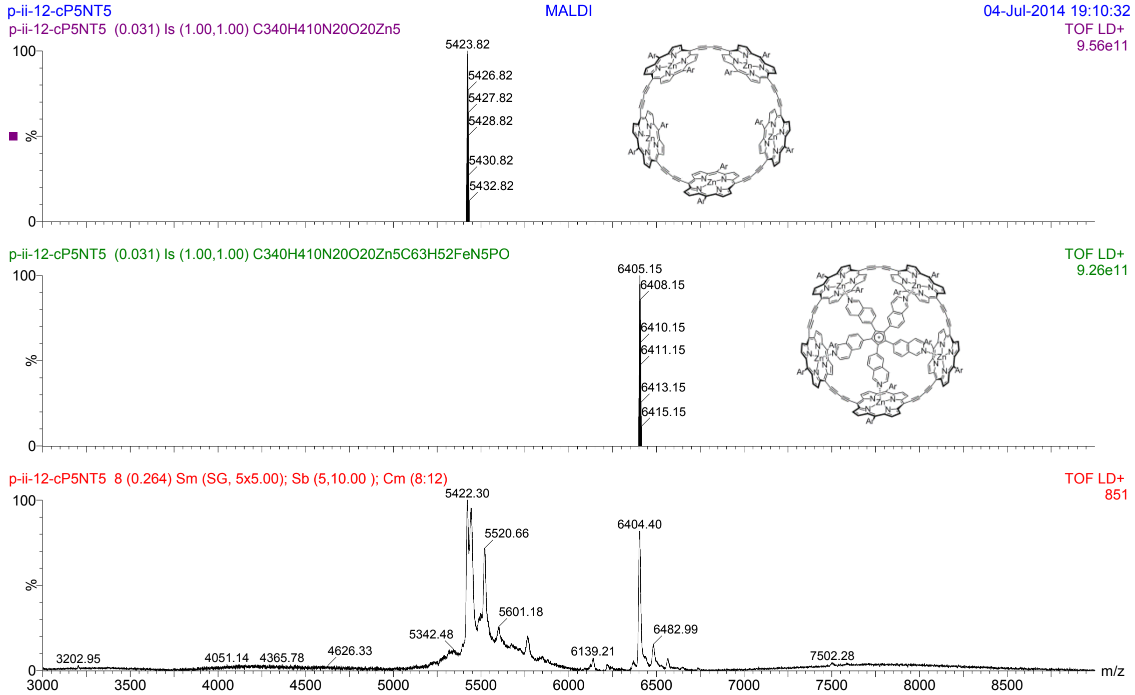

Figure S20. MALDI-MS spectrum of ***c*-P5**$_{\mathbf{OOct}}$**·T5**$_{\mathbf{Fc}}$ (matrix: DCTB, $C_{403}H_{462}FeN_{25}Zn_5PO_{21}$ requires: 6405, found: 6404; $C_{340}H_{410}N_{20}O_{20}Zn_5$ ([M-**T5**$_{\mathbf{Fc}}$]$^+$) requires: 5424, found: 5422).

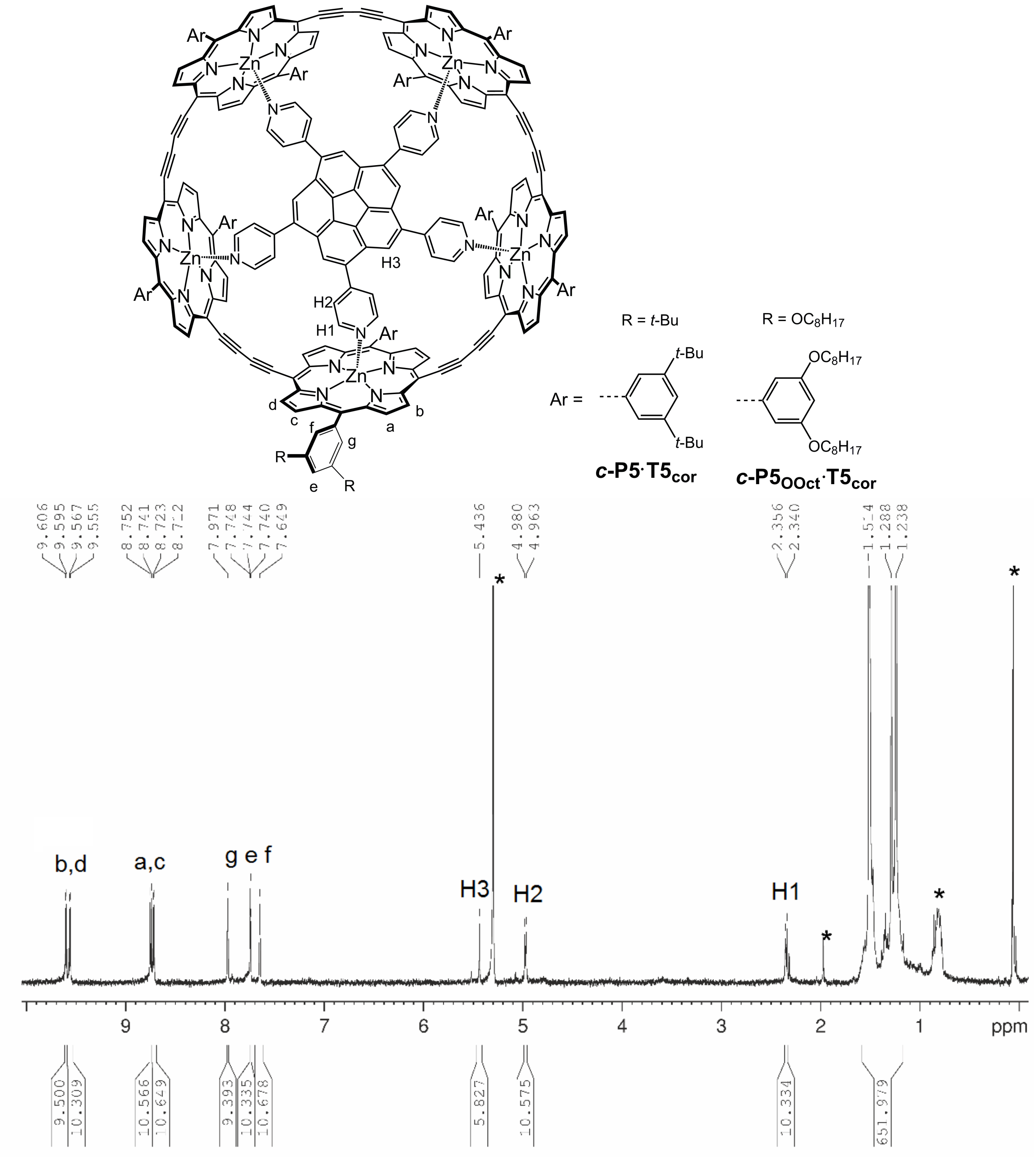

Figure S21. ^{1}H-NMR spectrum of ***c*-P5·T5$_{cor}$** (CD_2Cl_2, 500 MHz, 298 K, * represents residual solvent or impurity).

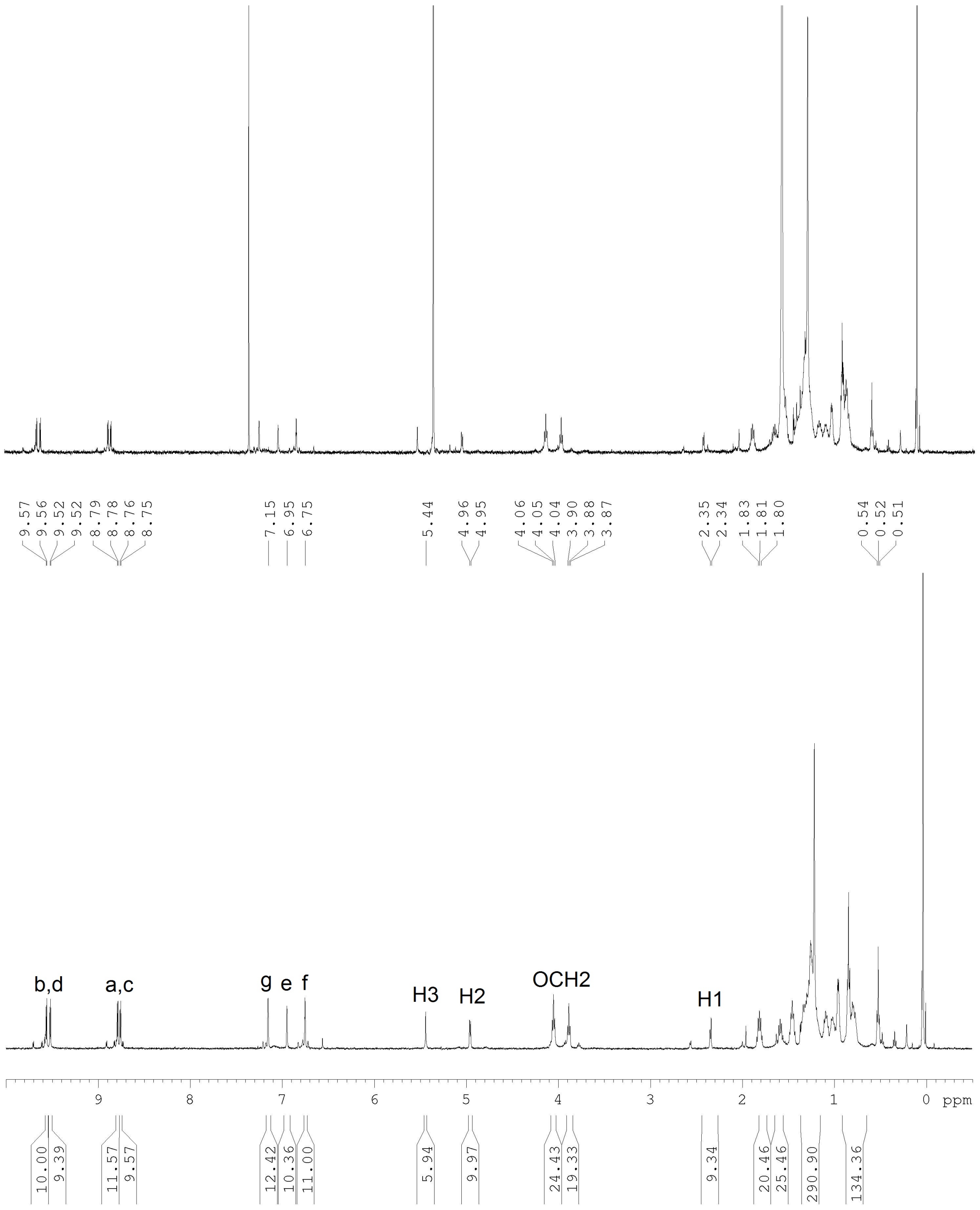

Figure S22. ^{1}H NMR spectra of ***c*-P5$_{OOct}$·T5$_{cor}$** (CD_2Cl_2, 500 MHz, 298 K, top: original spectrum; bottom: Diffusion-edited spectrum, gradient strength 40%).

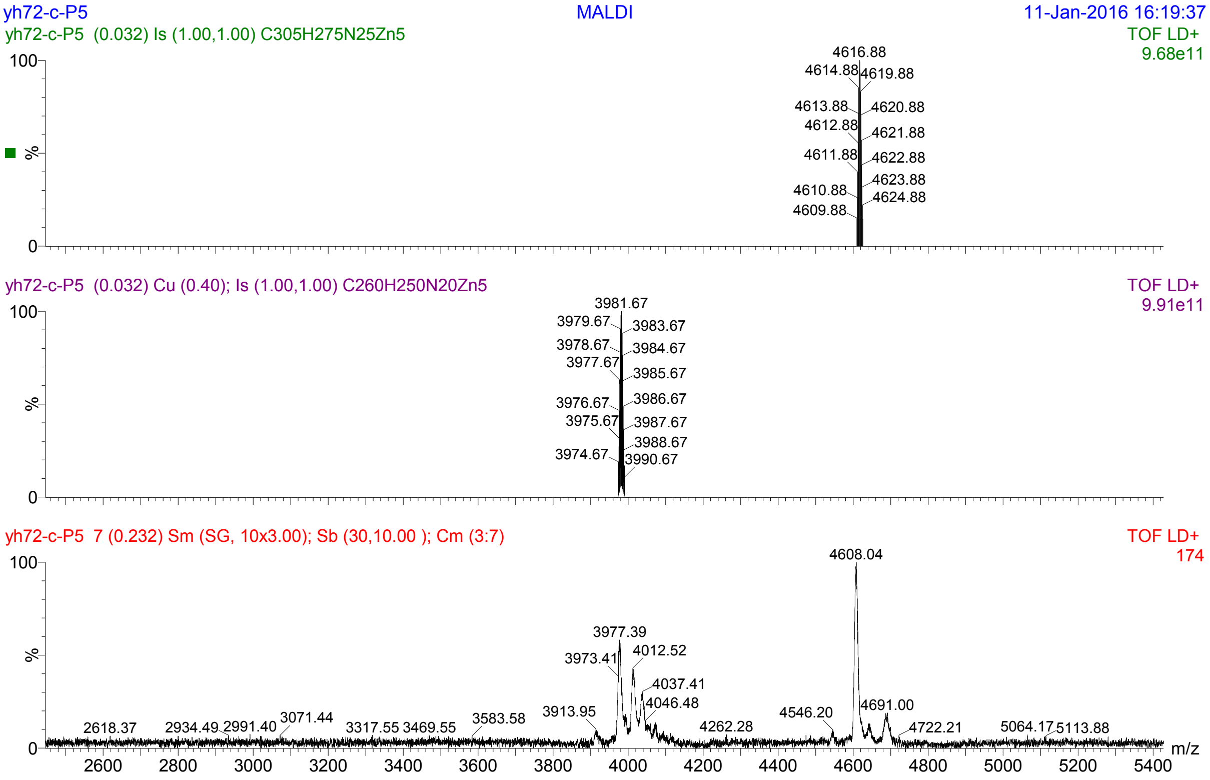

Figure S23. MALDI-MS spectrum of ***c*-P5·T5$_{\mathbf{cor}}$** (matrix: DCTB, $C_{305}H_{275}N_{25}Zn_5$ requires: 4617, found: 4608; $C_{260}H_{250}N_{20}Zn_5$ ([M-**T5$_{\mathbf{cor}}$**]$^+$) requires: 3982, found: 3977).

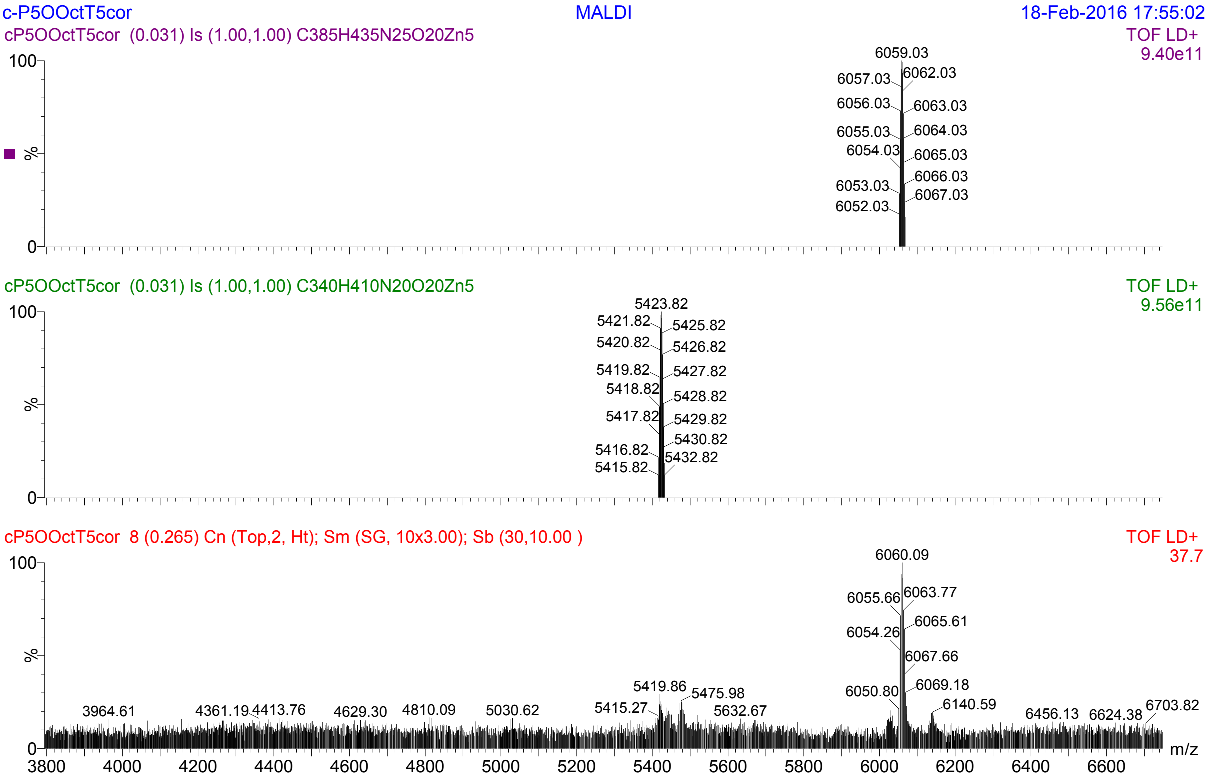

Figure S24. MALDI-MS spectrum of ***c*-P5$_{\mathbf{OOct}}$·T5$_{\mathbf{cor}}$** (matrix: DCTB, $C_{385}H_{435}N_{25}O_{20}Zn_5$ requires: 6059, found: 6060; $C_{340}H_{410}N_{20}O_{20}Zn_5$ ([M-**T5$_{\mathbf{cor}}$**]$^+$) requires: 5424, found: 5420).

*C2. Characterization of **c-P5·T5$_{Fc}$***

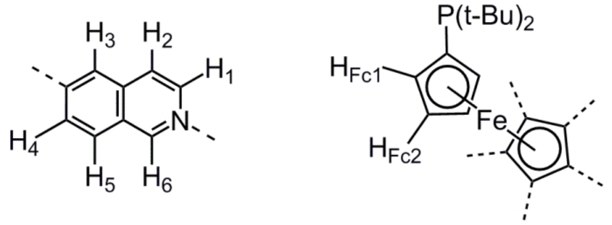

Figure S25. Designation of the protons in **T5$_{Fc}$**.

The chemical shifts of the protons on porphyrin structure ***c*-P5·T5$_{Fc}$** are similar to those reported for ***c*-P6·T6**.[S5] The major task is to assign the protons in the template: isoquinoline unit, cyclopentadiene unit and *tert*-butyl unit.

We can start the identification process from the HSQC spectrum. The correlation signals of the 6 protons on the isoquinoline structure are in the range between 120 ppm and 145 ppm, which is the range of aromatic carbons. H1 and H6 can be identified by the chemical shifts of the carbon atoms that they attach to; the assignment of other protons on the isoquinoline unit, HFc$_1$ and HFc$_2$ will be shown in following figures.

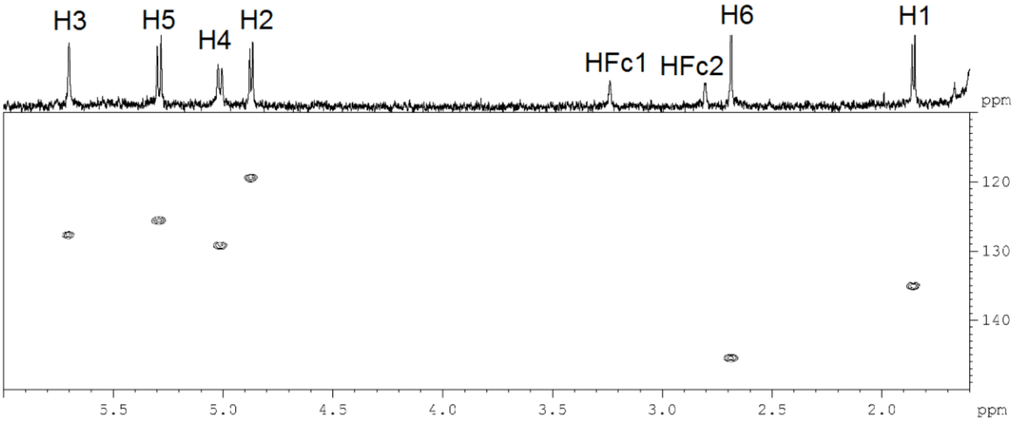

Figure S26. Identification of six isoquinoline protons from ^{1}H-^{13}C HSQC spectrum (diffusion-edited ^{1}H spectrum projection of ***c*-P5·T5$_{Fc}$**, CD_2Cl_2, 500 MHz, 298 K). HFc$_1$ and HFc$_2$ don't have correlation signals in this figure since the carbon atoms that they attach to have the chemical shifts in the range between 75 and 80 ppm.

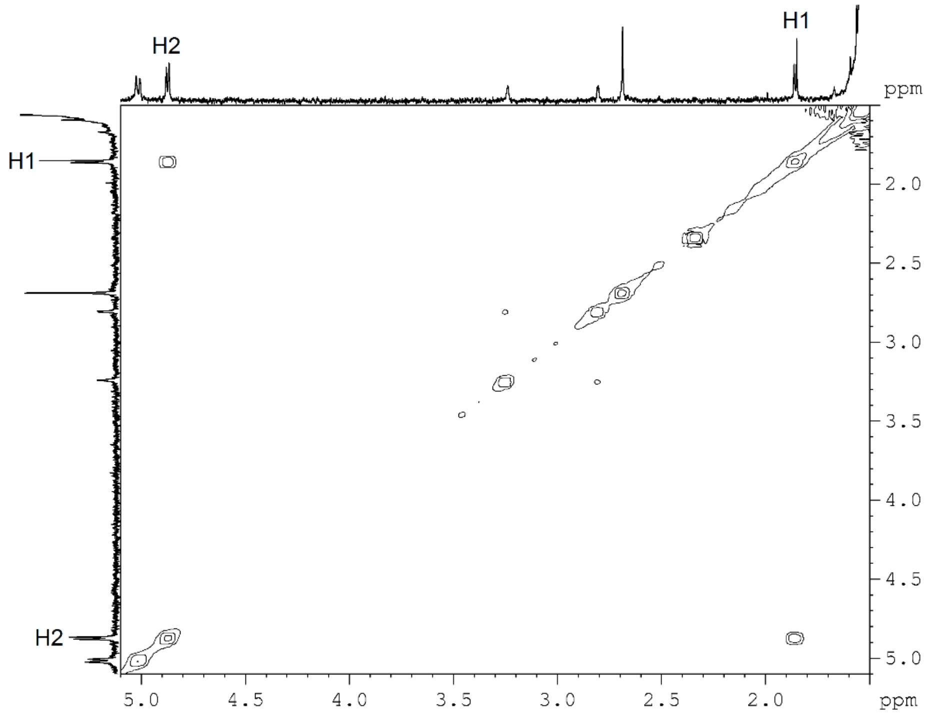

Figure S27. Correlation signal of H_1 - H_2 in ^1H-^1H COSY spectrum (diffusion-edited ^1H spectrum projection, CD_2Cl_2, 500 MHz, 298 K).

As is shown in **Figure S27**, assignment of the isoquinoline peaks starts from a COSY peak between H_1 (1.86 ppm) and H_2 (4.87 ppm). The peak of H_1 at 1.86 ppm is typical of the most shielded protons in the template.

As is shown in **Figure S28**, H_2 has strong NOE signals with H_1 and H_3 (5.70 ppm).

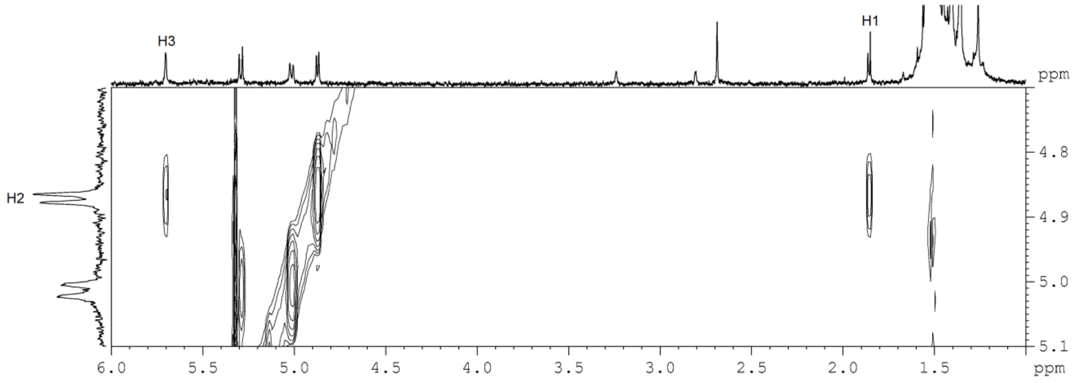

Figure S28. Correlation signals of H_1 - H_2 and H_3 - H_2 in ^1H-^1H NOESY spectrum (diffusion-edited ^1H spectrum projection of ***c*-P5·T5**$_{\mathbf{Fc}}$, CD_2Cl_2, 500 MHz, 298 K).

As is shown in **Figure S29**, H_3 has NOE signals with H_2 (strong) and H_4 (5.01 ppm, weak).

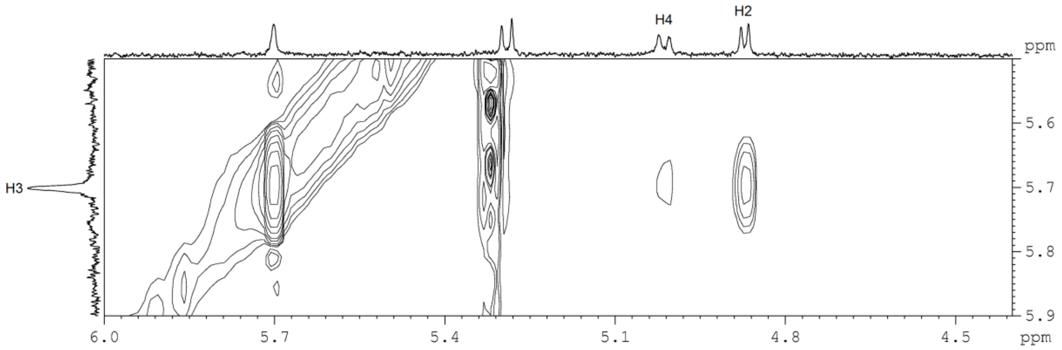

Figure S29. Correlation signals of H_3 - H_4 and H_3 - H_2 in ^{1}H-^{1}H NOESY spectrum (diffusion-edited ^{1}H spectrum projection of ***c*-P5·T5$_{Fc}$**, CD_2Cl_2, 500 MHz, 298 K).

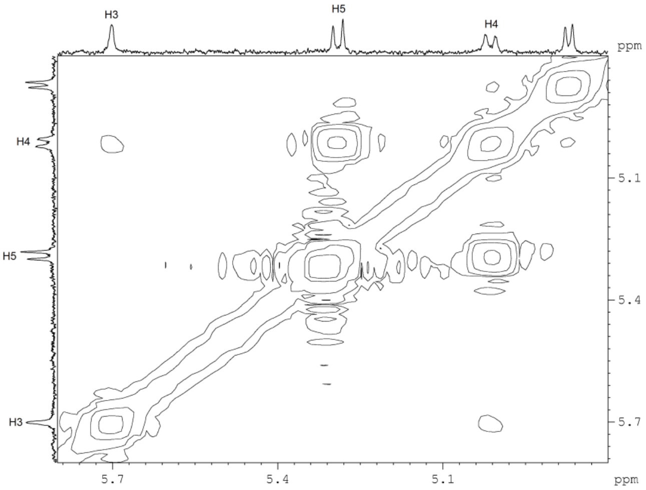

Figure S30. Correlation signal of H_4 - H_5 and H_4 - H_3 in ^{1}H-^{1}H COSY spectrum (diffusion-edited ^{1}H spectrum projection of ***c*-P5·T5$_{Fc}$**, CD_2Cl_2, 500 MHz, 298 K).

As is shown in **Figure S30**, H_4 has COSY signals with H_5 (5.30 ppm, strong) and H_3 (weak).

As is shown in **Figure S31**, H_5 has NOE signals with H_4 and H_6 (2.69 ppm). H_6 is a singlet and has no COSY signals with other 5 protons on the isoquinoline structure.

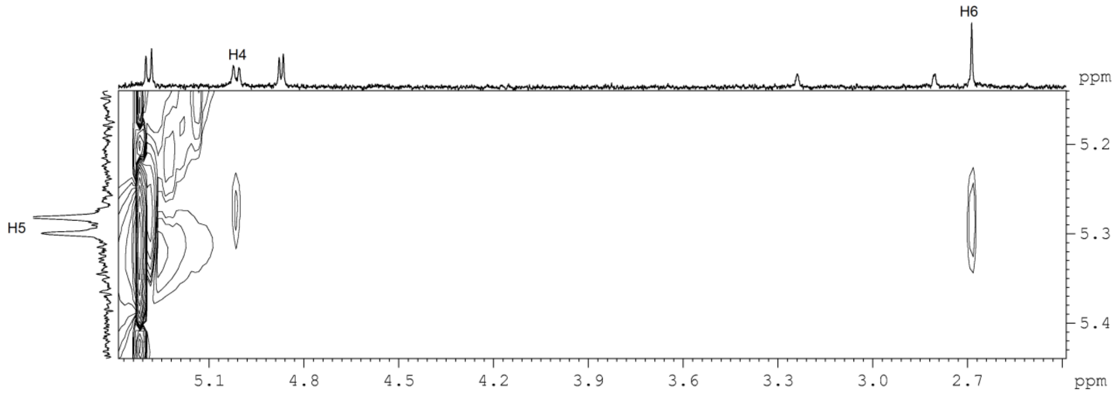

Figure S31. Correlation signals of H_5 - H_4 and H_5 - H_6 in ^{1}H-^{1}H NOESY spectrum (diffusion-edited ^{1}H spectrum projection of ***c*-P5·T5$_{Fc}$**, CD_2Cl_2, 500 MHz, 298 K).

As shown in **Figure S32**, the peaks at 3.24 ppm and 2.80 ppm belong to ferrocene protons, which have various NOE signals with isoquinoline protons. The *tert*-butyl protons (-0.25 ppm) on the ferrocene phosphine structure have NOE signals with H_{Fc1} (3.24 ppm); yet they do not have NOE signals with H_{Fc2} (2.80 ppm), which has longer distance in between them. Both H_{Fc1} and H_{Fc2} have NOE signals to H_3 and H_4, which are the isoquinoline protons close to the ferrocene core.

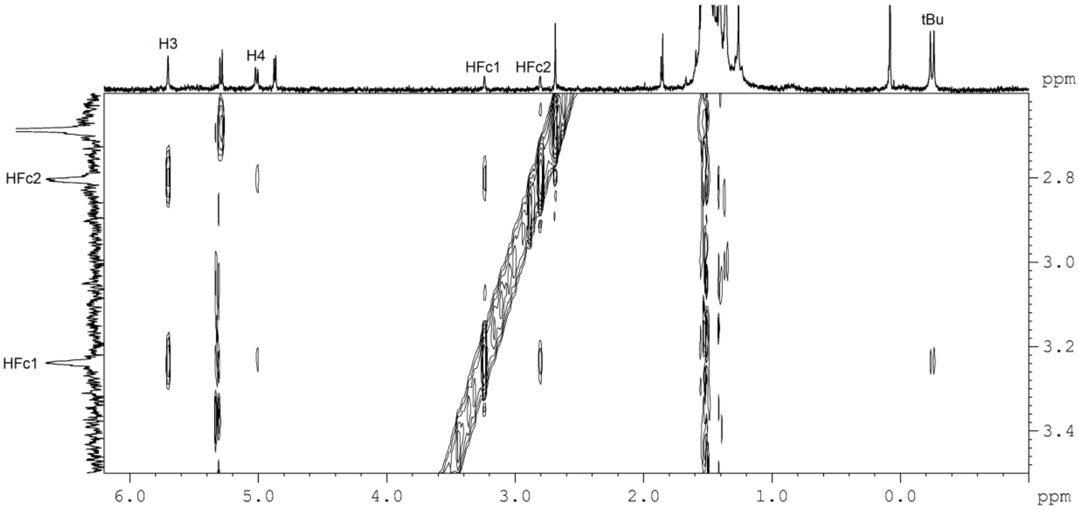

Figure S32. Correlation signals of H_{Fc1} - H_3, H_{Fc1} - H_4, H_{Fc1} - $H_{t\text{-}Bu}$, H_{Fc2} - H_3, H_{Fc2} - H_4 and H_{Fc1} - H_{Fc2} in ^1H-^1H NOESY spectrum (diffusion-edited ^1H spectrum projection of ***c*-P5·T5**$_{\mathbf{Fc}}$, CD_2Cl_2, 500 MHz, 298 K).

H_1 and H_6, which are the closest protons (in the template) to the porphyrins, has NOE signals with the beta- and only one (the one facing towards the inner side of the porphyrin ring structure) of the six aryl protons in the porphyrin.

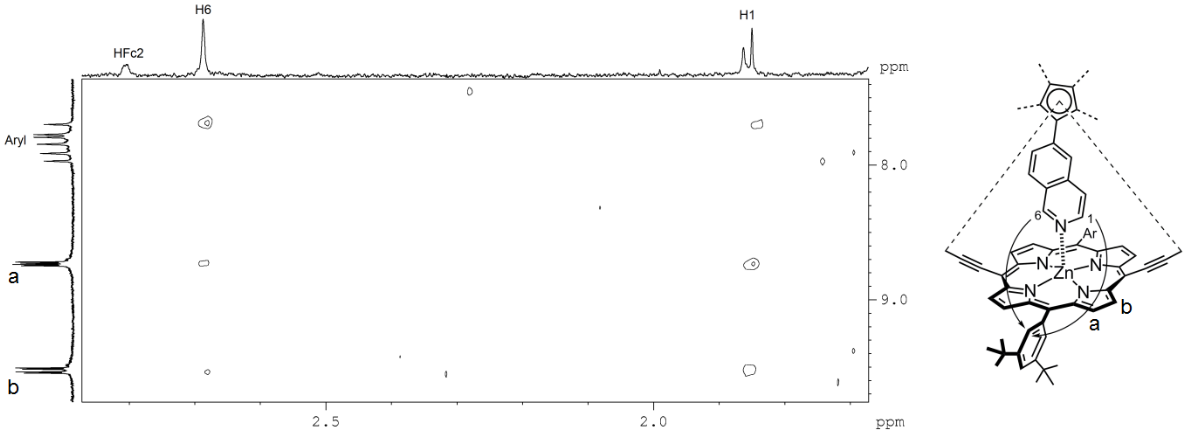

Figure S33. Correlation signals of H_1 and H_6 with the beta and only one of the six aryl-protons in ^1H-^1H NOESY spectrum (diffusion-edited ^1H spectrum projection of ***c*-P5·T5**$_{\mathbf{Fc}}$, CD_2Cl_2, 500 MHz, 298 K).

*C3. Characterization of **c-P5$_{OOct}$·T5$_{Fc}$***

***c*-P5$_{OOct}$·T5$_{Fc}$** has a very similar spectrum to ***c*-P5·T5$_{Fc}$**. Two doublets from beta-protons overlap with each other and a doublet appear at 8.80 ppm. More signals appear from the alkyl chains on the porphyrin: H_1 is overlapped under one of the alkyl chain signals at around 1.8 ppm. The ^{1}H-^{13}C COSY, ^{1}H-^{1}H COSY and ^{1}H-^{1}H NOESY spectra all give evidences for the overlap.

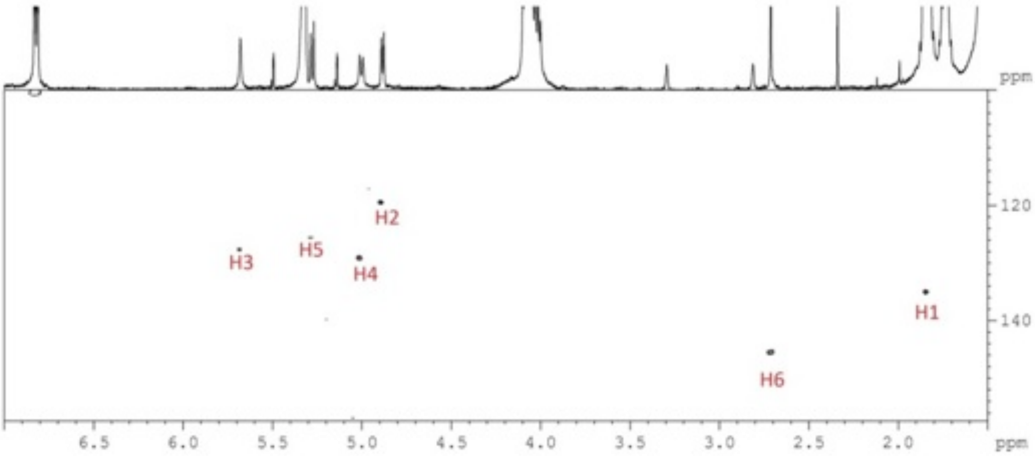

Figure S34. Identification of six isoquinoline protons from ^{1}H-^{13}C HSQC spectrum (CD_2Cl_2, 500 MHz, 298 K).

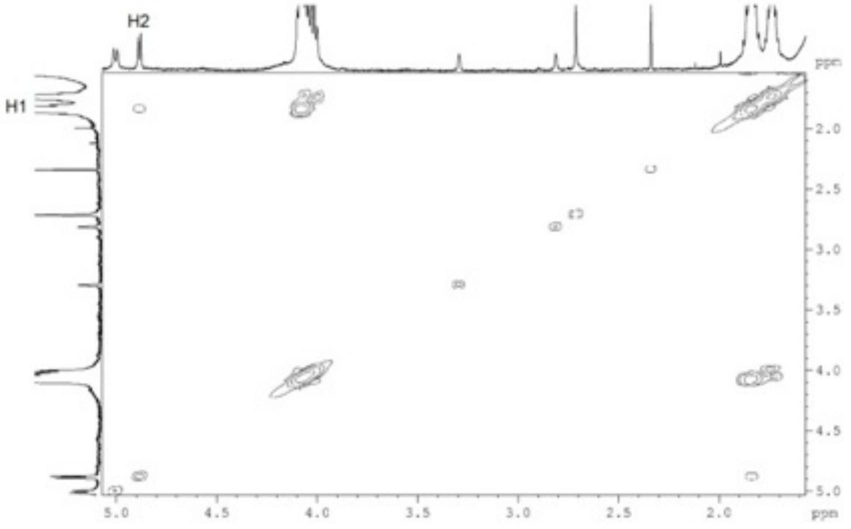

Figure S35. COSY signal between H_1 and H_2 (CD_2Cl_2, 500 MHz, 298 K).

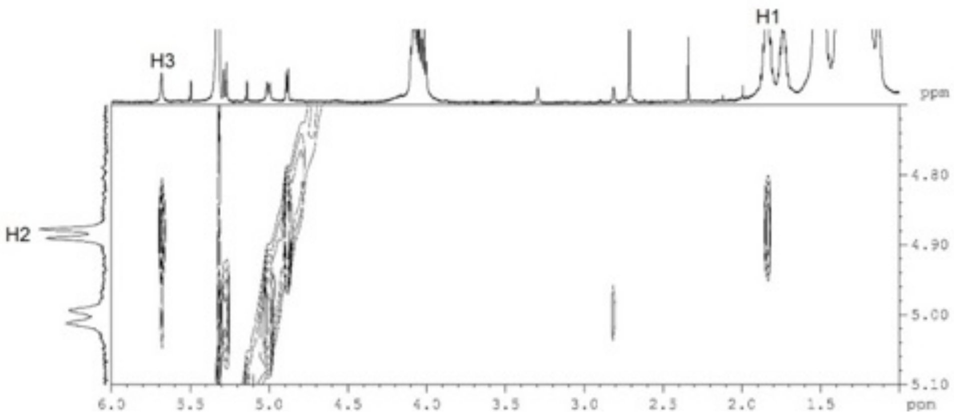

Figure S36. NOE signals of H_1 - H_2 and H_3 - H_2 (CD_2Cl_2, 500 MHz, 298 K).

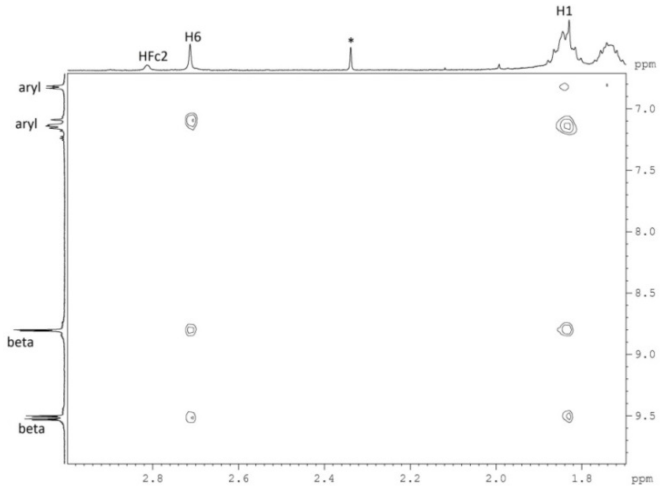

Figure S37. Correlation signals of H_1 and H_6 with the aryl and beta-protons in ^1H-^1H NOESY spectrum (CD_2Cl_2, 500 MHz, 298 K, * represents residual solvent or impurity).

*C4. Characterization of **c-P5·T5_{cor}***

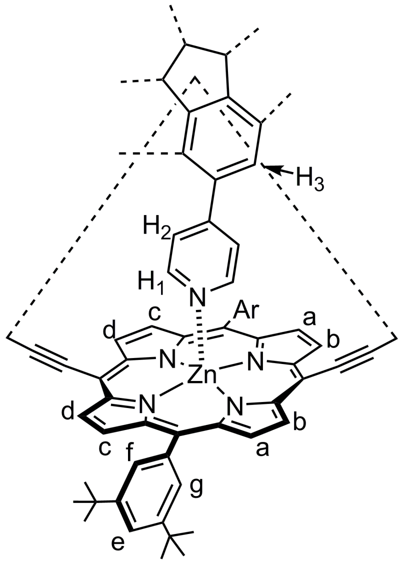

Figure S38. Designation of the protons in ***c*-P5·T5$_{cor}$**.

Compared to ***c*-P5·T5$_{Fc}$**, ***c*-P5·T5$_{cor}$** has a simpler template structure, which makes the designation of template signals easier.

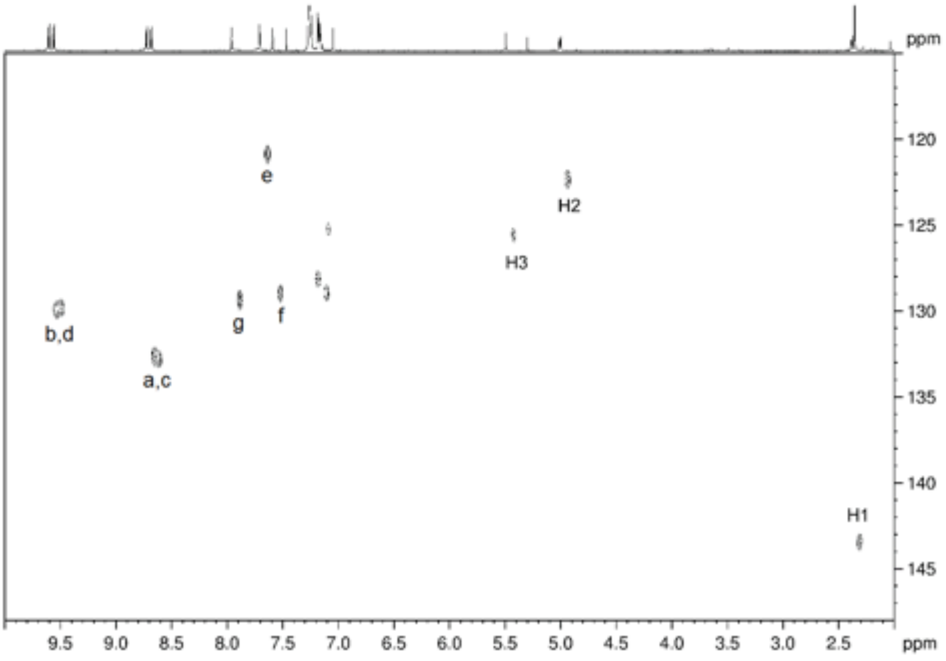

Figure S39. Identification of ***c*-P5·T5$_{cor}$** from ^{1}H-^{13}C HSQC spectrum ($CDCl_3$, 500 MHz, 298 K).

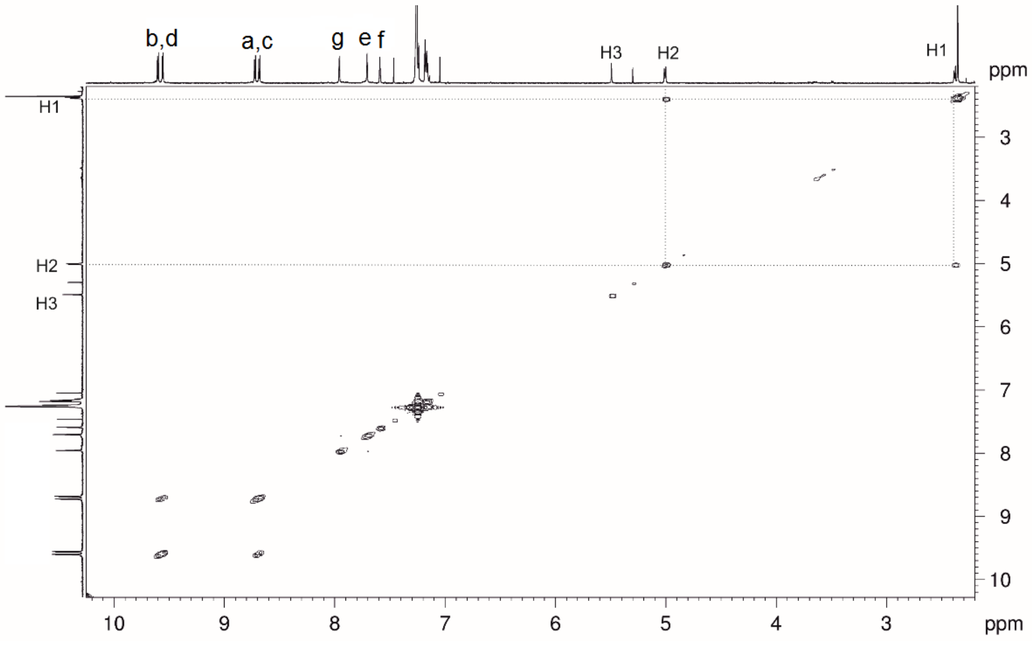

Figure S40. ^{1}H-^{1}H COSY spectrum of ***c*-P5·T5$_{cor}$** ($CDCl_3$, 500 MHz, 298 K).

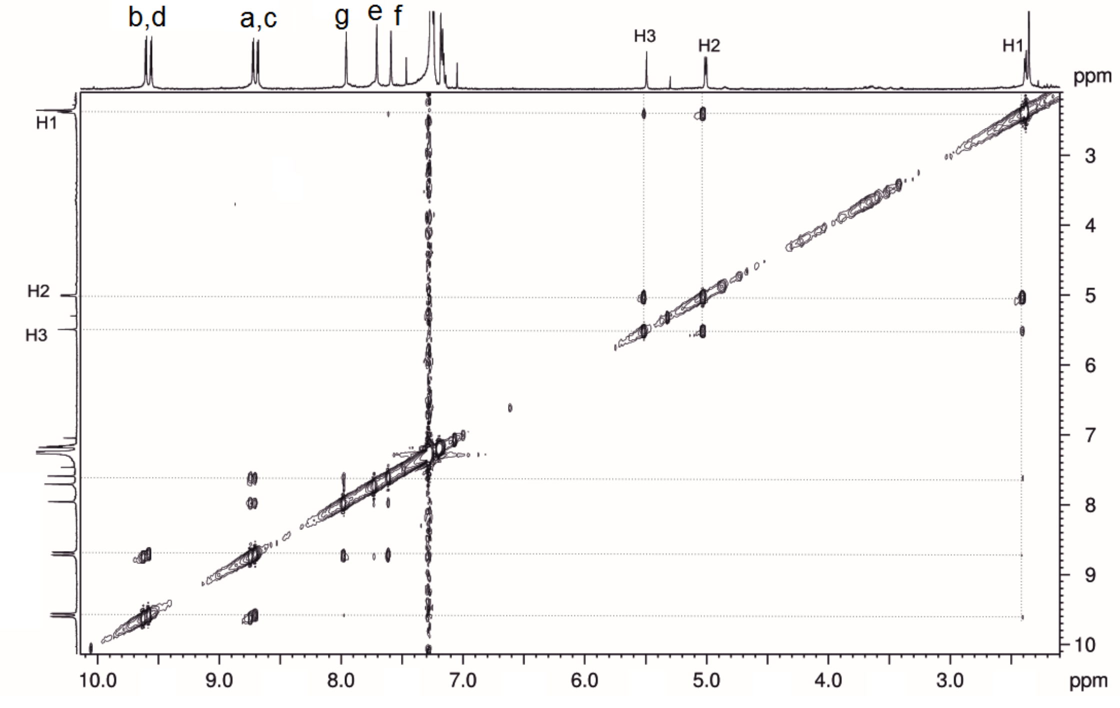

Figure S41. ^{1}H-^{1}H NOESY spectrum of ***c*-P5·T5$_{cor}$** ($CDCl_3$, 500 MHz, 298 K).

H1, which is the closest protons (in the template) to the porphyrins, has NOE signals with the beta- and only one (the one facing towards the inner side of the porphyrin ring structure) of the three aryl protons in the porphyrin (dashed line).

C5. The Shielding Effects of Porphyrin Nanoring

As is shown below, the shielding effect of the porphyrin nanoring can be reflected by the change of chemical shifts of the template protons between the free form and the nanoring-bound form.

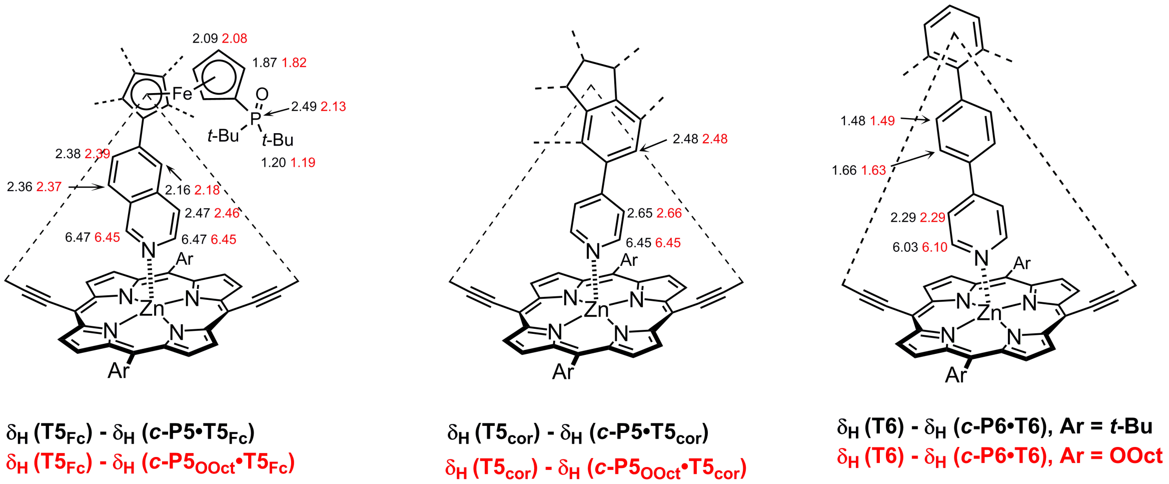

Figure S42. Chemical shift changes Δδ induced by the shielding effects of ***c*-P5·T5$_{Fc}$** and ***c*-P5·T5$_{cor}$**. The Δδ from previously reported ***c*-P6·T6** is shown as a comparison.[S5]

*C6. GPC Traces of **c-P5·T5$_{Fe}$** and **c-P5·T5$_{cor}$** Crude Reaction Mixtures*

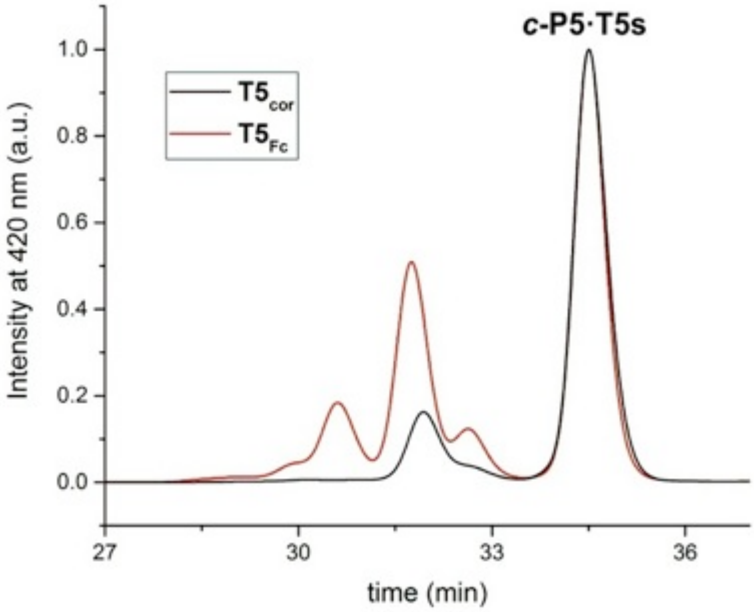

Figure S43. Normalized GPC traces (THF/pyridine, 100/1, *v*/*v*) of reaction mixture (after passing through aluminum column) of ***c*-P5** prepared using **T5$_{cor}$** and **T5$_{Fc}$** as templates. GPC column: JAIGEL 3H A (8 × 500 mm); flow rate: 1.0 mL/min.

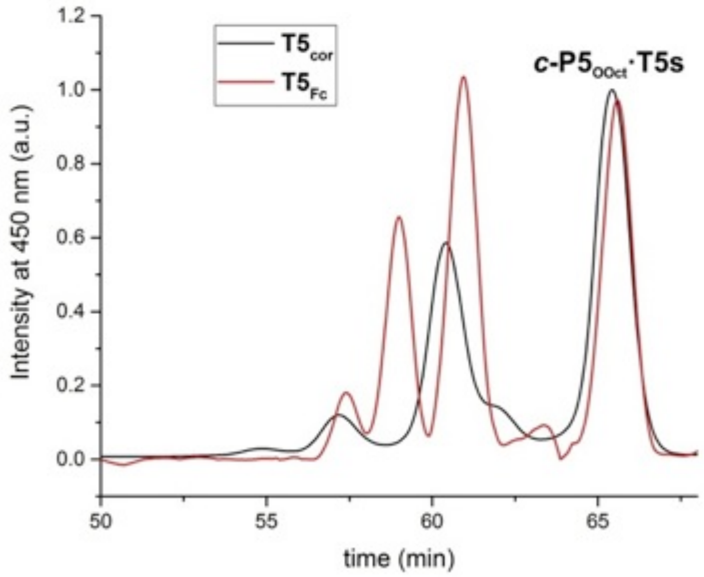

Figure S44. Normalized GPC traces (toluene/pyridine, 100/1, *v*/*v*) of reaction mixture (after passing through aluminum column) of ***c*-P5**$_{\mathbf{OOct}}$ prepared using **T5**$_{\mathbf{cor}}$ and **T5**$_{\mathbf{Fc}}$ templates (baseline corrected for **T5**$_{\mathbf{Fc}}$ trace). GPC column: JAIGEL 3H (20 × 600 mm) and JAIGEL 4H (20 × 600 mm) columns; flow rate: 3.5 mL/min.

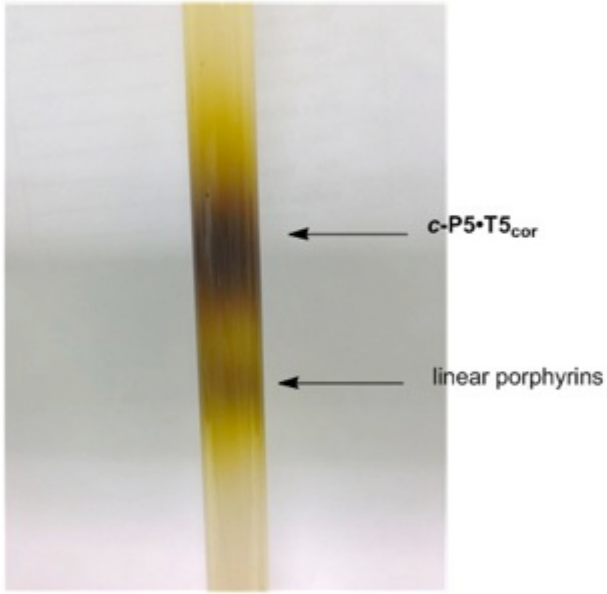

Figure S45. Picture of separation of ***c*-P5·T5**$_{\mathbf{cor}}$ from linear porphyrins by size exclusion column chromatography (toluene, Biobeads SX-1, 10 × 200 mm).

D. Fluorescent Properties

D1. Time-Resolved Photoluminescence Spectroscopy

To investigate photoluminescent (PL) decay dynamics, electronic gating through the time-correlated single-photon counting (TCSPC) technique was used (Becker & Hickl module). Here, the emission was detected with a silicon single-photon avalanche diode, yielding a temporal resolution of around 40 ps. By fitting the PL intensity (*I*) decay to a single exponential decay model, $I = Ae^{-t/\tau}$, the lifetime τ of the emission was extracted.

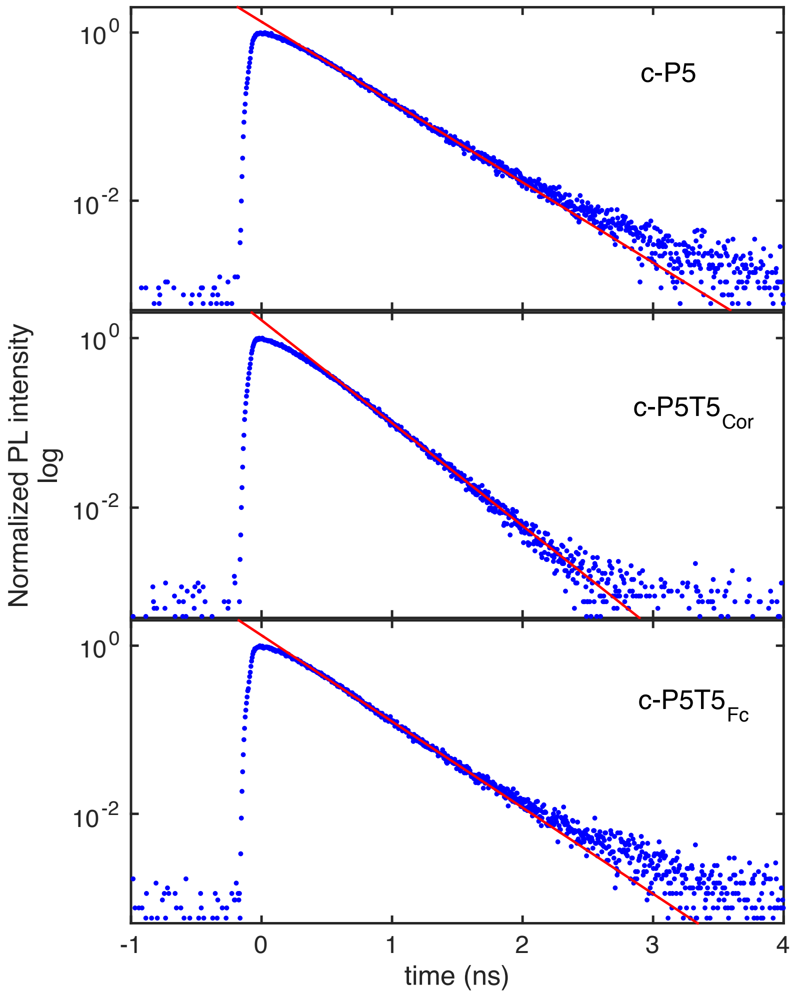

Figure S46. Normalized PL intensity decay dynamics of 5-porphyrin nanorings on log scale (blue) and single exponential fit to the intensity decay (red) recorded using TCSPC in toluene/1% pyridine solution at 450 nm excitation.

D2. Quantum Yield and Radiative Rates

As quantum yield (QY) measurements had been carried out on porphyrin nanorings in previous studies,[S6] a relative approach was adopted in this study using ***l*-P6** (see the reference for the compound structure) as a reference:

$$QY_{sample} = \frac{I_{sample}}{I_{ref}} \times \frac{Abs_{ref}(\lambda_{ext})}{Abs_{sample}(\lambda_{ext})} \times QY_{ref} \tag{S.1}$$

where I is the integrated area of PL emission spectrum at the excitation wavelength λ_{ext} and $Abs(\lambda_{ext})$ is the absorbance at λ_{ext}. The QY of the reference compound ***l*-P6** is 28%.

Using the obtained quantum yield and the overall decay rate Γ_{total} extracted from the PL transients ($\Gamma_{total} = 1/\tau$), the radiative (Γ_{rad}) and non-radiative (Γ_{nr}) rate were extracted using: $\Gamma_{rad} = QY \times \Gamma_{total}$ and $\Gamma_{total} = \Gamma_{rad} + \Gamma_{nr}$.

Table S1. Lifetime ($\boldsymbol{\tau}$), quantum yield (QY), radiative rate ($\boldsymbol{\Gamma_{rad}}$) and non-radiative rate ($\boldsymbol{\Gamma_{nr}}$) of 5-porphyrin nanorings. Lifetimes were extracted by measuring PL intensity dynamics using TCSPC with 450 nm excitation. PL quantum yields were measured with excitation at 500 nm.

Compound	$\boldsymbol{\tau}$ (ps)	QY	$\boldsymbol{\Gamma_{rad}}$ (1/ns)	$\boldsymbol{\Gamma_{nr}}$ (1/ns)
***c*-P5**	455	3.06%	0.067	2.13
***c*-P5·T5$_{cor}$**	372	0.61%	0.016	2.67
***c*-P5·T5$_{Fc}$**	418	0.89%	0.021	2.37

E. Effective Molarity Measurement

*E1. Titrations of Monodentate Ligands with **c-P5** (Reference Titrations)*

***c*-P5** was titrated with monodentate ligands (quinuclidine, pyridine and isoquinoline) to measure their association constants.

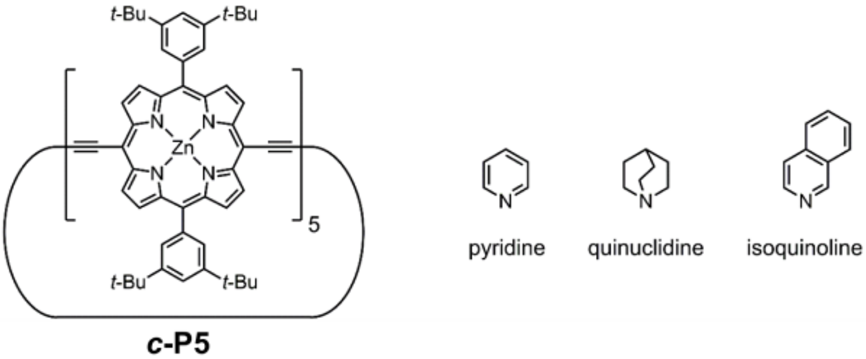

Figure S47. Ligands used for measurements of reference association constants of ***c*-P5**.

All the titrations were performed in toluene at 298 K and the concentration of porphyrin nanorings was 1.5 μM. All titrations were carried out at constant porphyrin concentration by adding porphyrin to the ligand stock solution before starting the titrations. Titration curves were fitted to a 1:1 binding isotherm using the equation:

$$\frac{A - A_{initial}}{\mathrm{A}_{\infty} - A_{initial}} = \left(\frac{(K_{\mathrm{a}}([\mathrm{L}] + [\mathrm{P}]_0) + 1) - \sqrt{(K_{\mathrm{a}}([\mathrm{L}] + [\mathrm{P}]_0) + 1)^2 - 4K_{\mathrm{a}}^2[\mathrm{P}]_0[\mathrm{L}]}}{2K_{\mathrm{a}}[\mathrm{P}]_0} \right) \quad \text{(S.2)}$$

where A is the observed absorption at a specific wavelength; $A_{initial}$ is the starting absorption at this wavelength; A_{∞} is the asymptotic final absorption at this wavelength; K_{a} is the association constant between ligand and porphyrin host; [L] is the total (free and bound) concentration of ligand; $[\mathrm{P}]_0$ is 5 × [***c*-P5**] (concentration of ***c*-P5**). The difference between absorptions at two different wavelengths can be substituted for each of the absorption parameters (A, $A_{initial}$, A_{∞}).

The results are listed in **Table S2** and figures below. In the stacked spectra, the bold black lines represent starting points and the red lines represent terminal points.

Table S2. Association constants of ***c*-P5** (1:1 association constants in M^{-1}, in toluene at 298 K).

Ligand	Binding constant	Run 1	Run 2	Average	Spectra
quinuclidine	K_{q}	(8.8 ± 0.6) × 10^5	(7.4 ± 0.8) × 10^5	(8.1 ± 0.8) × 10^5	**Figure S48** **Figure S49**
pyridine	K_{py}	(1.9 ± 0.1) × 10^4	(1.7 ± 0.1) × 10^4	(1.8 ± 0.1) × 10^4	**Figure S50** **Figure S51**
isoquinoline	K_{iso}	(2.4 ± 0.3) × 10^4	(2.2 ± 0.2) × 10^4	(2.3 ± 0.3) × 10^4	**Figure S52** **Figure S53**

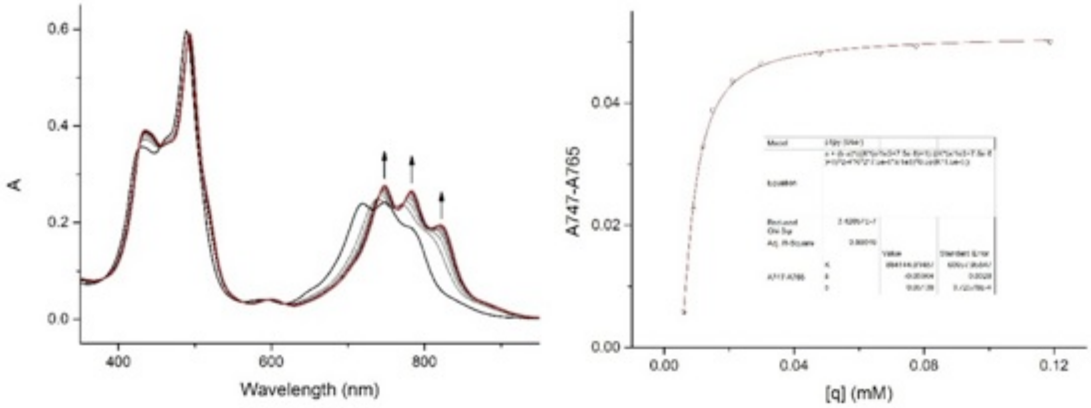

Figure S48. UV-vis-NIR titration results of quinuclidine and ***c*-P5**, $K_q = (8.8 \pm 0.6) \times 10^5\ M^{-1}$, $R^2 = 0.998$. (Run 1, toluene, 298 K, [***c*-P5**] = 1.5 μM).

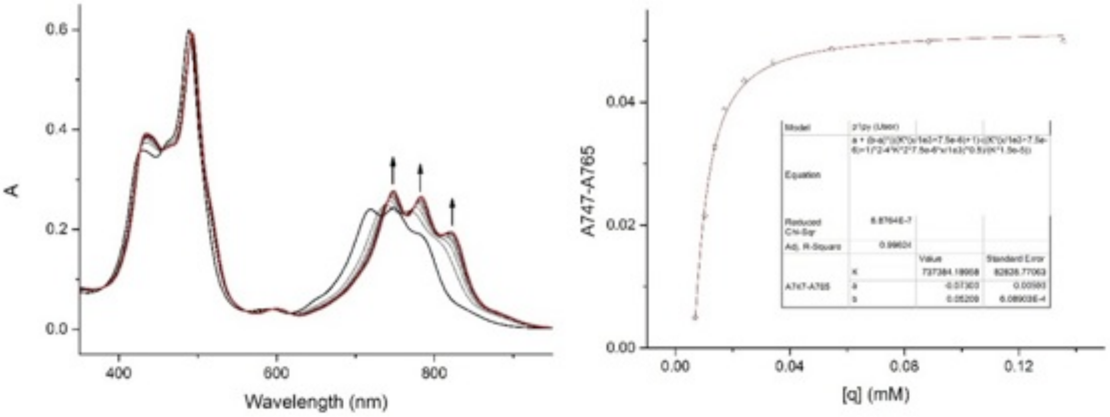

Figure S49. UV-vis-NIR titration results of quinuclidine and ***c*-P5**, $K_q = (7.4 \pm 0.8) \times 10^5\ M^{-1}$, $R^2 = 0.996$. (Run 2, toluene, 298 K, [***c*-P5**] = 1.5 μM).

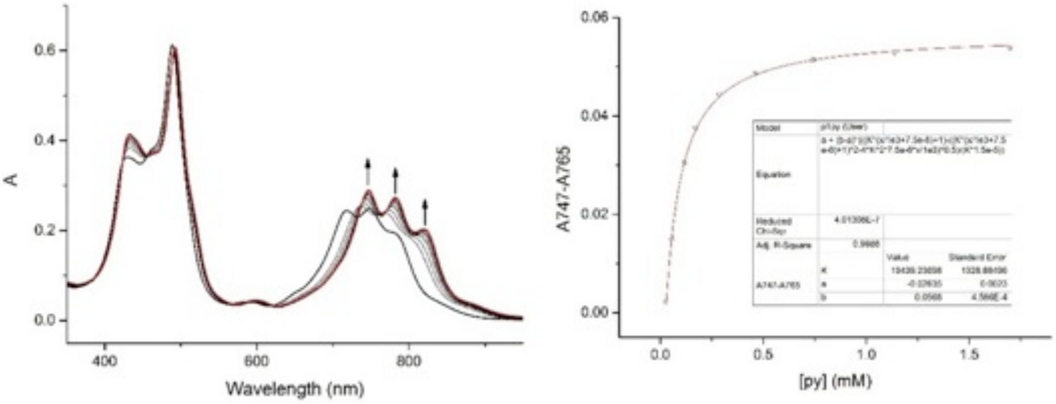

Figure S50. UV-vis-NIR titration results of pyridine and ***c*-P5**, $K_{py} = (1.9 \pm 0.1) \times 10^4\ M^{-1}$, $R^2 = 0.999$. (Run 1, toluene, 298 K, [***c*-P5**] = 1.5 μM).

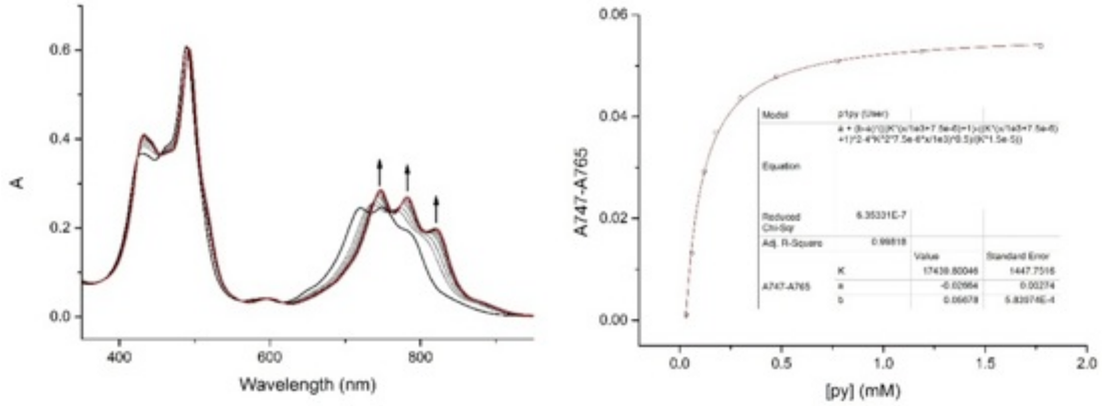

Figure S51. UV-vis-NIR titration results of pyridine and ***c*-P5**, $K_{py} = (1.7 \pm 0.1) \times 10^4$ M^{-1}, R^2 = 0.998. (Run 2, toluene, 298 K, [***c*-P5**] = 1.5 μM).

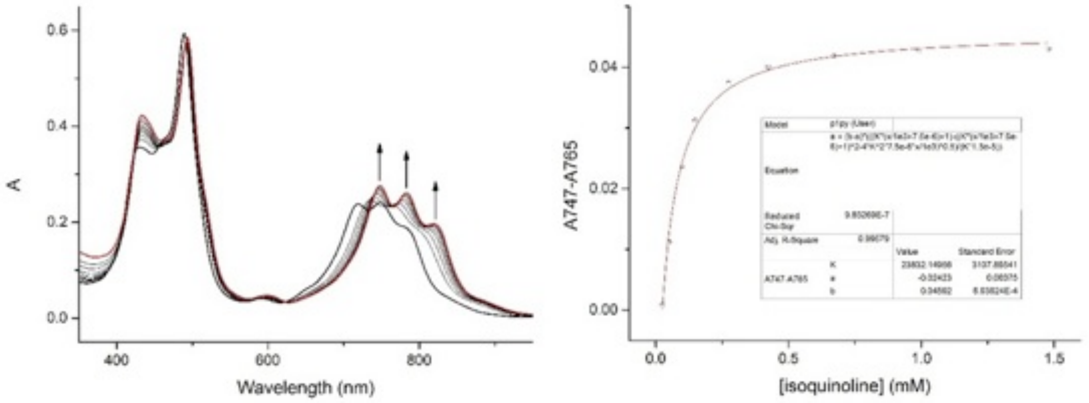

Figure S52. UV-vis-NIR titration results of isoquinoline and ***c*-P5,** $K_{iso} = (2.4 \pm 0.3) \times 10^4$ M^{-1}, R^2 = 0.996. (Run 1, toluene, 298 K, [***c*-P5**] = 1.5 μM).

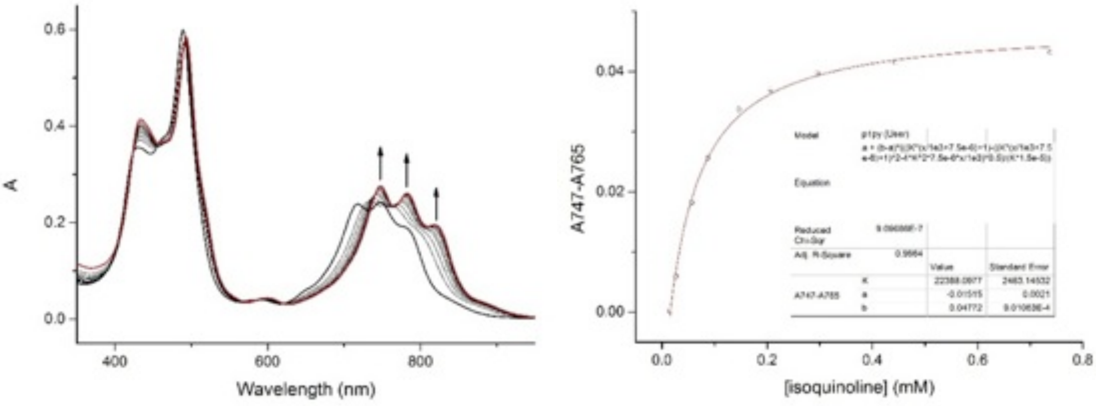

Figure S53. UV-vis-NIR titration results of isoquinoline and ***c*-P5**, $K_{iso} = (2.2 \pm 0.2) \times 10^4$ M^{-1}, R^2 = 0.996. (Run 2, toluene, 298 K, [***c*-P5**] = 1.5 μM).

*E2. Denaturation Titration of **c-P5·T5$_{Fc}$***

***c*-P5·T5$_{Fc}$** was titrated both with quinuclidine and pyridine. The corresponding denaturation constants are labeled as K_{dq} and K_{dpy} respectively.

All the titrations were performed in toluene at 298 K. All titrations were carried out at constant porphyrin nanoring concentration by adding porphyrin to the ligand stock solution before starting the titrations. All the data were fitted to the *n*-dentate breaking-up binding isotherm shown as below:

$$\frac{A - A_{initial}}{A_{\infty} - A_{initial}} = \left(\frac{-K_d[L]^n + \sqrt{K_d^2[L]^{2n} + 4K_d[L]^n[P]_0}}{2[P]_0}\right) \quad (S.3)$$

where A is the observed absorption at a specific wavelength; $A_{initial}$ is the starting absorption at a specific wavelength; A_{∞} is the terminal absorption at a specific wavelength; K_d is the dissociation constant between ligand and porphyrin nanoring complex; [L] is the concentration of ligand; $[P]_0$ is the concentration of porphyrin nanoring complex; n is the number of binding sites of nanoring complexes. For ***c*-P5·T5**, n = 5. The difference between absorptions at two different wavelengths can be substituted for each of the absorption parameters (A, $A_{initial}$, A_{∞}).

The titration results are listed in **Table S3** and figures below. In the stacked spectra, the bold black lines represent starting points and the red lines represent terminal points.

Table S3. Dissociation constants of ***c*-P5·T5$_{Fc}$** with quinuclidine and pyridine in toluene at 298 K.

Ligand	Dissociation constant	Run 1	Run 2	Average	Spectra
quinuclidine	K_{dq} (M^{-4})	(1.5 ± 0.1)	(1.6 ± 0.1)	(1.6 ± 0.1)	**Figure S54** **Figure S55**
pyridine	K_{dpy} (M^{-4})	(1.7 ± 0.1) × 10^{-8}	(1.6 ± 0.2) × 10^{-8}	(1.6 ± 0.2) × 10^{-8}	**Figure S56** **Figure S57**

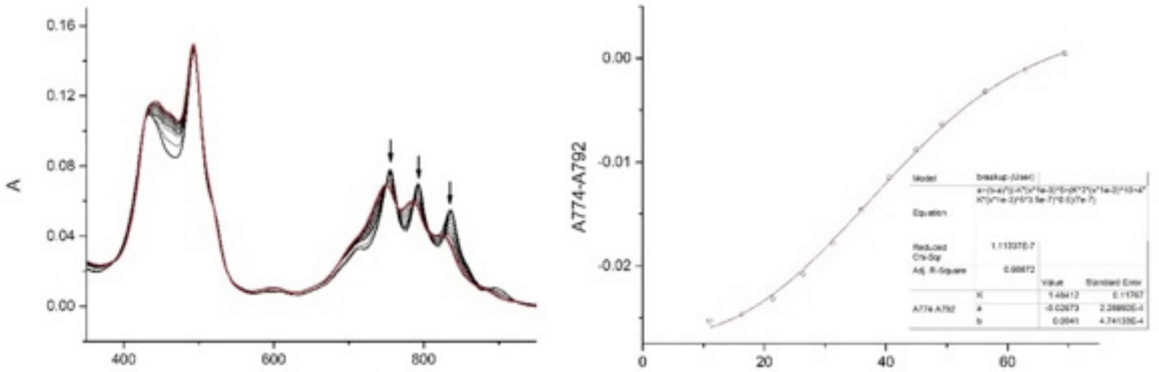

Figure S54. UV-vis-NIR titration results of quinuclidine and ***c*-P5·T5$_{Fc}$**, K_{dq} = (1.5 ± 0.1) M^{-4}, R^2 = 0.999. (Run 1, toluene, 298 K, [***c*-P5·T5$_{Fc}$**] = 0.35 μM).

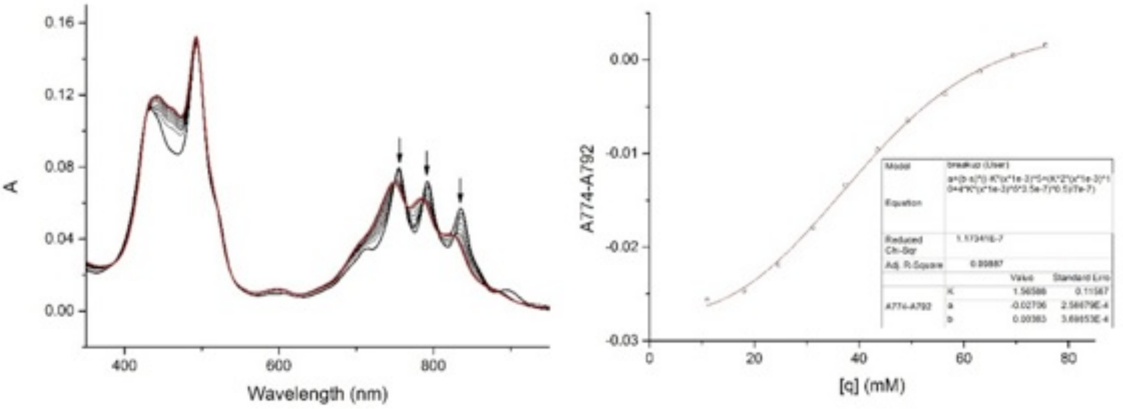

Figure S55. UV-vis-NIR titration results of quinuclidine and ***c*-P5·T5$_{\mathbf{Fc}}$**, $K_{dq} = (1.6 \pm 0.1)$ M^{-4}, $R^2 = 0.999$. (Run 2, toluene, 298 K, [***c*-P5·T5$_{\mathbf{Fc}}$**] = 0.35 μM).

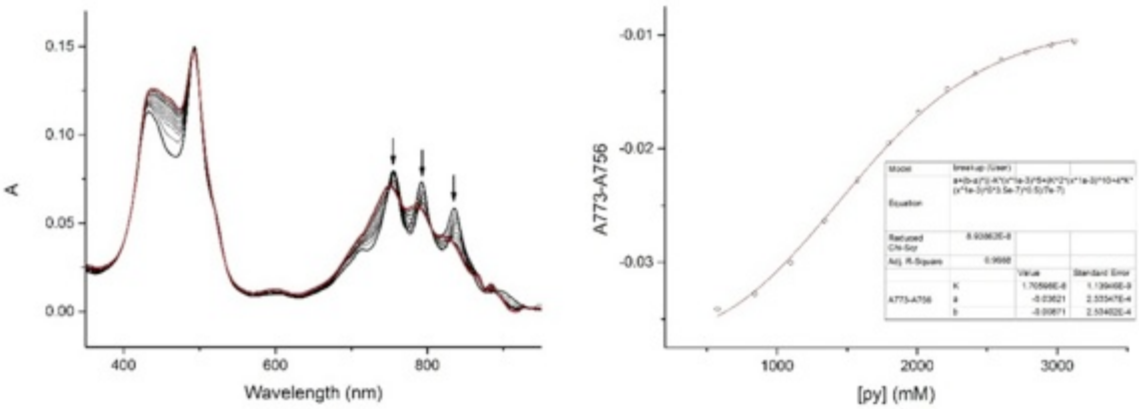

Figure S56. UV-vis-NIR titration results of pyridine and ***c*-P5·T5$_{\mathbf{Fc}}$**, $K_{dpy} = (1.7 \pm 0.1) \times 10^{-8}$ M^{-4}, $R^2 = 0.999$. (Run 1, toluene, 298 K, [***c*-P5·T5$_{\mathbf{Fc}}$**] = 0.35 μM).

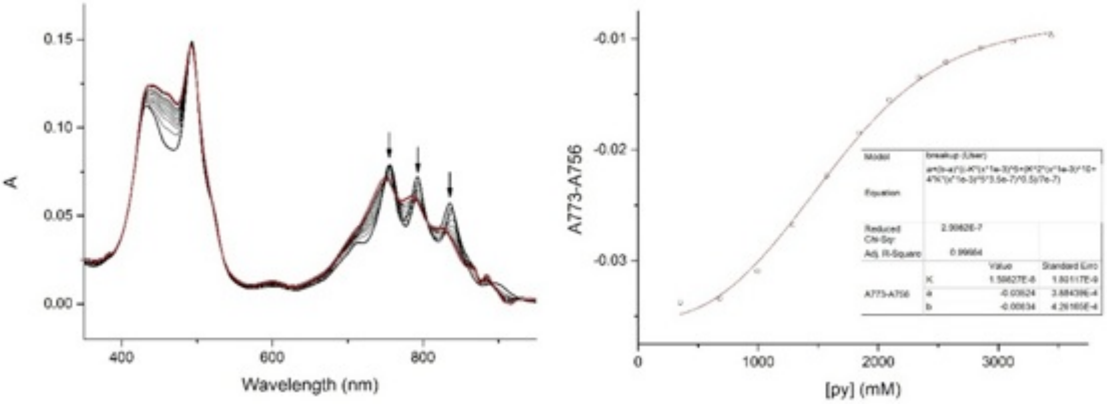

Figure S57. UV-vis-NIR titration results of pyridine and ***c*-P5·T5$_{\mathbf{Fc}}$**, $K_{dpy} = (1.6 \pm 0.2) \times 10^{-8}$ M^{-4}, $R^2 = 0.997$. (Run 2, toluene, 298 K, [***c*-P5·T5$_{\mathbf{Fc}}$**] = 0.35 μM).

*E3. Denaturation Titration of **c-P5·T5**$_{cor}$*

***c*-P5·T5**$_{\mathbf{cor}}$ was titrated with pyridine. The corresponding denaturation constant was labeled as K_{dpy}. All the titrations were performed in toluene at 298 K. All titrations were carried out at constant porphyrin nanoring concentration by adding porphyrin to the ligand stock solution before titrations started. All the data were fitted to the *n*-dentate breaking-up binding isotherm shown as (S.3).

The titration results are listed in **Table S4** and figures below. In the spectra, the bold black lines represent starting points and the red lines represent terminal points.

Table S4. Dissociation constants of ***c*-P5·T5**$_{\mathbf{cor}}$ with pyridine.

Ligand	Dissociation constant	Run 1	Run 2	Average	Spectra
pyridine	K_{dpy} (M^{-4})	$(7.6 \pm 0.5) \times 10^{-8}$	$(2.7 \pm 0.2) \times 10^{-8}$	$(5.1 \pm 0.3) \times 10^{-8}$	**Figure S58**<br>**Figure S59**

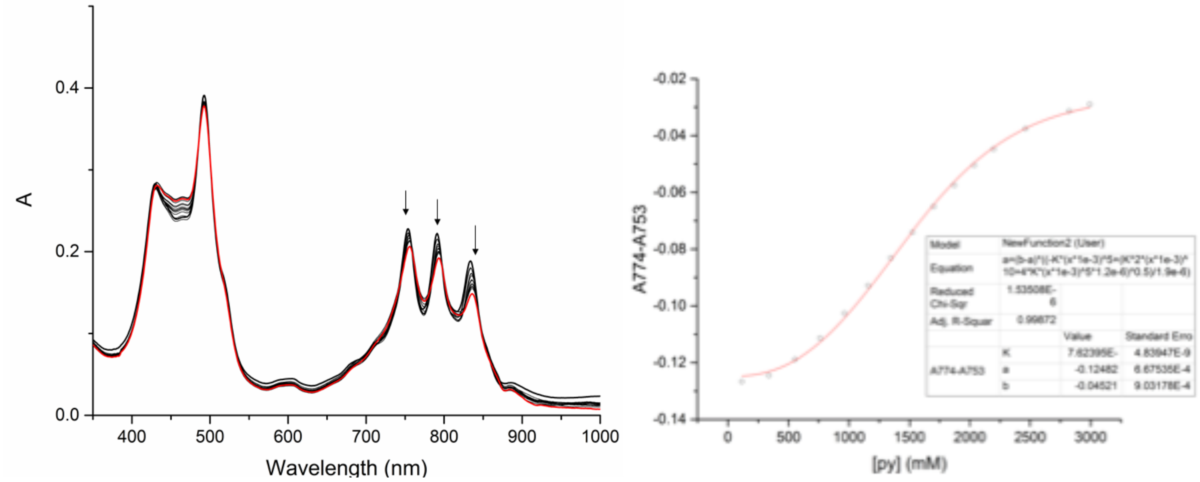

Figure S58. UV-vis-NIR titration results of pyridine and ***c*-P5·T5**$_{\mathbf{cor}}$, $K_{\mathrm{dpy}} = (7.6 \pm 0.5) \times 10^{-8}$ M^{-4}, $R^2 = 0.999$. (Run 1, toluene, 298 K, [***c*-P5·T5**$_{\mathbf{cor}}$] = 0.95 μM).

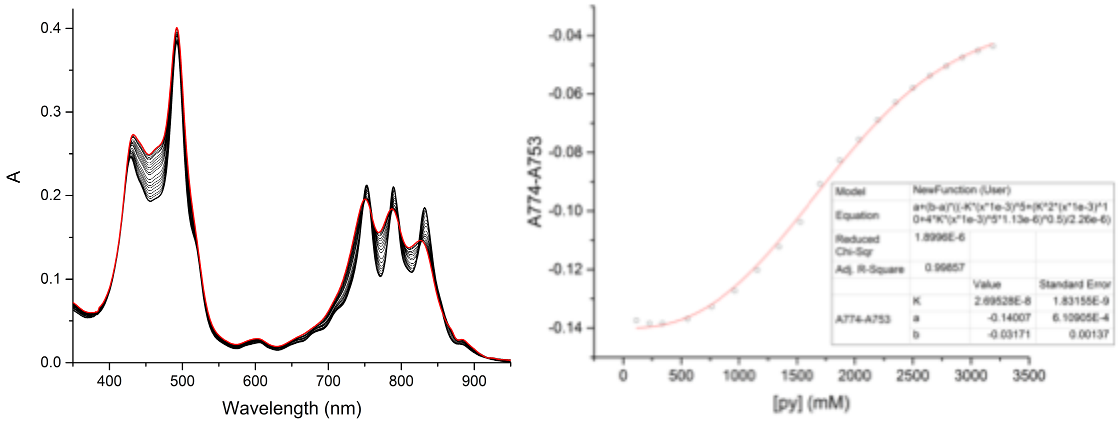

Figure S59. UV-vis-NIR titration results of pyridine and ***c*-P5·T5**$_{\mathbf{cor}}$**,** $K_{\mathrm{dpy}} = (2.7 \pm 0.2) \times 10^{-8}$ M^{-4}, $R^2 = 0.999$. (Run 2, toluene, 298 K, [***c*-P5·T5**$_{\mathbf{cor}}$] = 1.13 μM).

E4. Calculation of Effective Molarities

The formation constants K_f of ***c*-P5·T5$_{Fc}$** and ***c*-P5·T5$_{cor}$** were calculated from equation (S.4):

$$\log K_f = \log \frac{{K_L}^n}{K_{d,L}} = n\log K_L - \log K_{d,L} \tag{S.4}$$

where K_L is the association constants of the template-free ring with ligand L; $K_{d,L}$ is the denaturation constants of the nanoring-template complex with this ligand; n is the chelation number of nanoring-template complexes. For ***c*-P5·T5**, n = 5. The uncertainty in logK_f was calculated from equation (S.5):

$$\Delta(\log K_f) = \frac{1}{\ln 10}\sqrt{\left(\frac{n\Delta K_L}{K_L}\right)^2 + \left(\frac{\Delta K_{d,L}}{K_{d,L}}\right)^2} \tag{S.5}$$

where ΔK_L is the uncertainty of K_L and $\Delta K_{d,L}$ is the uncertainty of $K_{d,L}$.

The resulting values of logK_f are listed in **Table S5**:

Table S5. Formation constants of nanoring-template complexes (logarithms are decadic).

Nanoring	Denaturation ligand	Calculated logK_f
***c*-P5·T5$_{Fc}$**	quinuclidine	29.3 ± 0.2
***c*-P5·T5$_{Fc}$**	pyridine	29.1 ± 0.1
***c*-P5·T5$_{cor}$**	pyridine	28.5 ± 0.1

The geometric average effective molarities ($\overline{\text{EM}}$) of the nanoring complexes were calculated from equation (S.6):

$$\log \overline{\text{EM}} = \log \sqrt[n-1]{\frac{K_{\text{chem},n}}{K_1^n}} = \log \sqrt[n-1]{\frac{K_f}{K_\sigma K_1^n}} = \frac{(\log K_f - \log K_\sigma - n\log K_1)}{(n-1)} \tag{S.6}$$

where $K_{\text{chem},n}$ is the statistically corrected value of K_f. As is shown in **Figure S60**, for both ***c*-P5·T5$_{Fc}$** and ***c*-P5·T5$_{cor}$**, K_σ = 320. K_1 is the reference single-site microscopic binding constant statistically corrected for binding to one face of a porphyrin (i.e. $K_1 = 0.5K_{\text{iso}}$, half of the binding constants measured in reference association titrations in **Section E1**). The uncertainties in the values of log$\overline{\text{EM}}$ were calculated from equation (S.7):

$$\Delta(\log EM) = \frac{1}{(n-1)}\sqrt{\left(\Delta(\log \Delta K_f)\right)^2 + \left(\frac{n\Delta K_1}{\ln 10 K_1}\right)^2} \tag{S.7}$$

The geometric average effective molarities of the nanoring complexes are listed in **Table S6**:

Table S6. Geometric average effective molarities of ***c*-P5·T5$_{Fc}$** and ***c*-P5·T5$_{cor}$** (data generated from pyridine denaturation ligand; logarithms are decadic).

nanoring	logK_f	log$\overline{EM}$	$\overline{EM}$ (M)
***c*-P5·T5$_{Fc}$**	29.1 ± 0.1	1.57 ± 0.07	41 ± 9
***c*-P5·T5$_{cor}$**	28.5 ± 0.1	1.55 ± 0.02	36 ± 5

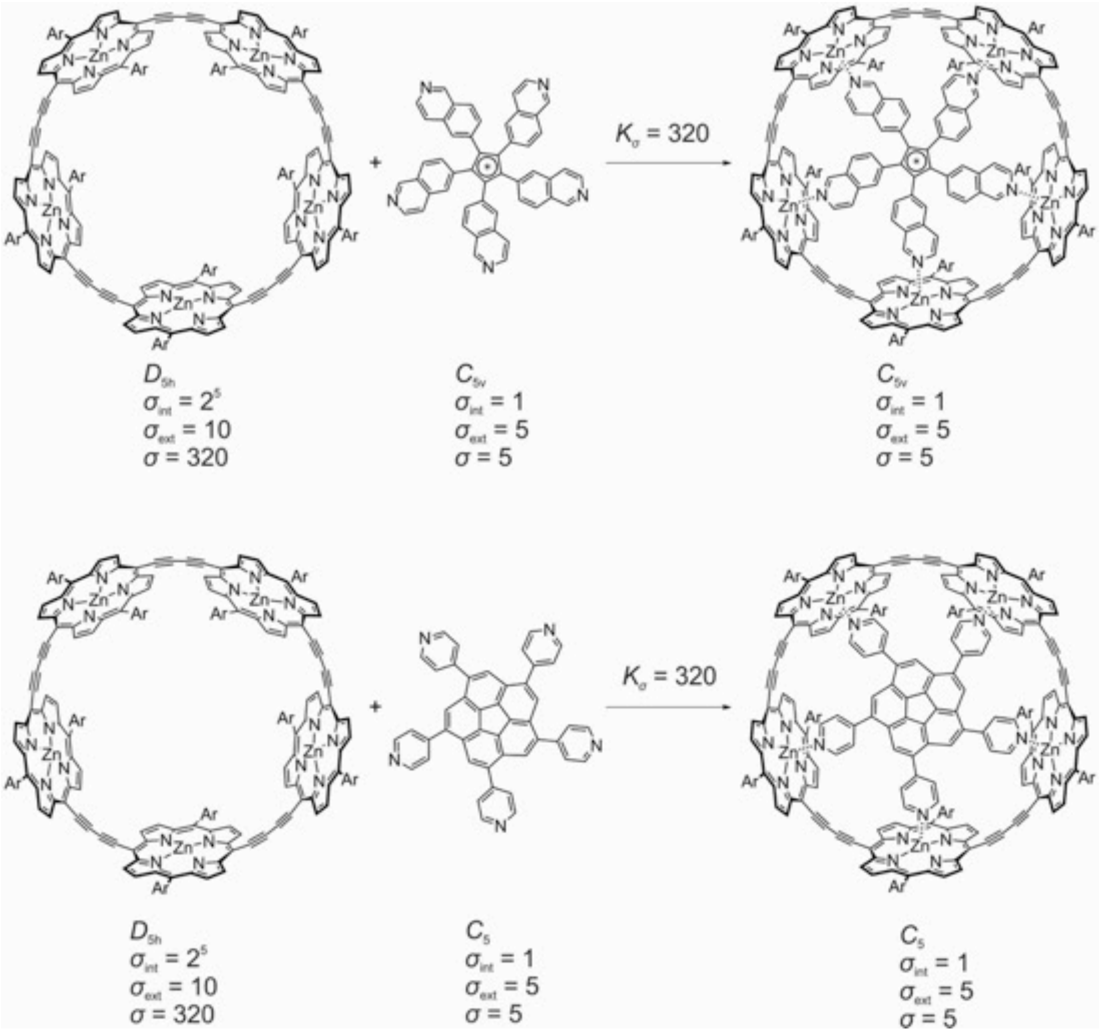

Figure S60. Statistical factors of ***c*-P5·T5$_{Fc}$** and ***c*-P5·T5$_{cor}$**.

F. Computational details

Molecular models were built in HyperChem v. 7.0 using the MM+ forcefield with parameters modified for the description of porphyrins.[S7] The porphyrin *meso* aryl groups were replaced with H, as was the di-*tert*-butyl phosphine oxide moiety on **T5$_{\mathbf{Fc}}$**. The Polak-Ribiere and steepest descent algorithms were used for geometry optimizations. A twisted and planar conformation of each of ***c*-P5•T5$_{\mathbf{cor}}$** and ***c*-P5•T5$_{\mathbf{Fc}}$** were prepared in HyperChem. The resulting geometries were subjected to further optimization in MOPAC2016 using the PM6 level of theory, and in the absence of symmetry.[S8] The eigenvector following minimization routine was used for the geometry optimization. For ***c*-P5•T5$_{\mathbf{cor}}$** the conformation of the starting HyperChem geometry (twisted or planar) was retained in the PM6-optimized structure. Both twisted and planar PM6 structures had no negative frequencies in force calculations. In contrast, both starting ***c*-P5•T5$_{\mathbf{Fc}}$** structures converged to similar geometries.

DFT geometry optimizations were performed using Gaussian 09/D.01 with the B3LYP functional and the 6-31G* basis set and an empirical dispersion correction recommended by Grimme (GD3).[S9]

For ***c*-P5•T5$_{\mathbf{cor}}$**, both twisted and planar PM6 geometries were used as starting points, the latter in C_5 symmetry. With DFT, both starting structures ultimately converged to similar twisted structures, with C_1 symmetry. Correspondingly, the geometry optimization from the twisted PM6 guess was remarkably quick (299 CPU hours) compared to the C_5 symmetric planar PM6 guess (892 CPU hours).[S10] The lower energy of the two DFT structures (ΔE = 2 kJ mol^{-1}) was subjected to a frequency calculation (666 CPU hours), revealing no imaginary frequencies.

For ***c*-P5•T5$_{\mathbf{Fc}}$**, the MOPAC geometry was similarly used as a starting point for DFT geometry optimization (1211 CPU hours). The frequency calculation on ***c*-P5•T5$_{\mathbf{Fc}}$** revealed a very small imaginary mode (1.1 cm^{-1}), to which we attribute no significance.

The free templates **T5$_{\mathbf{cor}}$** and **T5$_{\mathbf{Fc}}$** were optimized by DFT (B3LYP/6-31G* level of theory), each in C_5 symmetry, following pre-optimization of a HyperChem model in MOPAC by PM6. Frequency calculations confirmed the minimum nature of the stationary point, with no imaginary frequencies.

Key structural parameters are reproduced in Table S7. r_{N} and r_{Zn} refer to the radii of the N-donor and Zn-acceptor sets of the molecules. oop$_{\mathrm{Zn}}$ (out of plane) refers to the root mean squared (RMS) deviation of the Zn atoms in the complex from coplanarity.

Table S7. Key structural parameters from DFT.

	r_{N} (Å)	r_{Zn} (Å)	oop$_{\mathrm{Zn}}$ (Å)	Energy (a.u., Hartree atomic units)
**T5$_{\mathbf{Fc}}$**	7.73	–	–	–3654.5124733
**T5$_{\mathbf{cor}}$**	7.37	–	–	–2003.6958672
***c*-P5**	–	10.61	0.00	– 14593.7475707
***c*-P5•T5$_{\mathbf{Fc}}$**	7.72	9.88	0.43	– 18248.4589226
***c*-P5•T5$_{\mathbf{cor}}$**	7.54	9.71	0.67	– 16597.6259661

Coordinates of optimized geometries and final SCF energies are given in xyz files in the SI .ZIP file.

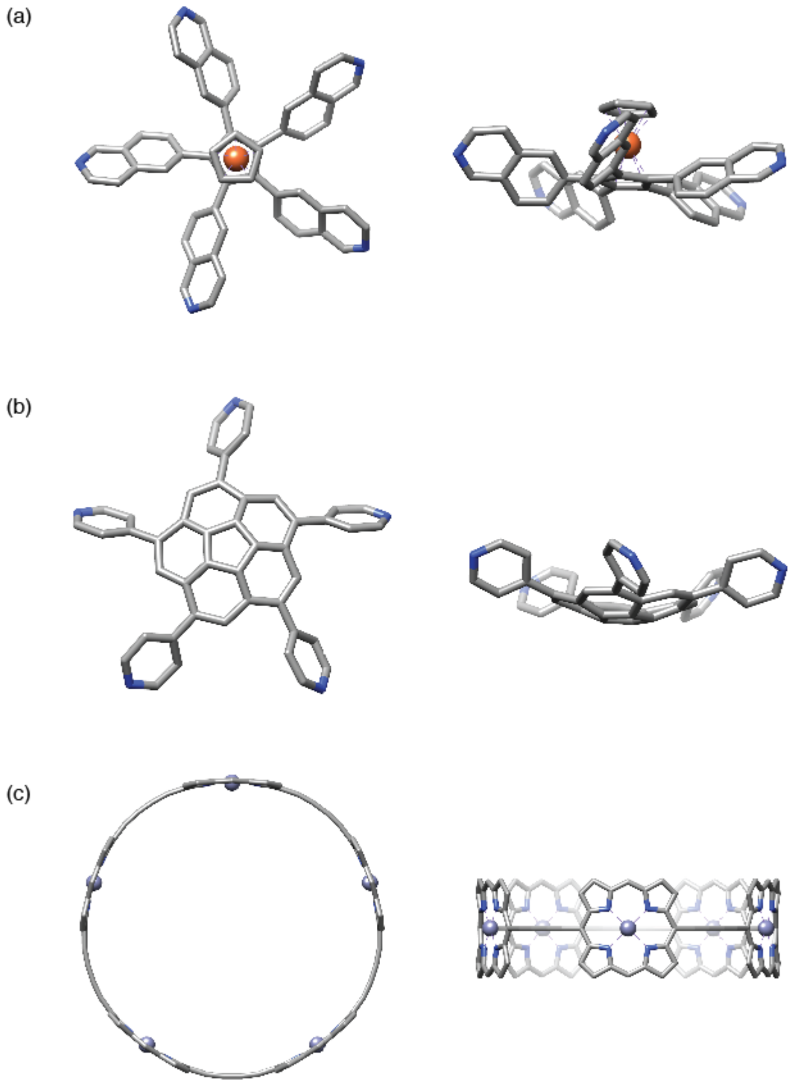

Figure S61. Geometry optimized DFT (B3LYP/6-31G*, GD3) structures for (a) $\mathbf{T5_{Fc}}$, (b) $\mathbf{T5_{cor}}$ and (c) ***c*-P5** (aryl groups truncated to –H).

The strain energy in ***c*-P5** was estimated by a homodesmotic reaction (Figure **S62** and Table **S8**) at the B3LYP/6-31G* level of theory, using the D3 dispersion correction.

A post-hoc correction with Grimme's gCP-D3(BJ) method,[S11] applied to B3LYP/6-31G* geometries calculated without iterant application of the D3 correction, did not significantly affect the result.

Similarly, the strain energy was not significantly changed when we used diphenylbutadiyne (Ph-C≡C-C≡C-Ph) in place of ***l*-P2** and the corresponding phenylacetylene capped ***l*-P5** in place of ***l*-P7** (in Figure **S62**).

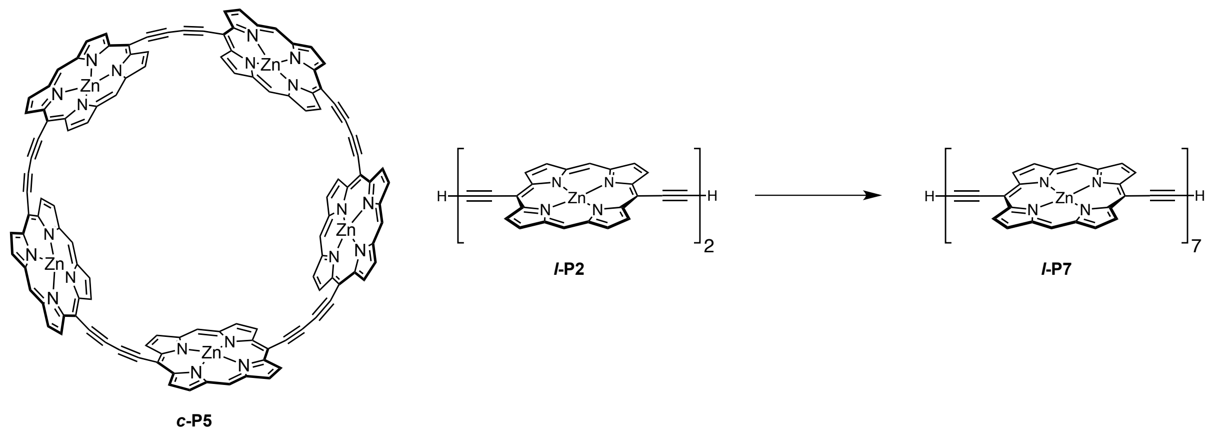

Figure S62. Homodesmotic reaction of ***c*-P5** with ***l*-P2** to give ***l*-P7**, for calculation of ring strain.

Table S8. Calculation of strain energy in ***c*-P5** (B3LYP/6-31G*).

	Energy (a.u., Hartree atomic units)
***c*-P5**	–14593.7475707
***l*-P2**	–5838.6796962
***l*-P7**	–20432.4733165
Strain energy = – (***l*-P7** – ***c*-P5** – ***l*-P2**) = 120.9 kJ mol^{-1}	

G. References

S1 C. A. Busacca, M. C. Eriksson, N. Haddad, Z. S. Han, J. C. Lorenz, B. Qu, X. Zeng, C. H. Senanayake, *Org. Synth.* **2013**, *90*, 316–326.

S2 (a) M. N. Eliseeva, L. T. Scott, *J. Am. Chem. Soc.* **2012**, *134*, 15169–15172. (b) K. Kawasumi, Q. Zhang, Y. Segawa, L. T. Scott, K. Itami, *Nat. Chem.* **2013**, *5*, 739–744

S3 P. N. Taylor, H. L. Anderson, *J. Am. Chem. Soc.* **1999**, *121*, 11538–11545.

S4 M. Hoffmann, C. J. Wilson, B. Odell, H. L. Anderson, *Angew. Chem. Int. Ed.* **2007**, *46*, 3122–3125.

S5 M. Hoffmann, J. Kärnbratt, M.-H. Chang, L. M. Herz, B. Albinsson, H. L. Anderson, *Angew. Chem. Int. Ed.* **2008**, *47*, 4993–4996.

S6 C.-K. Yong, P. Parkinson, D. V. Kondratuk, W.-H. Chen, A. Stannard, A. Summerfield, J. K. Sprafke, M. C. O'Sullivan, P. H. Beton, H. L. Anderson, L. M. Herz, *Chem. Sci.* **2015**, *6*, 181–189.

S7 Parameters from the following papers were used: H. M. Marques, I. Cukrowski, *Phys. Chem. Chem. Phys.* **2003**, *5*, 5499–5506; H. M. Marques, I. Cukrowski, *Phys. Chem. Chem. Phys*. **2002**, *4*, 5878–5887; E. Goldstein, B. Ma, J.-H. Lii, N. L. Allinger, *J. Phys. Org. Chem.* **1996**, *9*, 191–202; P. D. Jarowski, F. Diederich, K. N. Houk, *J. Org. Chem*. **2005**, *70*, 1671–1678.

S8 MOPAC2016, J. J. P. Stewart, Stewart Computational Chemistry, Colorado Springs, CO, USA, 2016; J. J. P. Stewart, *J. Mol. Modeling* **2007**, *13*, 1173–1213.

S9 A. D. Becke, *J. Chem. Phys.* **1993**, *98*, 5648; P. J. Stephens, F. J. Devlin, C. F. Chabalowski, M. J. Frisch, *J. Phys. Chem.* **1994**, *98* 11623–11627; V. A. Rassolov, J. A. Pople, M. A. Ratner and T. L. Windus, *J. Chem. Phys.* **1998**, *109*, 1223; P. C. Hariharan, J. A. Pople, *Theor. Chim. Acta*, **1973**, *28*, 213–222; W. J. Hehre, R. Ditchfield, J. A. Pople, *J. Chem. Phys.* **1972**, *56*, 2257; R. Ditchfield, W. J. Hehre, J. A. Pople, *J. Chem. Phys.* **1971**, *54*, 724; M. J. Frisch, G. W. Trucks, H. B. Schlegel, G. E. Scuseria, M. A. Robb, J. R. Cheeseman, G. Scalmani, V. Barone, B. Mennucci, G. A. Petersson, H. Nakatsuji, M. Caricato, X. Li, H. P. Hratchian, A. F. Izmaylov, J. Bloino, G. Zheng, J. L. Sonnenberg, M. Hada, M. Ehara, K. Toyota, R. Fukuda, J. Hasegawa, M. Ishida, T. Nakajima, Y. Honda, O. Kitao, H. Nakai, T. Vreven, J. A. Montgomery Jr., J. E. Peralta, F. Ogliaro, M. Bearpark, J. J. Heyd, E. Brothers, K. N. Kudin, V. N. Staroverov, R. Kobayashi, J. Normand, K. Raghavachari, A. Rendell, J. C. Burant, S. S. Iyengar, J. Tomasi, M. Cossi, N. Rega, J. M. Millam, M. Klene, J. E. Knox, J. B. Cross, V. Bakken, C. Adamo, J. Jaramillo, R. Gomperts, R. E. Stratmann, O. Yazyev, A. J. Austin, R. Cammi, C. Pomelli, J. W. Ochterski, R. L. Martin, K. Morokuma, V. G. Zakrzewski, G. A. Voth, P. Salvador, J. J. Dannenberg, S. Dapprich, A. D. Daniels, Ö. Farkas, J. B. Foresman, J. V Ortiz, J. Cioslowski, D. J. Fox, *Gaussian 09 Revision D.01*, Gaussian Inc., Wallingford CT, 2009; S. Grimme, J. Antony, S. Ehrlich, H. Krieg, *J. Chem. Phys.* **2010**, *132*, 154104.

S10 Gaussian09/D.01 jobs ran on a single 16-core (Intel Xeon E5-2640v3) node with 55 GB memory limit.

S11 (a) H. Kruse, S. Grimme, *J. Chem. Phys.* **2012**, *136*, 154101; (b) H. Cruse, L. Goerigk, S. Grimme, *J. Org. Chem.* **2012**, *77*, 10824–10834.
